# Dynamics of entangled networks of the quantum Internet

**DOI:** 10.1038/s41598-020-68498-x

**Published:** 2020-07-31

**Authors:** Laszlo Gyongyosi

**Affiliations:** 1 School of Electronics and Computer Science, University of Southampton, Southampton, SO17 1BJ UK; 2 Department of Networked Systems and Services, Budapest University of Technology and Economics, Budapest, 1117 Hungary; 3 MTA-BME Information Systems Research Group, Hungarian Academy of Sciences, Budapest, 1051 Hungary

**Keywords:** Mathematics and computing, Computer science, Pure mathematics

## Abstract

Entangled quantum networks are a fundamental of any global-scale quantum Internet. Here, a mathematical model is developed to quantify the dynamics of entangled network structures and entanglement flow in the quantum Internet. The analytical solutions of the model determine the equilibrium states of the entangled quantum networks and characterize the stability, fluctuation attributes, and dynamics of entanglement flow in entangled network structures. We demonstrate the results of the model through various entangled structures and quantify the dynamics.

## Introduction

As quantum computers continue to evolve significantly^1–18^, there arises a fundamental need for a communication network that provides unconditionally secure communication and all the network functions of the traditional internet. This novel network structure is called the quantum Internet^19–43^, a quantum communication network^20–23,25,27,31–36,38–42,44–76^ in which the nodes are represented by quantum devices (such as quantum repeaters^28,29,43,49,58,77–82^ or quantum computers^1–5, 83–86^), while the connections among the nodes are formulated via quantum entanglement. An entangled connection refers to a shared entangled quantum system among the quantum nodes^19,20,87,88–111^. Therefore, quantum entanglement is the key to any global-scale quantum Internet. Due to the fundamentally different processes and procedures associated with communication in the quantum Internet, the dynamic nature of these networks is also fundamentally different from a traditional network^77–81,112–124^. The dynamics^125–127^ involve the behavior of the network structure, which fluctuates along with the stability and reliability of the communication processes within the entangled structures. Quantifying the dynamics of the entangled structures allows us to determine the conditions for the development of stable quantum communications in strongly fluctuating and noisy environments, as well as to derive the basis for reliable and stable quantum communications in a global-scale quantum Internet^21,23,25,28,48–50^ setting. The quantum Internet is not yet available for experimentation, however, it must be ready for use as quantum computers become publicly available. Therefore, derivation of the fundamental dynamical attributes and behavioral characteristics of the entangled structures of the quantum Internet is fundamentally important and represents an emerging issue. While in a classical Internet a TCP/IP dynamics serves as an analytical tool to model the transmission, in a quantum Internet setting a dynamics model that characterizes the transmission of quantum states (density matrices) over the quantum channels is not available. A fundamental difference between the two settings, that in a quantum Internet the communication between distant points is realized over quantum channels (i.e., via CPTP—completely positive trace preserving—maps in a mathematical formalism), while the transmitted systems are entangled density matrices (assuming a general quantum Internet scenario). The correlation measure functions are also different in a quantum Internet setting, due to the fundamental nature of a classical communication channel and a quantum channel^128^.

Here, we develop an analytical model to quantify the dynamics of entangled network structures and entanglement flow in the quantum Internet. The analytical solutions of the model determine the equilibrium states of entangled quantum networks and characterize their stability and fluctuation attributes and the dynamics of entanglement flow within entangled network structures. Our work provides fundamental definitions and terms and proves fundamental theorems that quantify the dynamics of the entangled quantum networks of the quantum Internet. The proposed results are independent of the actual physical implementations; therefore, they can be applied within the heterogeneous structures of a global-scale quantum Internet.

To quantify the dynamic attributes of entangled structures of the quantum Internet, the analytical model defines a ${{\Psi }_{{{{{\mathscr {F}}}}_{N}}}}$ stability function motivated by the free energy thermodynamical potential function in thermondynamics^129–131^ and statistical physics^132–135^ (The free energy thermodynamical potential function $\Psi$ is defined as $\Psi = E-TS$, where *E* is the energy, *T* is an absolute temperature, while *S* is the entropy. The free energy thermodynamical potential function can also be interpreted as Gibbs free energy if *E* is interpreted as enthalpy^135^ (chemical reactions at constant pressure.) The concept of stability function $\Psi$ is therefore essentially roots in the Le Chatelier principle^136,137^ in a chemical equilibrium. The Le Chatelier principle says that chemical equilibrium occurs at minimum Gibbs energy of the reactants and the products and disturbance of the mix would result in restoration of the equilibrium in a way that cancels the perturbation.). In the developed model, the stability function determines the ${{{{\mathscr {S}}}}^{*}}\left( N \right)$ equilibrium state of the entangled structure. A ${{{\mathscr {S}}}^{*}}\left( N \right)$ stable equilibrium state of the entangled quantum network *N* is stable if heavy fluctuations in the network have zero effect on the entanglement flow ${{{\mathscr {F}}}_{N}}$ in the entangled quantum network. If ${{\Psi }_{{{{\mathscr {F}}}_{N}}}}$ is in a global minima, then the entangled structure is in a stable equilibrium state ${{{\mathscr {S}}}^{*}}\left( N \right)$. The determination of the stable equilibrium states of an entangled structure is fundamental to any seamless communication in a global-scale quantum Internet. The seamless quantum communication refers to a stable (reliable) transmission without fluctuations (the fluctuation does not exceed a critical limit). In a stable network state, the ${{R}_{x,y}}\left( {{t}_{0}},t \right)$ probability of non-erroneous information transmission between nodes and at moment is above a critical bound ${{C}^{*}}$, ${{R}_{x,y}}\left( {{t}_{0}},t \right) >{{C}^{*}}$, given that at moment ${{t}_{0}}$ the communication is correct. The entanglement flow is considered seamless optimal if it is seamless and if the entanglement rate exceeds a critical lower bound set for the entangled connections. We quantify the stability function for various entangled structures. The reliability of quantum communication is analyzed via the stability function of the entangled quantum network, since the stability of the entangled structure implies the reliability of quantum communication within the network.

Depending on the entanglement transmission rate of the entangled connections, the global quantum network can be decomposed into weakly and strongly entangled subnetworks. In a weakly entangled structure, the entanglement rate of the entangled connections is below a critical limit, while in a strongly entangled structure, the entanglement rate of the entangled connections exceeds this limit. As we prove, these structures are characterized by fundamentally different dynamic attributes and stability properties.

Entanglement purification is a cornerstone of the entangled networks of the quantum Internet^21,23,25,28,48,56,138–140^. Entanglement purification is a process that allows us to improve the entanglement fidelity of entangled states. It is a high-cost procedure since it requires the transmission of several quantum systems between the nodes to improve the final fidelity. Similar to the fundamental dynamic attributes of the quantum Internet, the dynamic effects of entanglement purification on an entangled structure remain unknown. We reveal the effects of entanglement purification on a large quantum network and show that the application of entanglement purification in a separated manner does not improve the capabilities of the quantum network.

The ${{{\mathscr {F}}}_{N}}$ entanglement flow in the entangled structure is the process of entanglement transmission in a large-scaled quantum entangled network *N*. Using the analytical model, we prove the conditions of seamless and seamless optimal entanglement transmission. The fluctuation of the entangled connections is derived via the Laplacian of the entangled structure, which is an important tool in spectral graph theory^141–144^.

The proposed analytical model also reveals the quantum supremacy (properties and attributes that are not available in a traditional internet) of the quantum Internet over the traditional internet. The proposed analytical solutions indicate that, for both weakly and strongly entangled structures, seamless optimal entanglement flow is always possible. Furthermore, the model revealed that an entangled structure can be transformed into a zero-fluctuation network via the establishment of a novel connection between the nodes, the result of which is proven via the use of spectral graph theory.

The novel contributions of our manuscript are as follows.

1. Dynamics of the entangled network structures of the quantum Internet is quantified in a closed-form. The fundamental definitions and terms are provided, fundamental theorems proven for entangled quantum networks.
2. We evaluate the stability of the entangled quantum networks of the quantum Internet and define the characteristics of weakly and strongly entangled structures.
3. We prove the stable equilibrium states of weakly and strongly entangled structures and quantify them in an exact closed form. We study the effects of noise on the stable equilibrium states of entangled structures.
4. We derive the fluctuation dynamics of entanglement transmission in the quantum Internet. Using the stable equilibrium states of entangled structures, we determine the conditions of seamless and seamless optimal entanglement flow in the quantum Internet.
5. We quantify the maximally allowed fluctuations in entangled structures for the seamless and seamless optimal entanglement flow in the quantum Internet. We prove the conditions for the construction of an entangled network structure with zero fluctuations.

This paper is organized as follows. Second section gives the basic terms and definitions. Third section  evaluates the dynamics and equilibrium states of entangled networks. Fourth section focuses on the dynamics of entanglement flow. Finally, fifth section concludes the results. Supplementary Information is included in the Appendix.

### Problem statement

The problems to be solved are as follows.

#### Problem 1

*Evaluate and quantify the dynamics and stability of an entangled network structure in an exact closed form. Prove the equilibrium state and fluctuation dynamics of the entangled network structures of the quantum Internet. Determine the effects of noise on the equilibrium states of the entangled network.*

#### Problem 2

*Prove the attributes of weakly and strongly entangled structures of the quantum Internet. Determine the stable equilibrium states of the entangled network structures for both noiseless and noisy cases and for both weakly and strongly entangled structures.*

#### Problem 3

*Prove the dynamic effects of local entanglement purification in the quantum Internet.*

#### Problem 4

*Prove the maximally allowed fluctuations in entangled structures for seamless entanglement flow in the quantum Internet.*

#### Problem 5

*Determine the attributes of an entangled network structure that statistically leads to zero fluctuations.*

The resolutions to Problems 1–5 are proposed in the Theorems and Lemmas of the manuscript.

## Preliminaries

This section briefly summarizes the basic terms and definitions. For further details, we suggest^28,128^.

### Entanglement fidelity

Let $| \beta _{00} \rangle = { \frac{1}{\sqrt{2} }} \left( {\left| 00 \right\rangle } +{\left| 11 \right\rangle } \right)$ be the target Bell state subject to be generated between distant nodes *A* and *B*^145^. The entanglement fidelity *F* at a given shared system $\sigma$ between *A* and *B* is

1 $$\begin{aligned} F\left( \sigma \right) =\langle {{\beta }_{00}} | \sigma |{{\beta }_{00}} \rangle , \end{aligned}$$

such that $F=1$ for a perfect Bell state and $F<1$ for an imperfect state^28,145^.

### Entanglement levels

Let *V* refer to the nodes of an entangled quantum network *N*, which consists of a transmitter node $A\in V$, a receiver node $B\in V$, and quantum repeater nodes $R_i\in V$, $i=1,\dots ,q$^145^. Let $E=\left\{ E_j\right\}$, $j=1,\dots ,m$ refer to a set of edges between the nodes of *V*, where each $E_j$ identifies an $\text {L}_l$-level entanglement, $l=1,\dots ,r$, between quantum nodes $x_j$ and $y_j$ , respectively. In the doubling architecture^28^, the number of spanned nodes is doubled in each level of entanglement swapping. The $d{\left( x,y\right) }_{\text {L}_l}$ hop distance for an *l*-level entangled connection $\text {L}_l$-level between nodes $x,y\in V$ is^51^

2 $$\begin{aligned} d{\left( x,y\right) }_{\text {L}_l}=2^{l-1}, \end{aligned}$$

where $l=1$ refers to a direct connection between *x* and *y* with no intermediate quantum repeaters^145^.

### Entanglement throughput, entanglement purification, entanglement swapping

#### Entanglement throughput

The $B_{F} \left( E_{l} \left( x,y\right) \right)$ entanglement throughput of an *l*-level entangled connection $E_{l} \left( x,y\right)$ is a quantity that measures the number of entangled density matrices transmittable over $E_{l} \left( x,y\right)$ per a unit time $\pi _{S} =st_{C}$, where *s* is a nonzero real number, $s>0$, of a particular entanglement fidelity *F*, where *C* is a cycle (see “Dynamics of the entangled structure” section). (Since $E_{l} \left( x,y\right)$ is formulated via a set of ${{\mathscr {N}}}$ physical links, it abstracts the capabilities of the physical links of $E_{l} \left( x,y\right)$ and the efficiency of entanglement swapping in the nodes). Practically, the entangled states are realized via Bell states in current implementations^145^ (The $B_{F}$ entanglement throughput is related to the term “bandwidth” from classical communication theory. A fundamental difference that a quantum channel ${{\mathscr {N}}}$ can transmit several different correlations, such as classical, private classical and quantum correlation^128^, and the quantum repeaters generate and outputs entangled density matrices (halves of an EPR states in practice) to establish an *l*-level entangled connection (see (2)). Quantum entanglement is a quantum correlation, therefore the term “bandwidth” is related to the $Q\left( {\mathscr {N}} \right)$ quantum capacity^128,146^ of the quantum channel ${{\mathscr {N}}}$. In a classical setting only classical correlations can be transmitted over a classical channel *N*, therefore the “bandwidth” in a traditional interpretation is related to the *C*(*N*) classical capacity of *N*.).

#### Entanglement purification

The $P_{N}$ entanglement purification process^28,56,138–140^ takes two imperfect systems $\sigma _{1}$ and $\sigma _{2}$ with $F_0<1$, and outputs a higher-fidelity system $\rho$ such that

3 $$\begin{aligned} F\left( \rho \right) >F_0. \end{aligned}$$

For a detailed technical description of entanglement purification, we suggest^28^.

#### Entanglement swapping

The entanglement swapping operation splices two short-distance Bell states into a longer-distance Bell pair via operations applied in an intermediate quantum node and via classical side information (i.e., a similar mechanism to quantum teleportation^28,145^).

### Definitions

The dynamics terms utilized in the model are defined as follows. The aim of the definitions is to introduce the related quantities, the detailed definitions are given in the particular sections.

#### Entanglement flow

##### **Definition 1**

(*Entanglement flow*) The ${{\mathscr {F}}}_{N}$ entanglement flow is the entanglement transmission over the $\left| V\right|$ quantum repeaters of the physical network *N*. For a given *j*th entangled path ${{\mathscr {P}}}_{j}$ of ${{\mathscr {F}}}_{N}$, $j=1,\ldots ,Q$, where *Q* is the total number of paths in *N*, an *i*th quantum node $R_{i}$, $i=1,\ldots ,\left| V\right|$, outputs *n* density matrices on path ${{\mathscr {P}}}_{j}$. For the total *Q* paths of *N*, an *i*th quantum repeater $R_{i}$ outputs $D\ge n$ density matrices on the *Q* paths ${{\mathscr {P}}}_{1},\ldots ,{{\mathscr {P}}}_{Q}$.

Entanglement flow is the number of entangled density matrices (half of EPR pairs in a practical setting) generated and outputted by the quantum repeaters, see also “Average entanglement rate of an entanglement flow” section. In the entanglement distribution procedure, a given quantum repeater $R_{i}$ has a particular number of incoming density matrices (halves of EPR states received from source neighbor quantum nodes in a practical scenario) and outcoming density matrices (the given quantum repeater $R_{i}$ generates entangled states, and sends out one half of the EPR states to a destination node, see also “Entanglement throughput” section.) The terminology “outputs a state into a path” means that a particular output state of the quantum repeater belongs to a particular path. Other outputs belong to other paths, etc.

#### Stability of the entangled quantum network

##### **Definition 2**

(*Stability function*) The $\Psi _{{{\mathscr {F}}}_{N} }\in {\mathbb {R}}$ stability function (will be detailed in (20)) measures the effects of any network fluctuation and noise on the entangled structure and the entanglement flow. If $\Psi _{{{\mathscr {F}}}_{N} }$ is in a local minima, then the entangled structure is in an ${{\mathscr {S}}}^{*} \left( N\right)$ equilibrium state. If $\Psi _{{{\mathscr {F}}}_{N} }$ is in a global minima, then the entangled structure is in a stable equilibrium state ${{\mathscr {S}}}^{*} \left( N\right)$.

#### Equilibrium state of an entangled quantum network

##### **Definition 3**

(*Equilibrium state of the quantum network*) The ${{\mathscr {S}}}^{*} \left( N\right)$ equilibrium state of the entangled quantum network *N* is a state of the entangled structure in which the network structure keeps the ${{\mathscr {S}}}^{*} \left( N\right)$ network state at $\vec {\varphi }\left( N\right) =\left( \varphi \left( 1\right) ,\ldots ,\varphi \left( \left| V\right| \right) \right) ^{T}$ fluctuations of the quantum network, where $\varphi \left( i \right) \in {\mathbb {R}}$ is a (normalized) fluctuation of a node ${{R}_{i}}$, defined as

4 $$\begin{aligned} \varphi \left( i \right) =\tfrac{1}{B_{F}^{*}\left( {{{\mathscr {F}}}_{N}} \right) }\left( {{B}_{F}}\left( {{R}_{i}} \right) -B_{F}^{*}\left( {{{\mathscr {F}}}_{N}} \right) \right) , \end{aligned}$$

where ${{B}_{F}}\left( i \right)$ is the outcoming entanglement throughput of node ${{R}_{i}}$ (number of density matrices – half of EPR pairs in a practical setting – outputted by ${{R}_{i}}$), while $B_{F}^{*}\left( {{{\mathscr {F}}}_{N}} \right)$ is a critical lower bound for the entanglement throughput of ${{{\mathscr {F}}}_{N}}$. The state of the quantum network is detailed in “State of the quantum network” section, see also (21).

**Stable equilibrium state**

##### **Definition 4**

(*Stable equilibrium state of the quantum network*) The ${{\mathscr {S}}}^{*} \left( N\right)$ equilibrium state of the entangled quantum network is stable if heavy fluctuations, $\varphi \left( i \right) >{{\varphi }^{*}}$, where ${{\varphi }^{*}}\in {\mathbb {R}}$ is a critical bound on network fluctuation ${\varphi }$ set for the $i=1,\ldots ,\left| V \right|$ nodes of *N*, keeps the network state ${{\mathscr {S}}}^{*} \left( N\right)$ (Detailed definition of ${\varphi }$ is given in (45).).

#### Average entanglement fidelity of an entanglement flow

For a *j*th path ${{\mathscr {P}}}_{j}$, the function $F_{{{\mathscr {P}}}_{j} } \left( R_{i} \right)$ identifies the average (Note: an averaging of quantities is used by the statistical model of the quantum network.) entanglement fidelity output via the quantum repeater $R_{i}$ in the ${{\mathscr {F}}}_{N}$ entanglement flow of *N*, as

5 $$\begin{aligned} F_{{{\mathscr {P}}}_{j} } \left( R_{i} \right) ={ \frac{1}{n}} \sum _{f=1}^{n}F_{i} \left( \sigma _{f} \right) , \end{aligned}$$

where $\sigma _{f}$ is an *f*th, $f=1,\ldots ,n$, entangled subsystem outputted by $R_{i}$ on path ${{\mathscr {P}}}_{j}$. For the *Q* paths of *N*, the $F_{{{\mathscr {P}}}} \left( R_{i} \right)$ fidelity is derived for $R_{i}$ as

6 $$\begin{aligned} F_{{{\mathscr {P}}}} \left( R_{i} \right) ={ \frac{1}{D_{i} }} \sum _{f=1}^{D_{i} }F_{i} \left( \sigma _{f} \right) , \end{aligned}$$

where $D_i$ is the number of density matrices outputted to the *Q* paths ${{\mathscr {P}}}_{1},\ldots ,{{\mathscr {P}}}_{Q}$ by $R_i$. From (6), the $F\left( {{\mathscr {F}}}_{N} \right)$ average fidelity of ${{\mathscr {F}}}_{N}$ is as

7 $$\begin{aligned} F\left( {{\mathscr {F}}}_{N} \right) ={ \frac{1}{\left| V\right| }} \sum _{i=1}^{\left| V\right| }F_{{{\mathscr {P}}}} \left( R_{i} \right) ={ \frac{1}{\left| V\right| }} \sum _{i=1}^{\left| V\right| }{ \frac{1}{D_{i} }}\sum _{f=1}^{D_{i} }F_{i} \left( \sigma _{f} \right) . \end{aligned}$$

#### Average entanglement rate of an entanglement flow

For a *j*th path ${{\mathscr {P}}}_{j}$ of ${{\mathscr {F}}}_{N}$ with $\left| V_{{{\mathscr {P}}}_{j} } \right|$ quantum nodes and $\left| S_{{{\mathscr {P}}}_{j} } \right|$ entangled connections, the $B_{F,{{\mathscr {P}}}_{j} } \left( {{\mathscr {F}}}_{N} \right)$ average entanglement rate of ${{\mathscr {P}}}_{j}$ at a particular entanglement fidelity *F* is

8 $$\begin{aligned} B_{F,{{\mathscr {P}}}_{j} } \left( {{\mathscr {F}}}_{N} \right) ={ \frac{1}{\left| S_{{{\mathscr {P}}}_{j} } \right| }} \sum _{s=1}^{\left| S_{{{\mathscr {P}}}_{j} } \right| }B_{F,{{\mathscr {P}}}_{j} } \left( E_{s} \right) , \end{aligned}$$

where $B_{F,{{\mathscr {P}}}_{j} } \left( E_{s} \right)$ identifies the average entanglement throughput of an *s*th entangled connection $E_{s}$ for a particular entanglement fidelity *F*, $s=1,\ldots ,\left| S_{{{\mathscr {P}}}_{j} } \right|$.

For the total *Q* paths of *N*, the $B\left( {{\mathscr {F}}}_{N} \right)$ average entanglement throughput of ${{\mathscr {F}}}_{N}$ for a particular entanglement fidelity *F* is as

9 $$\begin{aligned} B_F\left( {{\mathscr {F}}}_{N} \right) ={ \frac{1}{Q}} \sum _{j=1}^{Q}{ \frac{1}{\left| S_{{{\mathscr {P}}}_{j} } \right| }}\sum _{s=1}^{\left| S_{{{\mathscr {P}}}_{j} } \right| }B_{F,{{\mathscr {P}}}_{j} } \left( E_{s} \right) . \end{aligned}$$

#### Average noise of an entanglement flow

The $0\le \Delta \left( {{\mathscr {F}}}_{N} \right) \le 1$ average (Note: an averaging of quantities is used by the statistical model of the quantum network.) noise probability (referred to as average noise) of ${{\mathscr {F}}}_{N}$ is defined as

10 $$\begin{aligned} \Delta \left( {{\mathscr {F}}}_{N} \right) ={ \frac{1}{\left| V\right| }} \sum _{i=1}^{\left| V\right| }\Delta \left( R_{i} \right) ={ \frac{1}{\left| V\right| }} \sum _{i=1}^{\left| V\right| }{ \frac{1}{D_{i} }}\sum _{f=1}^{D_{i} }\Delta _{i} \left( \sigma _{f} \right) , \end{aligned}$$

where $0\le \Delta \left( R_{i} \right) \le 1$ is the average noise of an *i*th quantum node $R_{i}$,

11 $$\begin{aligned} \Delta \left( R_{i} \right) ={ \frac{1}{D_{i} }} \sum _{f=1}^{D_{i} }\Delta _{i} \left( \sigma _{f} \right) , \end{aligned}$$

where $0\le \Delta _{i}\left( \sigma _{f} \right) \le 1$ is the noise probability on an *f*th output density matrix $\sigma _{f}$ of $R_{i}$, defined as

12 $$\begin{aligned} {{\mathscr {E}}\left( {{\sigma }_{f}} \right) }=\left( 1-{{\Delta }_{i}}\left( {{\sigma }_{f}} \right) \right) {{\sigma }_{f}}+{{\Delta }_{i}}\left( {{\sigma }_{f}} \right) {{{\sigma }'_{f}}}, \end{aligned}$$

where ${{\mathscr {E}}}$ is a noisy channel, while ${{{\sigma }'_{f}}}$ is the noisy density matrix with an arbitrary noise, defined as

13 $$\begin{aligned} {{{\sigma }'_{f}}}={U_{e}^{f}}{{\sigma }_{f}}(U_{e}^{f})^{\dagger }, \end{aligned}$$

where $U_{e}^{f}$ is an error transformation.

#### State of the quantum network

Let ${{\mathscr {S}}}\left( N\right)$ refer to the state (statistical model) of *N*, as

14 $$\begin{aligned} {{\mathscr {S}}}\left( N\right) =\left\{ f \left( B_{F} \left( {{\mathscr {F}}}_{N} \right) \right) ,\Delta \left( {{\mathscr {F}}}_{N} \right) ,\phi \left( F\left( {{\mathscr {F}}}_{N} \right) \right) \right\} , \end{aligned}$$

where $f \left( B_{F} \left( {{\mathscr {F}}}_{N} \right) \right)$ is a normalized value of $B_{F} \left( {{\mathscr {F}}}_{N} \right)$ (see (9)), $\Delta \left( {{\mathscr {F}}}_{N} \right)$ is given in (10), while $\phi \left( F\left( {{\mathscr {F}}}_{N} \right) \right)$ is a normalized value of $F\left( {{\mathscr {F}}}_{N} \right)$ (see (7)).

#### Seamless property and optimality of an entanglement flow

**Seamless entanglement flow**

##### **Definition 5**

(*Seamless property of entanglement flow*) An ${{\mathscr {F}}}_{N}$ entanglement flow is seamless, ${{\mathscr {F}}}_{N} =\tilde{{{\mathscr {F}}}}_{N}$, if for all $\left| V \right|$ nodes of *N*

15 $$\begin{aligned} \varphi \left( i \right) \le \varphi ^{*}, \end{aligned}$$

where $\varphi ^{*}$ is a critical bound on network fluctuation ${\varphi }$ (see (45)) set for the $i=1,\ldots ,\left| V \right|$ nodes of *N*.

**Seamless optimal entanglement flow**

##### **Definition 6**

(*Seamless optimal entanglement flow*) An ${{\mathscr {F}}}_{N}$ entanglement flow is seamless optimal, ${{\mathscr {F}}}_{N} ={{\mathscr {F}}}_{N}^{*}$, if ${{\mathscr {F}}}_{N}$ is seamless, ${{\mathscr {F}}}_{N} =\tilde{{{\mathscr {F}}}}_{N}$, and

16 $$\begin{aligned} B_{F} \left( {{\mathscr {F}}}_{N} \right) \ge B'_{F} \left( {{\mathscr {S}}}^{*} \left( N\right) \right) , \end{aligned}$$

where $B'_{F} \left( {{\mathscr {S}}}^{*} \left( N\right) \right)$ is a lower bound on $B_{F} \left( {{\mathscr {S}}}^{*} \left( N\right) \right)$ in a ${{\mathscr {S}}}^{*} \left( N\right)$ stable equilibrium state of *N*.

#### Dynamics of the entangled structure

**Weakly entangled quantum networks**

##### **Definition 7**

(*Weakly entangled subnetwork of the entangled quantum network*) Let ${{\mathscr {S}}}_{N}$ be a subnetwork of *N* with $\left| {{\mathscr {S}}}_{N} \right|$ quantum nodes. The ${{\mathscr {S}}}_{N}$ subnetwork is weakly entangled, ${{\mathscr {S}}}'_{N}$, if only

17 $$\begin{aligned} B_{F} \left( {{\mathscr {S}}}'_{N} \right) <B_{F}^{*} \left( {{\mathscr {S}}}'_{N} \right) , \end{aligned}$$

holds for the $B_{F} \left( {{\mathscr {S}}}'_{N} \right)$ average entanglement throughput of ${{\mathscr {S}}}'_{N}$ for a particular entanglement fidelity *F*,

18 $$\begin{aligned} B_F\left( {{\mathscr {F}}}_{N} \right) ={ \frac{1}{\Omega _{{{\mathscr {S}}}'_{N} }}} \sum _{j=1}^{\Omega _{{{\mathscr {S}}}'_{N} }}{ \frac{1}{\left| S_{{{\mathscr {P}}}_{j} } \right| }}\sum _{s=1}^{\left| S_{{{\mathscr {P}}}_{j} } \right| }B_{F,{{\mathscr {P}}}_{j} } \left( E_{s} \right) . \end{aligned}$$

where $\Omega _{{{\mathscr {S}}}'_{N} }$ is the number of paths of ${{\mathscr {S}}}'_{N}$, while $B_{F}^{*} \left( {{\mathscr {S}}}'_{N} \right)$ is an expected value of $B_{F} \left( {{\mathscr {S}}}'_{N} \right)$ for a particular entanglement fidelity *F*.

**Strongly entangled quantum networks**

##### **Definition 8**

(*Strongly entangled subnetwork of the entangled quantum network*) The ${{\mathscr {S}}}_{N}$ subnetwork of *N* is strongly entangled, ${{\mathscr {S}}}_{N}^{*}$, if only

19 $$\begin{aligned} B_{F} \left( {{\mathscr {S}}}_{N}^{*} \right) \ge B_{F}^{*} \left( {{\mathscr {S}}}_{N}^{*} \right) , \end{aligned}$$

for a particular entanglement fidelity *F*.

**Cycle**

##### **Definition 9**

A cycle *C* with cycle-time ${{t}_{C}}={1}/{{{f}_{C}}} \sec$ is set via an oscillator $O_{C}$ with frequency ${{f}_{C}}={1}/{{{t}_{C}}}$ in the quantum nodes used for synchronization of a quantum network.

The *sC* cycles identify $s{{t}_{C}}={s}/{{{f}_{C}}} \sec$, where *s* is a nonzero real number.

## Dynamics and equilibrium states of entangled networks

### Stability of an entangled quantum network

#### Theorem 1

(Dynamics of the entangled network structure) *The* $\Psi _{{{\mathscr {F}}}_{N}}$ stability function defines the stability of the entangled structure N as

20 $$\begin{aligned} \Psi _{{{\mathscr {F}}}_{N} } \left( \phi \left( F\left( {{\mathscr {F}}}_{N} \right) \right) \right) =\left| V\right| c_{B} \varphi \left( \chi \left( {{\mathscr {F}}}_{N} \right) \right) , \end{aligned}$$

*where* $F\left( {{\mathscr {F}}}_{N} \right)$ *is the average fidelity of* ${{\mathscr {F}}}_{N}$, $\phi \left( \cdot \right)$ *is a normalizing function*, $c_{B}$ *and* $\varphi$ *are constants, while* $\chi \left( {{\mathscr {F}}}_{N} \right)$ *is statistical quantity determined via* $\Delta \left( {{\mathscr {F}}}_{N} \right) ,\phi \left( F\left( {{\mathscr {F}}}_{N} \right) \right)$ *and* $F\left( {{\mathscr {F}}}_{N} \right)$.

#### Proof

The proof is purely statististical, defines the stability function motivated by the terminology of free energy potential, showing how the network state evolves where a challenge is evaluating the Chapman–Kolmogorov equation, that will be defined in (48).

Let ${{\mathscr {S}}}\left( N\right)$ refer to the state of *N* as given by (14). Then, a ${{\mathscr {S}}}^{*} \left( N\right)$ stable equilibrium state of (14) is defined as

21 $$\begin{aligned} {{\mathscr {S}}}^{*} \left( N\right) =\left\{ f^{*} \left( B_{F} \left( {{\mathscr {F}}}_{N} \right) \right) ,\Delta ^{*} \left( {{\mathscr {F}}}_{N} \right) ,\phi ^{*} \left( F\left( {{\mathscr {F}}}_{N} \right) \right) \right\} , \end{aligned}$$

where $*$ refers to the function values in ${{\mathscr {S}}}^{*} \left( N\right)$.

The formalization of (14) is plausible model for the fluctuation dynamics analysis, since the entangled network structure formulates a macroscopic system with local interactions^125,126^. In our analytical model, the local interactions are represented by a normalized value of the $B_{F} \left( {{\mathscr {F}}}_{N} \right)$ average entanglement rate of entanglement flow, while the global parameter is the $\Delta \left( {{\mathscr {F}}}_{N} \right)$ average noise of entanglement flow in the entangled structure. Another important parameter of the statistical model is the order parameter, which represents the statistical orderliness of the system. In the statistical physics model of the entangled quantum network structure, the orderliness of the system is represented by a normalized value of the $F\left( {{\mathscr {F}}}_{N} \right)$ average fidelity of the entanglement flow in the quantum network.

The $\phi \left( \cdot \right)$ and $f \left( \cdot \right)$ normalizing functions are defined as follows.

The $\phi \left( F\left( {{\mathscr {F}}}_{N} \right) \right)$ normalized value of $F\left( {{\mathscr {F}}}_{N} \right)$ (see (7)) is defined as

22 $$\begin{aligned} \phi \left( F\left( {{\mathscr {F}}}_{N} \right) \right) =F\left( {{\mathscr {F}}}_{N} \right) {\tilde{\xi }}\left( {{\mathscr {F}}}_{N} \right) , \end{aligned}$$

where $-1\le {\tilde{\xi }}\left( {{\mathscr {F}}}_{N} \right) \le 1$ identifies the ratios of quantum repeaters in *N* for which the $F_{{{\mathscr {P}}}} \left( R_{i} \right)$ average fidelity (see (6)) is $F_{{{\mathscr {P}}}} \left( R_{i} \right) <F_{{{\mathscr {P}}}}^{*} \left( R_{i} \right)$ and $F_{{{\mathscr {P}}}} \left( R_{i} \right) \ge F_{{{\mathscr {P}}}}^{*} \left( R_{i} \right)$, where $F_{{{\mathscr {P}}}}^{*} \left( R_{i} \right)$ is a lower bound on $F_{{{\mathscr {P}}}} \left( R_{i} \right)$. See also (29) and (33) for a detailed definition of ${\tilde{\xi }}\left( {{\mathscr {F}}}_{N} \right)$.

The $f \left( B_{F} \left( {{\mathscr {F}}}_{N} \right) \right)$ normalized value of $B_{F} \left( {{\mathscr {F}}}_{N} \right)$ is defined as

23 $$\begin{aligned} f \left( B_{F} \left( {{\mathscr {F}}}_{N} \right) \right) =B_{F} \left( {{\mathscr {F}}}_{N} \right) { \frac{1}{B_{F}^{*} \left( {{\mathscr {F}}}_{N} \right) }} , \end{aligned}$$

where $B_{F}^{*} \left( {{\mathscr {F}}}_{N} \right)$ is a critical bound for $B_{F} \left( {{\mathscr {F}}}_{N} \right)$.

The function in (23) can be characterized as

24 $$\begin{aligned} f \left( B_{F} \left( {{\mathscr {F}}}_{N} \right) \right) =\left\{ \begin{array}{l} {f \left( B_{F} \left( {{\mathscr {F}}}_{N} \right) \right)<1,\; \mathrm{if}\; B_{F} \left( {{\mathscr {F}}}_{N} \right) \mathrm{<}B_{F}^{*} \left( {{\mathscr {F}}}_{N} \right) } \\ {f \left( B_{F} \left( {{\mathscr {F}}}_{N} \right) \right) =1,\; \mathrm{if}\; B_{F} \left( {{\mathscr {F}}}_{N} \right) \mathrm{=}B_{F}^{*} \left( {{\mathscr {F}}}_{N} \right) } \\ {f \left( B_{F} \left( {{\mathscr {F}}}_{N} \right) \right)>1,\; \mathrm{if}\; B_{F} \left( {{\mathscr {F}}}_{N} \right) \mathrm{>}B_{F}^{*} \left( {{\mathscr {F}}}_{N} \right) } \end{array}\right. . \end{aligned}$$

Using the statistical physics model ${{\mathscr {S}}}\left( N\right)$ of (14), let $H\left( {{\mathscr {F}}}_{N} \right)$ be the Hamiltonian of the entanglement flow ${{\mathscr {F}}}_{N}$ in the entangled network structure *N*, as

25 $$\begin{aligned} H\left( {{\mathscr {F}}}_{N} \right) =-H\left( \Delta \left( {{\mathscr {F}}}_{N} \right) \right) \sum _{i\in V}\sigma _{i} -\sum _{E\left( i,k\right) \in S}J_{i,k} \sigma _{i} \sigma _{k} , \end{aligned}$$

where $J_{i,k}$ is an interaction parameter, while $H\left( \Delta \left( {{\mathscr {F}}}_{N} \right) \right)$ is the Hamiltonian^125,126^ of the average noise $\Delta \left( {{\mathscr {F}}}_{N} \right)$ (see (10)), as

26 $$\begin{aligned} H\left( \Delta \left( {{\mathscr {F}}}_{N} \right) \right) ={ \frac{1}{\mu _{0} }} \Delta \left( {{\mathscr {F}}}_{N} \right) c_{B} \varphi , \end{aligned}$$

where $\mu _{0}$ is a normalization term defined via $J_{i,k}$^125,126^, as

27 $$\begin{aligned} {{J}_{i,k}}=\tfrac{1}{\mu _{0}^{2}}\left( \begin{matrix} J &{} J \\ -J &{} J \\ \end{matrix} \right) , \end{aligned}$$

while $c_{B}$ and $\varphi$ constants, while $\sigma _{i}$ represents a state of quantum node $R_{i} \in V$ of *N*, $i=1,\ldots ,\left| V\right|$, defined as

28 $$\begin{aligned} \sigma _{i} ={{\xi }_{i}}\mu _{0} , \end{aligned}$$

where ${{\xi }_{i}}$ is as

29 $$\begin{aligned} {{\xi }_{i}}=\mathrm{sign}\left( \Delta F_{{{\mathscr {P}}}} \left( R_{i} \right) \right) , \end{aligned}$$

where the $\mathrm{sign}\left( x\right)$ function returns the sign of *x* ($\mathrm{sign}\left( 0\right)$ is considered as negative), $\Delta F_{{{\mathscr {P}}}} \left( R_{i} \right)$ is as

30 $$\begin{aligned} \Delta F_{{{\mathscr {P}}}} \left( R_{i} \right) =F_{{{\mathscr {P}}}}^{*} \left( R_{i} \right) -F_{{{\mathscr {P}}}} \left( R_{i} \right) , \end{aligned}$$

The $H\left( {{\mathscr {F}}}_{N} \right)$ Hamiltonian of ${{\mathscr {F}}}_{N}$ from (25) can be rewritten as

31 $$\begin{aligned} H\left( {{\mathscr {F}}}_{N} \right) =-H\left( \Delta \left( {{\mathscr {F}}}_{N} \right) \right) \mu _{0} \sum _{i}{{\xi }_{i}} -J\sum _{i,k}{{\xi }_{i}}{{\xi }_{k}} . \end{aligned}$$

The result in (31) is equivalent to an Ising system^125–127^ in statistical physics, while in some physical models ${\xi }_{i}, {\xi }_{j}$ can also refer to spin up/down of qubits *i*, *j*. In the current system model, these parameters refer to the state of quantum nodes in terms of quantum fidelity, see (29) and (30).

From some fundamentals of statistical physics^125–127^, the $H\left( {{\xi }_{i}}\right)$ Hamiltonian of (29) can be derived in the following manner. Let ${{\xi }_{i}}$ and ${{\xi }_{k}}$ be associated to $R_{i}$ and $R_{k}$, as given in (29). Then, by utilizing the Weiss mean field^147^ approximation (The Weiss mean field theory is the mean field theory of an Ising model^147^.), ${{{\xi }_{i}}}{{{\xi }_{k}}}$ can be evaluated as

32 $$\begin{aligned} {{{\xi }_{i}}}{{{\xi }_{k}}}&=\left( {{{\xi }_{i}}}-{\tilde{\xi }}\left( {{{\mathscr {F}}}_{N}} \right) \right) \left( {{{\xi }_{k}}}-{\tilde{\xi }}\left( {{{\mathscr {F}}}_{N}} \right) \right) -{{\left( {\tilde{\xi }}\left( {{{\mathscr {F}}}_{N}} \right) \right) }^{2}}+\left( {\tilde{\xi }}\left( {{{\mathscr {F}}}_{N}} \right) \right) \left( {{{\xi }_{i}}}+{{{\xi }_{k}}} \right) \\&\approx {{\left( {\tilde{\xi }}\left( {{{\mathscr {F}}}_{N}} \right) \right) }^{2}}+\left( {\tilde{\xi }}\left( {{{\mathscr {F}}}_{N}} \right) \right) \left( {{{\xi }_{i}}}+{{{\xi }_{k}}} \right) , \end{aligned}$$

where ${\tilde{\xi }}\left( {{\mathscr {F}}}_{N} \right)$ is defined as

33 $$\begin{aligned} {\tilde{\xi }}\left( {{\mathscr {F}}}_{N} \right) ={ \frac{1}{\left| V\right| }} \sum _{i=1}^{\left| V\right| }{{\xi }_{i}} . \end{aligned}$$

As follows, (22) can be rewritten as a statistical quantity, as

34 $$\begin{aligned} \phi \left( F\left( {{\mathscr {F}}}_{N} \right) \right) ={ \frac{F\left( {{\mathscr {F}}}_{N} \right) }{\left| V\right| }} \sum _{i=1}^{\left| V\right| }{{\xi }_{i}} , \end{aligned}$$

and the range of (34) can be characterized as

35 $$\begin{aligned} \phi \left( F\left( {{\mathscr {F}}}_{N} \right) \right) =\left\{ \begin{array}{l} {-1\le \phi \left( F\left( {{\mathscr {F}}}_{N} \right) \right)<0,\; {\mathrm{if}}\; \left( -1\le {\tilde{\xi }}\left( {{\mathscr {F}}}_{N} \right)<0\right) \wedge \left( F\left( {{\mathscr {F}}}_{N} \right)>0\right) } \\ {\phi \left( F\left( {{\mathscr {F}}}_{N} \right) \right) =0,\; {\mathrm{if}}\; \left( {\tilde{\xi }}\left( {{\mathscr {F}}}_{N} \right) =0\right) \wedge \left( F\left( {{\mathscr {F}}}_{N} \right) \ge 0\right) } \\ {0<\phi \left( F\left( {{\mathscr {F}}}_{N} \right) \right) \le 1,\; {\mathrm{if} }\;\left( 0<{\tilde{\xi }}\left( {{\mathscr {F}}}_{N} \right) \le 1\right) \wedge \left( F\left( {{\mathscr {F}}}_{N} \right) >0\right) } \end{array}\right. . \end{aligned}$$

From the relations (32) and (33), the Hamiltonian $H\left( {{\mathscr {F}}}_{N} \right)$ from (31) can be rewritten as

36 $$\begin{aligned} H\left( {{{\mathscr {F}}}_{N}} \right)&=-H\left( \Delta \left( {{{\mathscr {F}}}_{N}} \right) \right) {{\mu }_{0}}\sum \limits _{i\in V}{{{{\xi }_{i}}}}-J\sum \limits _{E\left( i,k \right) \in S}{\left( -{{\left( {\tilde{\xi }}\left( {{{\mathscr {F}}}_{N}} \right) \right) }^{2}}+{\tilde{\xi }}\left( {{{\mathscr {F}}}_{N}} \right) \left( {{{\xi }_{i}}}+{{{\xi }_{k}}} \right) \right) } \\&=-H\left( \Delta \left( {{{\mathscr {F}}}_{N}} \right) \right) {{\mu }_{0}}\left| V \right| {\tilde{\xi }}\left( {{{\mathscr {F}}}_{N}} \right) -J\left( -{{\left( {\tilde{\xi }}\left( {{{\mathscr {F}}}_{N}} \right) \right) }^{2}}\sum \limits _{E\left( i,k \right) \in S}{1}+{\tilde{\xi }}\left( {{{\mathscr {F}}}_{N}} \right) \sum \limits _{E\left( i,k \right) \in S}{\left( {{{\xi }_{i}}}+{{{\xi }_{k}}} \right) } \right) \\&=-H\left( \Delta \left( {{{\mathscr {F}}}_{N}} \right) \right) {{\mu }_{0}}\left| V \right| {\tilde{\xi }}\left( {{{\mathscr {F}}}_{N}} \right) -J\left( -{{\left( {\tilde{\xi }}\left( {{{\mathscr {F}}}_{N}} \right) \right) }^{2}}\tfrac{1}{2}{\tilde{K}}\left| V \right| +{\tilde{\xi }}\left( {{{\mathscr {F}}}_{N}} \right) {\tilde{K}}\sum \limits _{i\in V}{{{{\xi }_{i}}}} \right) \\&=-H\left( \Delta \left( {{{\mathscr {F}}}_{N}} \right) \right) {{\mu }_{0}}\left| V \right| {\tilde{\xi }}\left( {{{\mathscr {F}}}_{N}} \right) -J\left( -{{\left( {\tilde{\xi }}\left( {{{\mathscr {F}}}_{N}} \right) \right) }^{2}}\tfrac{1}{2}{\tilde{K}}\left| V \right| +{{\left( {\tilde{\xi }}\left( {{{\mathscr {F}}}_{N}} \right) \right) }^{2}}{\tilde{K}}\left| V \right| \right) \\&=-\left| V \right| \left( -H\left( \Delta \left( {{{\mathscr {F}}}_{N}} \right) \right) {{\mu }_{0}}{\tilde{\xi }}\left( {{{\mathscr {F}}}_{N}} \right) +\tfrac{1}{2}J{\tilde{K}}{{\left( {\tilde{\xi }}\left( {{{\mathscr {F}}}_{N}} \right) \right) }^{2}} \right) , \end{aligned}$$

where ${\tilde{K}}$ is the average number of entangled connections between the nodes, defined as

37 $$\begin{aligned} {\tilde{K}}={ \frac{1}{J}} c_{B} \varphi f \left( B_{F} \left( {{\mathscr {F}}}_{N} \right) \right) , \end{aligned}$$

where $f \left( B_{F} \left( {{\mathscr {F}}}_{N} \right) \right)$ is given in (23), while

38 $$\begin{aligned} \sum _{E\left( i,k\right) \in S}1 ={ \frac{1}{2}} {\tilde{K}}\left| V\right| , \end{aligned}$$

since the term ${\tilde{K}}\left| V\right|$ takes twice the entangled connections of *N*.

Since $H\left( {{\xi }_{i}}\right)$ is derived for the $\left| V\right| =1$ and $\left| S\right| =K$ case, from (36), the Hamiltonian $H\left( {{\xi }_{i}}\right)$ of ${{\xi }_{i}}$ is as

39 $$\begin{aligned} H\left( {{{\xi }_{i}}} \right)&=-H\left( \Delta \left( {{{\mathscr {F}}}_{N}} \right) \right) {{\mu }_{0}}{{{\xi }_{i}}}-J{{{\xi }_{i}}}\sum \limits _{E\left( i,k \right) \in S}{{{{\xi }_{k}}}} \\&=-{{{\xi }_{i}}}\left( H\left( \Delta \left( {{{\mathscr {F}}}_{N}} \right) \right) {{\mu }_{0}}+JK{\tilde{\xi }}\left( {{{\mathscr {F}}}_{N}} \right) \right) . \end{aligned}$$

From the Hamiltonian $H\left( {{\mathscr {F}}}_{N} \right)$ in (36), the $E\left( {{\mathscr {F}}}_{N} \right)$ energy of the system ${{\mathscr {S}}}\left( N\right)$ can be straightforwardly evaluated as

40 $$\begin{aligned} E\left( {{\mathscr {F}}}_{N} \right) =-\left| V\right| \left( H\left( \Delta \left( {{\mathscr {F}}}_{N} \right) \right) \mu _{0} {\tilde{\xi }}\left( {{\mathscr {F}}}_{N} \right) +{ \frac{1}{2}} {\tilde{K}}J\left( {\tilde{\xi }}\left( {{\mathscr {F}}}_{N} \right) \right) ^{2} \right) , \end{aligned}$$

while the $S_{e} \left( {{\mathscr {F}}}_{N} \right)$ entropy of ${{\mathscr {S}}}\left( N\right)$ is as (see also the Shannon–Boltzmann formula^125–127^)

41 $$\begin{aligned} S_{e} \left( {{\mathscr {F}}}_{N} \right) =-\left| V\right| c_{B} \sum _{i}f\left( {{\xi }_{i}}\right) \ln f\left( {{\xi }_{i}}\right) , \end{aligned}$$

where $c_{B}$ is a constant (set as the Boltzmann’s constant in statistical physics), while $f\left( {{\xi }_{i}}\right)$ is a distribution function (Gibbs state^125–127^) as

42 $$\begin{aligned} f\left( {{\xi }_{i}}\right) =\exp \left( { \frac{-H\left( {{\xi }_{i}}\right) }{c_{B} \varphi }} \right) , \end{aligned}$$

where $\varphi$ is the temperature in statistical physics^125–127^, however in our setting, $\varphi$ is an internal parameter called fluctuation frequency. The value of $\varphi$ quantifies the fluctuations of the network structure, and determined as follows.

Using $H\left( {{\xi }_{i}}\right)$ (see (39)) in (42), allows us to rewrite $f\left( {{\xi }_{i}}\right)$ in function of $f \left( B_{F} \left( {{\mathscr {F}}}_{N} \right) \right)$, $\Delta \left( {{\mathscr {F}}}_{N} \right)$ and ${\tilde{\xi }}\left( {{\mathscr {F}}}_{N} \right)$, as

43 $$\begin{aligned} f\left( {{\xi }_{i}}\right) ={ \frac{1}{\mathrm{X} }} \exp \left( \Delta \left( {{\mathscr {F}}}_{N} \right) +f \left( B_{F} \left( {{\mathscr {F}}}_{N} \right) \right) {\tilde{\xi }}\left( {{\mathscr {F}}}_{N} \right) \right) {{\xi }_{i}}, \end{aligned}$$

where $\mathrm{X}$ is a normalization term, defined as

44 $$\begin{aligned} {\mathrm{X}} =\sum _{i=1}^{\left| V\right| }\exp \left( \Delta \left( {{\cal{F}}}_{N} \right) +f \left( B_{F} \left( {{\cal{F}}}_{N} \right) \right) {\tilde{\xi }}\left( {{\cal{F}}}_{N} \right) \right) {{\xi }_{i}} , \end{aligned}$$

thus from (42) and (43), $\varphi$ is yielded as

45 $$\begin{aligned} \varphi ={ \frac{-H\left( {{\xi }_{i}}\right) }{c_{B} \left( \ln { \frac{1}{\mathrm{X} }} \left( \Delta \left( {{\mathscr {F}}}_{N} \right) +f \left( B_{F} \left( {{\mathscr {F}}}_{N} \right) \right) {\tilde{\xi }}\left( {{\mathscr {F}}}_{N} \right) \right) {{\xi }_{i}}\right) }} . \end{aligned}$$

Since, from some fundamentals of statistical physics^125^, the $\Psi _{{{\mathscr {F}}}_{N} }$ stability of ${{\mathscr {S}}}\left( N\right)$ is analogous to the difference of the $E\left( {{\mathscr {F}}}_{N} \right)$ energy and the weighted entropy $\varphi S_{e} \left( {{\mathscr {F}}}_{N} \right)$,

46 $$\begin{aligned} \Psi _{{{\mathscr {F}}}_{N} } =E\left( {{\mathscr {F}}}_{N} \right) -\varphi S_{e} \left( {{\mathscr {F}}}_{N} \right) , \end{aligned}$$

where $\varphi$ is the fluctuation frequency (analogous to temperature in the thermodynamical free energy potential function). The $\phi \left( F\left( {{\mathscr {F}}}_{N} \right) \right)$ term (34) therefore identifies the weighted average fidelity of ${{\mathscr {F}}}_{N}$, such that ${\tilde{\xi }}\left( {{\mathscr {F}}}_{N} \right)$ is a stochastic variable, since ${\tilde{\xi }}\left( {{\mathscr {F}}}_{N} \right)$ fluctuates over the system states ${{\mathscr {S}}}\left( N\left( t\right) \right)$, $t=1,\ldots ,T$, where *T* is a total system evaluation time period, and $N\left( t\right)$ is the state of *N* at a particular *t*. Therefore, at a particular system state ${{\mathscr {S}}}\left( N\left( t\right) \right)$, ${\tilde{\xi }}\left( {{\mathscr {F}}}_{N} \right)$ can be characterized by a function

47 $$\begin{aligned} \psi \left( {\tilde{\xi }}\left( {{\mathscr {F}}}_{N} \right) ,{{\mathscr {S}}}\left( N\left( t\right) \right) \right) =f\left( {\tilde{\xi }}\left( {{\mathscr {F}}}_{N} \right) ,{{\mathscr {S}}}\left( N\left( t\right) \right) \right) , \end{aligned}$$

such that $f\left( {\tilde{\xi }}\left( {{\mathscr {F}}}_{N} \right) ,{{\mathscr {S}}}\left( N\left( t\right) \right) \right)$ refers to (42) taken over ${\tilde{\xi }}\left( {{\mathscr {F}}}_{N} \right)$ at a given ${{\mathscr {S}}}\left( N\left( t\right) \right)$. The derivative of $\psi \left( {\tilde{\xi }}\left( {{\mathscr {F}}}_{N} \right) ,{{\mathscr {S}}}\left( N\left( t\right) \right) \right) =f\left( {\tilde{\xi }}\left( {{\mathscr {F}}}_{N} \right) ,{{\mathscr {S}}}\left( N\left( t\right) \right) \right)$ is evaluated as

48 $$\begin{aligned}&\tfrac{d\psi \left( {\tilde{\xi }}\left( {{{\mathscr {F}}}_{N}} \right) ,{\mathscr {S}}\left( N\left( t \right) \right) \right) }{d{\mathscr {S}}\left( N\left( t \right) \right) } \\&\quad = \sum \limits _{{{\tilde{\xi }}}'\left( {{{\mathscr {F}}}_{N}} \right) }{\Pr \left( \left. {\tilde{\xi }}\left( {{{\mathscr {F}}}_{N}} \right) \right| {{\tilde{\xi }}}'\left( {{{\mathscr {F}}}_{N}} \right) \right) \psi \left( {{\tilde{\xi }}}'\left( {{{\mathscr {F}}}_{N}} \right) ,{\mathscr {S}}\left( N\left( t \right) \right) \right) } \\&\qquad -\psi \left( {\tilde{\xi }}\left( {{{\mathscr {F}}}_{N}} \right) ,{\mathscr {S}}\left( N\left( t \right) \right) \right) \sum \limits _{{{\tilde{\xi }}}'\left( {{{\mathscr {F}}}_{N}} \right) }{\Pr \left( \left. {{\tilde{\xi }}}'\left( {{{\mathscr {F}}}_{N}} \right) \right| {\tilde{\xi }}\left( {{{\mathscr {F}}}_{N}} \right) \right) ,} \end{aligned}$$

where $\Pr \left( \left. {\tilde{\xi }}\left( {{\mathscr {F}}}_{N} \right) \right| {\tilde{\xi }}'\left( {{\mathscr {F}}}_{N} \right) \right)$ is the probability of the transition ${\tilde{\xi }}'\left( {{\mathscr {F}}}_{N} \right) \rightarrow {\tilde{\xi }}\left( {{\mathscr {F}}}_{N} \right)$ at a given state ${{\mathscr {S}}}\left( N\left( t\right) \right)$, $\Pr \left( \left. {\tilde{\xi }}'\left( {{\mathscr {F}}}_{N} \right) \right| {\tilde{\xi }}\left( {{\mathscr {F}}}_{N} \right) \right)$ is the probability of ${\tilde{\xi }}\left( {{\mathscr {F}}}_{N} \right) \rightarrow {\tilde{\xi }}'\left( {{\mathscr {F}}}_{N} \right)$ at a given ${{\mathscr {S}}}\left( N\left( t\right) \right)$. In statistical physics, (48) identifies the so-called master equation, or Chapmann–Kolmogorov equation^125,126^.

A challenge in the evaluation of (48) is the determination of the conditional probabilities for a given ${{\mathscr {S}}}\left( N\left( t\right) \right)$, and to find the solutions of the derivative ${ \frac{d\psi \left( {\tilde{\xi }}\left( {{\mathscr {F}}}_{N} \right) ,{{\mathscr {S}}}\left( N\left( t\right) \right) \right) }{d{{\mathscr {S}}}\left( N\left( t\right) \right) }} =0$ to determine the probability distribution of the state-transition function $\psi \left( {\tilde{\xi }}\left( {{\mathscr {F}}}_{N} \right) ,{{\mathscr {S}}}\left( N\left( t\right) \right) \right)$ .

The conditional probabilities in (48) are derived as follows. Assuming that ${{\mathscr {S}}}^{*} \left( N\right)$ is a current system state, the following condition can be written for the conditional probabilities:

49 $$\begin{aligned} \Pr \left( \left. {{\xi }_{i}}\right| {{\xi }'_{i}} \right) \psi \left( {{\xi }'_{i}} ,{{\mathscr {S}}}\left( N\left( t\right) \right) \right) =\Pr \left( \left. {{\xi }'_{i}} \right| {{\xi }_{i}}\right) \psi \left( {{\xi }_{i}},{{\mathscr {S}}}\left( N\left( t\right) \right) \right) . \end{aligned}$$

Then, using (42) with the Hamiltonian, the $\Phi \left( {{\xi }_{i}},{\tilde{\xi }}\left( {{\mathscr {F}}}_{N} \right) \right)$ distribution function at a particular ${\tilde{\xi }}\left( {{\mathscr {F}}}_{N} \right)$ can be evaluated, as

50 $$\begin{aligned} \Phi \left( {{\xi }_{i}},{\tilde{\xi }}\left( {{\mathscr {F}}}_{N} \right) \right) ={ \frac{1}{\omega }} \exp \left( \Delta \left( {{\mathscr {F}}}_{N} \right) +f \left( B_{F} \left( {{\mathscr {F}}}_{N} \right) \right) {\tilde{\xi }}\left( {{\mathscr {F}}}_{N} \right) \right) {{\xi }_{i}}, \end{aligned}$$

where $\omega$ is a normalization term,

51 $$\begin{aligned} \omega =\sum _{i}\exp \left( \Delta \left( {{\mathscr {F}}}_{N} \right) +f \left( B_{F} \left( {{\mathscr {F}}}_{N} \right) \right) {\tilde{\xi }}\left( {{\mathscr {F}}}_{N} \right) \right) {{\xi }_{i}} . \end{aligned}$$

Using (50) and (51), ${\tilde{\xi }}\left( {{\mathscr {F}}}_{N} \right)$ can be yielded as

52 $$\begin{aligned} {\tilde{\xi }}\left( {{{\mathscr {F}}}_{N}} \right)&=\sum \limits _{i}{{{{\xi }_{i}}}\Phi \left( {{{\xi }_{i}}},{\tilde{\xi }}\left( {{{\mathscr {F}}}_{N}} \right) \right) } \\&=\tfrac{1}{\omega }\sum \limits _{i}{{{{\xi }_{i}}}\exp \left( \Delta \left( {{{\mathscr {F}}}_{N}} \right) +{{f}_{s}}\left( {{B}_{F}}\left( {{{\mathscr {F}}}_{N}} \right) \right) {\tilde{\xi }}\left( {{{\mathscr {F}}}_{N}} \right) \right) {{{\xi }_{i}}}} \\&=\tfrac{\sum \nolimits _{i}{{{{\xi }_{i}}}\exp \left( \zeta \left( {{{\mathscr {F}}}_{N}} \right) {{{\xi }_{i}}} \right) }}{\sum \nolimits _{i}{\exp \left( \zeta \left( {{{\mathscr {F}}}_{N}} \right) {{{\xi }_{i}}} \right) }} \\&=\tfrac{\partial \Omega \left( {{{\mathscr {F}}}_{N}} \right) }{\partial \zeta \left( {{{\mathscr {F}}}_{N}} \right) }, \end{aligned}$$

where

53 $$\begin{aligned} \Omega \left( {{\mathscr {F}}}_{N} \right) =\ln \sum _{i}\exp \left( \zeta \left( {{\mathscr {F}}}_{N} \right) {{\xi }_{i}}\right) , \end{aligned}$$

and

54 $$\begin{aligned} \zeta \left( {{\mathscr {F}}}_{N} \right) =\Delta \left( {{\mathscr {F}}}_{N} \right) +f \left( B_{F} \left( {{\mathscr {F}}}_{N} \right) \right) {\tilde{\xi }}\left( {{\mathscr {F}}}_{N} \right) . \end{aligned}$$

Since, the value of ${{\xi }_{i}}$ can be selected from *W* possible values, the formula of (52) can be written as

55 $$\begin{aligned} {\tilde{\xi }}\left( {{\mathscr {F}}}_{N} \right) =s_{0} +\Delta s{ \frac{\exp \left( \zeta \left( {{\mathscr {F}}}_{N} \right) \Delta s\right) }{1-\left( \exp \left( \zeta \left( {{\mathscr {F}}}_{N} \right) \Delta s\right) \right) ^{W} }} \left( { \frac{1-\left( \exp \left( \zeta \left( {{\mathscr {F}}}_{N} \right) \Delta s\right) \right) ^{W} }{1-\left( \exp \left( \zeta \left( {{\mathscr {F}}}_{N} \right) \Delta s\right) \right) }} -W\left( \exp \left( \zeta \left( {{\mathscr {F}}}_{N} \right) \Delta s\right) \right) ^{W-1} \right) , \end{aligned}$$

where $$W=2$$, $$s_{0} =-1$$, and

56 $$\begin{aligned} \Delta s={ \frac{1}{i}} \left( {{\xi }_{i}}-s_{0} \right) =2. \end{aligned}$$

Therefore, for the entangled quantum network *N*, (55) can be written as

57 $$\begin{aligned} {\tilde{\xi }}\left( {{{\mathscr {F}}}_{N}} \right)&=\tfrac{\exp \left( \zeta \left( {{{\mathscr {F}}}_{N}} \right) \Delta s \right) -1}{\exp \left( \zeta \left( {{{\mathscr {F}}}_{N}} \right) \Delta s \right) +1} \\&=\tfrac{\exp \left( 2\zeta \left( {{{\mathscr {F}}}_{N}} \right) \right) -1}{\exp \left( 2\zeta \left( {{{\mathscr {F}}}_{N}} \right) \right) +1} \\&=\tfrac{\exp \left( \zeta \left( {{{\mathscr {F}}}_{N}} \right) \right) -\exp \left( -\zeta \left( {{{\mathscr {F}}}_{N}} \right) \right) }{\exp \left( \zeta \left( {{{\mathscr {F}}}_{N}} \right) \right) +\exp \left( -\zeta \left( {{{\mathscr {F}}}_{N}} \right) \right) } \\&=\tanh \left( \zeta \left( {{{\mathscr {F}}}_{N}} \right) \right) \\&=\tanh \left( \Delta \left( {{{\mathscr {F}}}_{N}} \right) +{{f}_{s}}\left( {{B}_{F}}\left( {{{\mathscr {F}}}_{N}} \right) \right) {\tilde{\xi }}\left( {{{\mathscr {F}}}_{N}} \right) \right) . \end{aligned}$$

Then, using (50), the formula of (49) can be rewritten as

58 $$\begin{aligned} \Pr \left( \left. {{\xi }_{i}}\right| {{\xi }'_{i}} \right) \Phi \left( {{\xi }'_{i}} ,{\tilde{\xi }}\left( {{\mathscr {F}}}_{N} \right) \right) =\Pr \left( \left. {{\xi }'_{i}} \right| {{\xi }_{i}}\right) \Phi \left( {{\xi }_{i}},{\tilde{\xi }}\left( {{\mathscr {F}}}_{N} \right) \right) , \end{aligned}$$

and since ${{\xi }_{i}}=\pm 1,{{\xi }'_{i}} =\mp 1$, the conditional probabilities are yielded as

59 $$\begin{aligned} \Pr \left( \left. {{\xi }_{i}}\right| {{\xi }'_{i}} \right) =Q\exp \left( -\left( \Delta \left( {{\mathscr {F}}}_{N} \right) +f \left( B_{F} \left( {{\mathscr {F}}}_{N} \right) \right) {\tilde{\xi }}\left( {{\mathscr {F}}}_{N} \right) \right) \right) \end{aligned}$$

and

60 $$\begin{aligned} \Pr \left( \left. {{\xi }'_{i}} \right| {{\xi }_{i}}\right) =Q\exp \left( \Delta \left( {{\mathscr {F}}}_{N} \right) +f \left( B_{F} \left( {{\mathscr {F}}}_{N} \right) \right) {\tilde{\xi }}\left( {{\mathscr {F}}}_{N} \right) \right) , \end{aligned}$$

where *Q* is a constant.

From (59) and (60), the derivative in (48) can be rewritten as

61 $$\begin{aligned}&\tfrac{d\psi \left( {\tilde{\xi }}\left( {{{\mathscr {F}}}_{N}} \right) ,{\mathscr {S}}\left( N\left( t \right) \right) \right) }{d{\mathscr {S}}\left( N\left( t \right) \right) } \\&\quad = -\left( {{\kappa }_{{{{\mathscr {F}}}_{N}}}}\left( {\tilde{\xi }}\left( {{{\mathscr {F}}}_{N}} \right) \right) \right) {{f}_{s}}\left( {{B}_{F}}\left( {{{\mathscr {F}}}_{N}} \right) \right) \psi \left( {\tilde{\xi }}\left( {{{\mathscr {F}}}_{N}} \right) ,{\mathscr {S}}\left( N\left( t \right) \right) \right) \\&\qquad +\left( {{\Pi }_{{{{\mathscr {F}}}_{N}}}}\left( {\tilde{\xi }}\left( {{{\mathscr {F}}}_{N}} \right) \right) \right) {{f}_{s}}\left( {{B}_{F}}\left( {{{\mathscr {F}}}_{N}} \right) \right) \tfrac{\partial \psi \left( {\tilde{\xi }}\left( {{{\mathscr {F}}}_{N}} \right) ,{\mathscr {S}}\left( N\left( t \right) \right) \right) }{\partial {\tilde{\xi }}\left( {{{\mathscr {F}}}_{N}} \right) }, \end{aligned}$$

where

62 $$\begin{aligned} \kappa _{{{\mathscr {F}}}_{N} } \left( {\tilde{\xi }}\left( {{\mathscr {F}}}_{N} \right) \right) =Q\left( \alpha -{\tilde{\xi }}\left( {{\mathscr {F}}}_{N} \right) \beta \right) , \end{aligned}$$

where

63 $$\begin{aligned} \alpha =\sinh \left( \Delta \left( {{\mathscr {F}}}_{N} \right) +f \left( B_{F} \left( {{\mathscr {F}}}_{N} \right) \right) {\tilde{\xi }}\left( {{\mathscr {F}}}_{N} \right) \right) \end{aligned}$$

and

64 $$\begin{aligned} \beta =\cosh \left( \Delta \left( {{\mathscr {F}}}_{N} \right) +f \left( B_{F} \left( {{\mathscr {F}}}_{N} \right) \right) {\tilde{\xi }}\left( {{\mathscr {F}}}_{N} \right) \right) , \end{aligned}$$

while

65 $$\begin{aligned} \Pi _{{{\mathscr {F}}}_{N} } \left( {\tilde{\xi }}\left( {{\mathscr {F}}}_{N} \right) \right) ={ \frac{1}{\left| V\right| }} Q\left( \beta -{\tilde{\xi }}\left( {{\mathscr {F}}}_{N} \right) \alpha \right) , \end{aligned}$$

therefore the solution^125,126^ of the derivative ${ \frac{d\psi \left( {\tilde{\xi }}\left( {{\mathscr {F}}}_{N} \right) ,{{\mathscr {S}}}\left( N\left( t\right) \right) \right) }{d{{\mathscr {S}}}\left( N\left( t\right) \right) }} =0$ is yielded as

66 $$\begin{aligned} \psi \left( {\tilde{\xi }}\left( {{\mathscr {F}}}_{N} \right) ,{{\mathscr {S}}}\left( N\left( t\right) \right) \right) ={ \frac{c}{\Pi _{{{\mathscr {F}}}_{N} } \left( {\tilde{\xi }}\left( {{\mathscr {F}}}_{N} \right) \right) }} \exp \left( \int \limits _{-1}^{{\tilde{\xi }}\left( {{\mathscr {F}}}_{N} \right) }{ \frac{\kappa _{{{\mathscr {F}}}_{N} } \left( x\right) }{\Pi _{{{\mathscr {F}}}_{N} } \left( x\right) }} dx \right) , \end{aligned}$$

where *c* is a constant.

It also can verified, that for ${{\xi }_{i}}=\pm 1$, (50) picks up the value of

67 $$\begin{aligned} \Phi \left( {{\xi }_{i}},{\tilde{\xi }}\left( {{\mathscr {F}}}_{N} \right) \right) ={ \frac{1\pm {\tilde{\xi }}\left( {{\mathscr {F}}}_{N} \right) }{2}} , \end{aligned}$$

thus using (39), the $S_{e} \left( \phi \left( F\left( {{\mathscr {F}}}_{N} \right) \right) \right)$ entropy at a particular $\phi \left( F\left( {{\mathscr {F}}}_{N} \right) \right)$ is as

68 $$\begin{aligned}&{{S}_{e}}\left( {{\phi }_{s}}\left( F\left( {{{\mathscr {F}}}_{N}} \right) \right) \right) \\&\quad = -\left| V \right| {{c}_{B}}\sum \limits _{i}{\Phi \left( {{{\xi }_{i}}},{\tilde{\xi }}\left( {{{\mathscr {F}}}_{N}} \right) \right) \ln \Phi \left( {{{\xi }_{i}}},{\tilde{\xi }}\left( {{{\mathscr {F}}}_{N}} \right) \right) } \\&\quad =-\left| V \right| {{c}_{B}}\left( \tfrac{1}{2} \left( 1+\tfrac{{{\phi }_{s}}\left( F\left( {{{\mathscr {F}}}_{N}} \right) \right) }{F\left( {{{\mathscr {F}}}_{N}} \right) } \right) \ln \left( 1+\tfrac{{{\phi }_{s}}\left( F\left( {{{\mathscr {F}}}_{N}} \right) \right) }{F\left( {{{\mathscr {F}}}_{N}} \right) } \right)\right. \\&\qquad +\tfrac{1}{2}\left( 1-\tfrac{{{\phi }_{s}}\left( F\left( {{{\mathscr {F}}}_{N}} \right) \right) }{F\left( {{{\mathscr {F}}}_{N}} \right) } \right) \left. \ln \left( 1-\tfrac{{{\phi }_{s}}\left( F\left( {{{\mathscr {F}}}_{N}} \right) \right) }{F\left( {{{\mathscr {F}}}_{N}} \right) } \right) \right) . \end{aligned}$$

As a corollary, from (46) and (68), the $\Psi _{{{\mathscr {F}}}_{N} } \left( \phi \left( F\left( {{\mathscr {F}}}_{N} \right) \right) \right)$ stability function at a particular $\phi \left( F\left( {{\mathscr {F}}}_{N} \right) \right)$ is yielded as

69 $$\begin{aligned} {{\Psi }_{{{{\mathscr {F}}}_{N}}}}\left( {{\phi }_{s}}\left( F\left( {{{\mathscr {F}}}_{N}} \right) \right) \right)&=E\left( {{{\mathscr {F}}}_{N}} \right) -\varphi {{S}_{e}}\left( {{\phi }_{s}}\left( F\left( {{{\mathscr {F}}}_{N}} \right) \right) \right) \\&=\left| V \right| {{c}_{B}}\varphi \left( \chi \left( {{{\mathscr {F}}}_{N}} \right) \right) , \end{aligned}$$

where $\chi \left( {{\mathscr {F}}}_{N} \right)$ is defined as

70 $$\begin{aligned} \chi \left( {{{\mathscr {F}}}_{N}} \right) &= -\Delta \left( {{{\mathscr {F}}}_{N}} \right) \tfrac{{{\phi }_{s}}\left( F\left( {{{\mathscr {F}}}_{N}} \right) \right) }{F\left( {{{\mathscr {F}}}_{N}} \right) }-\tfrac{1}{2}{{f}_{s}}\left( {{B}_{F}}\left( {{{\mathscr {F}}}_{N}} \right) \right) {{\left( \tfrac{{{\phi }_{s}}\left( F\left( {{{\mathscr {F}}}_{N}} \right) \right) }{F\left( {{{\mathscr {F}}}_{N}} \right) } \right) }^{2}} \\&\quad+\tfrac{1}{2}\left( 1+\tfrac{{{\phi }_{s}}\left( F\left( {{{\mathscr {F}}}_{N}} \right) \right) }{F\left( {{{\mathscr {F}}}_{N}} \right) } \right) \ln \left( 1+\tfrac{{{\phi }_{s}}\left( F\left( {{{\mathscr {F}}}_{N}} \right) \right) }{F\left( {{{\mathscr {F}}}_{N}} \right) } \right) \\&\quad +\tfrac{1}{2}\left( 1-\tfrac{{{\phi }_{s}}\left( F\left( {{{\mathscr {F}}}_{N}} \right) \right) }{F\left( {{{\mathscr {F}}}_{N}} \right) } \right) \ln \left( 1-\tfrac{{{\phi }_{s}}\left( F\left( {{{\mathscr {F}}}_{N}} \right) \right) }{F\left( {{{\mathscr {F}}}_{N}} \right) } \right) , \end{aligned}$$

where $\Delta \left( {{\mathscr {F}}}_{N} \right)$ is evaluated via (26) as

71 $$\begin{aligned} \Delta \left( {{{\mathscr {F}}}_{N}} \right)&=\tfrac{{{\mu }_{0}}H\left( \Delta \left( {{{\mathscr {F}}}_{N}} \right) \right) }{{{c}_{B}}\varphi } \\&=-\tfrac{\ln \left( f\left( {{{\xi }_{i}}} \right) \right) {{\mu }_{0}}H\left( \Delta \left( {{{\mathscr {F}}}_{N}} \right) \right) }{H\left( {{{\xi }_{i}}} \right) }, \end{aligned}$$

where $f\left( {{\xi }_{i}}\right)$ is given in (42), $H\left( {{\xi }_{i}}\right)$ is as in (39), while $f \left( B_{F} \left( {{\mathscr {F}}}_{N} \right) \right)$ can be rewritten via (37) as a statistical quantity

72 $$\begin{aligned} {{f}_{s}}\left( {{B}_{F}}\left( {{{\mathscr {F}}}_{N}} \right) \right)&=\tfrac{{\tilde{K}}J}{{{c}_{B}}\varphi } \\&=\tfrac{-\ln \left( f\left( {{{\xi }_{i}}} \right) \right) {\tilde{K}}J}{H\left( {{{\xi }_{i}}} \right) }. \end{aligned}$$

Therefore, such as $\phi \left( F\left( {{\mathscr {F}}}_{N} \right) \right)$ in (34), both $\Delta \left( {{\mathscr {F}}}_{N} \right)$ and $f \left( B_{F} \left( {{\mathscr {F}}}_{N} \right) \right)$ can be rewritten as statistical parameters of ${{\mathscr {S}}}\left( N\right)$ of the entangled quantum network *N*.

The next problem is the analysis of function (69) to derive the fluctuation model and the ${{\mathscr {S}}}^{*} \left( N\right)$ stable equilibrium state of the entangled network. The stability analysis of *N* is as follows.

To find the ${{\mathscr {S}}}^{*} \left( N\right)$ state of *N*, the derivative of (69) is taken, as

73 $$\begin{aligned} {{{{\Psi }'}}_{{{{\mathscr {F}}}_{N}}}}\left( {{\phi }_{s}}\left( F\left( {{{\mathscr {F}}}_{N}} \right) \right) \right)&=\tfrac{d{{\Psi }_{{{{\mathscr {F}}}_{N}}}}\left( {{\phi }_{s}}\left( F\left( {{{\mathscr {F}}}_{N}} \right) \right) \right) }{d{\tilde{\xi }}\left( {{{\mathscr {F}}}_{N}} \right) } \\&=-\left( \Delta \left( {{{\mathscr {F}}}_{N}} \right) +{{f}_{s}}\left( {{B}_{F}}\left( {{{\mathscr {F}}}_{N}} \right) \right) {\tilde{\xi }}\left( {{{\mathscr {F}}}_{N}} \right) \right) +\tfrac{1}{2}\ln \tfrac{1+{\tilde{\xi }}\left( {{{\mathscr {F}}}_{N}} \right) }{1-{\tilde{\xi }}\left( {{{\mathscr {F}}}_{N}} \right) }, \end{aligned}$$

from which the condition of $\Psi '_{{{\mathscr {F}}}_{N} } \left( \phi \left( F\left( {{\mathscr {F}}}_{N} \right) \right) \right) =0$ results in

74 $$\begin{aligned} \Delta \left( {{\mathscr {F}}}_{N} \right) +f \left( B_{F} \left( {{\mathscr {F}}}_{N} \right) \right) {\tilde{\xi }}\left( {{\mathscr {F}}}_{N} \right) ={ \frac{1}{2}} \ln { \frac{1+{\tilde{\xi }}\left( {{\mathscr {F}}}_{N} \right) }{1-{\tilde{\xi }}\left( {{\mathscr {F}}}_{N} \right) }} . \end{aligned}$$

Then, since

75 $$\begin{aligned} { \frac{1}{2}} \left( 1\pm {\tilde{\xi }}\left( {{\mathscr {F}}}_{N} \right) \right) ={ \frac{\exp \left( \pm \left( \Delta \left( {{\mathscr {F}}}_{N} \right) +f \left( B_{F} \left( {{\mathscr {F}}}_{N} \right) \right) {\tilde{\xi }}\left( {{\mathscr {F}}}_{N} \right) \right) \right) }{2\cosh \left( \Delta \left( {{\mathscr {F}}}_{N} \right) +f \left( B_{F} \left( {{\mathscr {F}}}_{N} \right) \right) {\tilde{\xi }}\left( {{\mathscr {F}}}_{N} \right) \right) }} , \end{aligned}$$

the result in (74) can be rewritten as

76 $$\begin{aligned} {\tilde{\xi }}\left( {{{\mathscr {F}}}_{N}} \right)&=\tfrac{1}{2}\left( 1+{\tilde{\xi }}\left( {{{\mathscr {F}}}_{N}} \right) \right) -\tfrac{1}{2}\left( 1-{\tilde{\xi }}\left( {{{\mathscr {F}}}_{N}} \right) \right) \\&=\tanh \left( \Delta \left( {{{\mathscr {F}}}_{N}} \right) +{{f}_{s}}\left( {{B}_{F}}\left( {{{\mathscr {F}}}_{N}} \right) \right) {\tilde{\xi }}\left( {{{\mathscr {F}}}_{N}} \right) \right) . \end{aligned}$$

As, the $\Delta \left( {{\mathscr {F}}}_{N} \right)$ average noise of the entanglement flow in the entangled structure is zero, $\Delta \left( {{\mathscr {F}}}_{N} \right) =0$, (76) is yielded as

77 $$\begin{aligned} {\tilde{\xi }}\left( {{{\mathscr {F}}}_{N}} \right) &= \left( {{s}_{0}}+\Delta s\tfrac{W-1}{2} \right) +\tfrac{{{W}^{2}}-1}{12}{{\left( \Delta s \right) }^{2}}{{f}_{s}}\left( {{B}_{F}}\left( {{{\mathscr {F}}}_{N}} \right) \right) {\tilde{\xi }}\left( {{{\mathscr {F}}}_{N}} \right) \\&\quad-\tfrac{{{W}^{4}}-1}{720}{{\left( \Delta s \right) }^{4}}{{\left( {{f}_{s}}\left( {{B}_{F}}\left( {{{\mathscr {F}}}_{N}} \right) \right) \right) }^{3}}{{\left( {\tilde{\xi }}\left( {{{\mathscr {F}}}_{N}} \right) \right) }^{3}}, \end{aligned}$$

where $W=2$, $s_{0} =-1$, $\Delta s={ \frac{1}{i}} \left( {{\xi }_{i}}-s_{0} \right) =2$, thus (77) can be rewritten as

78 $$\begin{aligned} {\tilde{\xi }}\left( {{{\mathscr {F}}}_{N}} \right)&=\tanh \left( {{f}_{s}}\left( {{B}_{F}}\left( {{{\mathscr {F}}}_{N}} \right) \right) {\tilde{\xi }}\left( {{{\mathscr {F}}}_{N}} \right) \right) \\&={{f}_{s}}\left( {{B}_{F}}\left( {{{\mathscr {F}}}_{N}} \right) \right) {\tilde{\xi }}\left( {{{\mathscr {F}}}_{N}} \right) -\tfrac{1}{3}{{\left( {{f}_{s}}\left( {{B}_{F}}\left( {{{\mathscr {F}}}_{N}} \right) \right) \right) }^{3}}{{\left( {\tilde{\xi }}\left( {{{\mathscr {F}}}_{N}} \right) \right) }^{3}}, \end{aligned}$$

with solutions ${\tilde{\xi }}\left( {{\mathscr {F}}}_{N} \right) _{0,1,2}$, as^125^

79 $$\begin{aligned} {\tilde{\xi }}\left( {{\mathscr {F}}}_{N} \right) _{0} =0, \end{aligned}$$

and

80 $$\begin{aligned} {\tilde{\xi }}\left( {{\mathscr {F}}}_{N} \right) _{1,2} =\pm \sqrt{{ \frac{3\left( f \left( B_{F} \left( {{\mathscr {F}}}_{N} \right) \right) -1\right) }{\left( f \left( B_{F} \left( {{\mathscr {F}}}_{N} \right) \right) \right) ^{3} }} } . \end{aligned}$$

As ${{\mathscr {S}}}^{*} \left( N\right)$ is determined via (79) and (80), the stability of ${{\mathscr {S}}}^{*} \left( N\right)$ can be determined via the second derivative of $\Psi _{{{\mathscr {F}}}_{N} } \left( \phi \left( F\left( {{\mathscr {F}}}_{N} \right) \right) \right)$, which is for a given solution $\tilde{{\xi }_{i}} \left( {{\mathscr {F}}}_{N} \right)$, $i=0,1,2$ is as

81 $$\begin{aligned} \Psi ''_{{{\mathscr {F}}}_{N} } \left( \phi \left( F\left( {{\mathscr {F}}}_{N} \right) \right) \right) =-f \left( B_{F} \left( {{\mathscr {F}}}_{N} \right) \right) +{ \frac{1}{1-\tilde{{\xi }_{i}} \left( {{\mathscr {F}}}_{N} \right) }} . \end{aligned}$$

From (81), the stability of the ${{\mathscr {S}}}^{*} \left( N\right)$ equilibrium state of the entangled quantum network *N* is as follows.

If

82 $$\begin{aligned} \Psi ''_{{{\mathscr {F}}}_{N} } \left( \phi \left( F\left( {{\mathscr {F}}}_{N} \right) \right) \right) <0, \end{aligned}$$

then the ${{\mathscr {S}}}^{*} \left( N\right)$ equilibrium state of the entangled quantum network *N* is stable (in a stable equilibrium state, system fluctuations cannot transform ${{\mathscr {S}}}^{*} \left( N\right)$ to a non-stable system state), if

83 $$\begin{aligned} \Psi ''_{{{\mathscr {F}}}_{N} } \left( \phi \left( F\left( {{\mathscr {F}}}_{N} \right) \right) \right) =0, \end{aligned}$$

then ${{\mathscr {S}}}^{*} \left( N\right)$ equilibrium state is critical stable (in a critical stable equilibrium state, the system is fragile and a small fluctuation can transform ${{\mathscr {S}}}^{*} \left( N\right)$ to a non-stable system state), while if

84 $$\begin{aligned} \Psi ''_{{{\mathscr {F}}}_{N} } \left( \phi \left( F\left( {{\mathscr {F}}}_{N} \right) \right) \right) >0, \end{aligned}$$

then the ${{\mathscr {S}}}^{*} \left( N\right)$ equilibrium state of the entangled quantum network *N* is non-stable.

As a corollary, if $\Delta \left( {{\mathscr {F}}}_{N} \right) =0$ and

85 $$\begin{aligned} B_{F} \left( {{\mathscr {F}}}_{N} \right) <B_{F}^{*} \left( {{\mathscr {F}}}_{N} \right) , \end{aligned}$$

then the ${{\mathscr {S}}}^{*} \left( N\right)$ equilibrium state of *N* is stable only for ${\tilde{\xi }}\left( {{\mathscr {F}}}_{N} \right) _{0} =0$. If $\Delta \left( {{\mathscr {F}}}_{N} \right) =0$ and

86 $$\begin{aligned} B_{F} \left( {{\mathscr {F}}}_{N} \right) \ge B_{F}^{*} \left( {{\mathscr {F}}}_{N} \right) , \end{aligned}$$

then the ${{\mathscr {S}}}^{*} \left( N\right)$ equilibrium state is stable only for ${\tilde{\xi }}\left( {{\mathscr {F}}}_{N} \right) _{1,2}$.

The stability derivation for the $\Delta \left( {{\mathscr {F}}}_{N} \right) >0$ case, is as follows.

From the series expansion of (69), $\Psi _{{{\mathscr {F}}}_{N} } \left( \phi \left( F\left( {{\mathscr {F}}}_{N} \right) \right) \right)$ can be rewritten as

87 $$\begin{aligned} {{\Psi }_{{{{\mathscr {F}}}_{N}}}}\left( {{\phi }_{s}}\left( F\left( {{{\mathscr {F}}}_{N}} \right) \right) \right) &= -\ln 2-\Delta \left( {{{\mathscr {F}}}_{N}} \right) {\tilde{\xi }}\left( {{{\mathscr {F}}}_{N}} \right) \\&\quad+\tfrac{1}{2}\left( 1-{{f}_{s}}\left( {{B}_{F}}\left( {{{\mathscr {F}}}_{N}} \right) \right) \right) {{\left( {\tilde{\xi }}\left( {{{\mathscr {F}}}_{N}} \right) \right) }^{2}}+\tfrac{1}{12}{{\left( {\tilde{\xi }}\left( {{{\mathscr {F}}}_{N}} \right) \right) }^{4}}, \end{aligned}$$

thus the ${{\mathscr {S}}}^{*} \left( N\right)$ equilibrium state can be determined from the $\Psi '_{{{\mathscr {F}}}_{N} } \left( \phi \left( F\left( {{\mathscr {F}}}_{N} \right) \right) \right)$ derivative of (87), as

88 $$\begin{aligned}&{{{{\Psi }'}}_{{{{\mathscr {F}}}_{N}}}}\left( {{\phi }_{s}}\left( F\left( {{{\mathscr {F}}}_{N}} \right) \right) \right) \\&\quad = -\Delta \left( {{{\mathscr {F}}}_{N}} \right) +\left( 1-{{f}_{s}}\left( {{B}_{F}}\left( {{{\mathscr {F}}}_{N}} \right) \right) \right) {\tilde{\xi }}\left( {{{\mathscr {F}}}_{N}} \right) +\tfrac{1}{3}{{\left( {\tilde{\xi }}\left( {{{\mathscr {F}}}_{N}} \right) \right) }^{3}}=0, \end{aligned}$$

with solutions^125,126^

89 $$\begin{aligned} \sum \limits _{i=0}^{2}{{\tilde{\xi }}{{\left( {{{\mathscr {F}}}_{N}} \right) }_{i}}}&=3\left( 1-{{f}_{s}}\left( {{B}_{F}}\left( {{{\mathscr {F}}}_{N}} \right) \right) \right) +{\tilde{\xi }}\left( {{{\mathscr {F}}}_{N}} \right) _{2}^{2}-\prod \limits _{i=0}^{1}{{\tilde{\xi }}{{\left( {{{\mathscr {F}}}_{N}} \right) }_{i}}} \\&=3\Delta \left( {{{\mathscr {F}}}_{N}} \right) -\prod \limits _{i=0}^{2}{{\tilde{\xi }}{{\left( {{{\mathscr {F}}}_{N}} \right) }_{i}}} \\&=0. \end{aligned}$$

The stability of the ${{\mathscr {S}}}^{*} \left( N\right)$ equilibrium state can be determined from the second derivate of (87),

90 $$\begin{aligned} \Psi ''_{{{\mathscr {F}}}_{N} } \left( \phi \left( F\left( {{\mathscr {F}}}_{N} \right) \right) \right) =\left( 1-f \left( B_{F} \left( {{\mathscr {F}}}_{N} \right) \right) \right) +\left( {\tilde{\xi }}\left( {{\mathscr {F}}}_{N} \right) \right) ^{2} . \end{aligned}$$

If ${{\mathscr {S}}}^{*} \left( N\right)$ is a critical stable equilibrium state, then

91 $$\begin{aligned} \Psi ''_{{{\mathscr {F}}}_{N} } \left( \phi \left( F\left( {{\mathscr {F}}}_{N} \right) \right) \right) =\left( 1-f \left( B_{F} \left( {{\mathscr {F}}}_{N} \right) \right) \right) +\left( x^{*} \right) ^{2} =0, \end{aligned}$$

which holds only if the parameters in (89) are set as

92 $$\begin{aligned} {\tilde{\xi }}\left( {{\mathscr {F}}}_{N} \right) _{0,1} =x^{*} , \end{aligned}$$

and

93 $$\begin{aligned} {\tilde{\xi }}\left( {{\mathscr {F}}}_{N} \right) _{2} =-2x^{*} , \end{aligned}$$

where

94 $$\begin{aligned} x^{*} =\pm \sqrt{\left( f \left( B_{F} \left( {{\mathscr {F}}}_{N} \right) \right) -1\right) } , \end{aligned}$$

with

95 $$\begin{aligned} f \left( B_{F} \left( {{\mathscr {F}}}_{N} \right) \right) >1 \end{aligned}$$

for a critical stable equilibrium state (91).

Therefore, if $f \left( B_{F} \left( {{\mathscr {F}}}_{N} \right) \right) \le 1$, then for any $\Delta \left( {{\mathscr {F}}}_{N} \right) >0$, $\Psi ''_{{{\mathscr {F}}}_{N} } \left( \phi \left( F\left( {{\mathscr {F}}}_{N} \right) \right) \right) \ne 0$ in (90). However, the entangled network structure still could have stable ${{\mathscr {S}}}^{*} \left( N\right)$ equilibrium state at $f \left( B_{F} \left( {{\mathscr {F}}}_{N} \right) \right) \le 1$ and $\Delta \left( {{\mathscr {F}}}_{N} \right) >0$, but not a critical stable.

At (91), the $\Delta ^{*} \left( {{\mathscr {F}}}_{N} \right)$ average noise for a critical stable equilibrium state (see (91)) is as

96 $$\begin{aligned} \Delta ^{*} \left( {{\mathscr {F}}}_{N} \right) =-{ \frac{2}{3}} \left( x^{*} \right) ^{3} =\mp { \frac{2}{3}} \sqrt{\left( f \left( B_{F} \left( {{\mathscr {F}}}_{N} \right) \right) -1\right) ^{3} } , \end{aligned}$$

such that

97 $$\begin{aligned} \left( \Delta ^{*} \left( {{\mathscr {F}}}_{N} \right) \right) ^{2} ={ \frac{4}{9}} \left( f \left( B_{F} \left( {{\mathscr {F}}}_{N} \right) \right) -1\right) ^{3} \end{aligned}$$

from which $f \left( B_{F} \left( {{\mathscr {F}}}_{N} \right) \right)$ can be rewritten as

98 $$\begin{aligned} f \left( B_{F} \left( {{\mathscr {F}}}_{N} \right) \right) =\root 3 \of {{ \frac{9}{4}} } \left( \Delta ^{*} \left( {{\mathscr {F}}}_{N} \right) \right) ^{\frac{2}{3} } +1. \end{aligned}$$

As follows, (95) can be rewritten as

99 $$\begin{aligned} \root 3 \of {{ \frac{9}{4}} } \left( \Delta ^{*} \left( {{\mathscr {F}}}_{N} \right) \right) ^{\frac{2}{3} } +1>1, \end{aligned}$$

which yields the condition for a critical stable state

100 $$\begin{aligned} \Delta ^{*} \left( {{\mathscr {F}}}_{N} \right) >0. \end{aligned}$$

The result in (100) indicates that for any $\Delta \left( {{\mathscr {F}}}_{N} \right) >0$ and $f \left( B_{F} \left( {{\mathscr {F}}}_{N} \right) \right) \ge 1$, the entangled network is in a ${\tilde{\xi }}\left( {{\mathscr {F}}}_{N} \right) =x^{*}$ critical stable equilibrium state ${{\mathscr {S}}}^{*} \left( N\right)$. If $\Delta \left( {{\mathscr {F}}}_{N} \right) >0$ and $f \left( B_{F} \left( {{\mathscr {F}}}_{N} \right) \right) <1$, the entangled network is in a stable or in a non-stable equilibrium state ${{\mathscr {S}}}^{*} \left( N\right)$.

To conclude the statements, if $\Delta \left( {{\mathscr {F}}}_{N} \right) >0$, then the stable ${{\mathscr {S}}}^{*} \left( N\right)$ equilibrium state is yielded at a system state ${\tilde{\xi }}\left( {{\mathscr {F}}}_{N} \right) _{i}$ that minimizes $\Psi _{{{\mathscr {F}}}_{N} } \left( \phi \left( F\left( {{\mathscr {F}}}_{N} \right) \right) \right)$, as a global minima

101 $$\begin{aligned} \Psi _{{{\mathscr {F}}}_{N} } \left( \phi \left( F\left( {{\mathscr {F}}}_{N} \right) \right) \right) =\min \left\{ \Psi _{{{\mathscr {F}}}_{N} } \left( F\left( {{\mathscr {F}}}_{N} \right) {\tilde{\xi }}\left( {{\mathscr {F}}}_{N} \right) _{i} \right) \right\} _{i=0}^{2} \end{aligned}$$

such that $${ \frac{1}{3}} \prod _{i=0}^{2}{\tilde{\xi }}\left( {{\mathscr {F}}}_{N} \right) _{i} =\Delta \left( {{\mathscr {F}}}_{N} \right) >0$$.

The derivations also reveals that for any $f \left( B_{F} \left( {{\mathscr {F}}}_{N} \right) \right) \ge 0$, the solutions of ${\tilde{\xi }}\left( {{\mathscr {F}}}_{N} \right)$ evaluated via (89) are determined by the actual value of $\Delta \left( {{\mathscr {F}}}_{N} \right)$. As a corollary, the minimal values of $\Psi _{{{\mathscr {F}}}_{N} } \left( \phi \left( F\left( {{\mathscr {F}}}_{N} \right) \right) \right)$ in (101) also depend on $\Delta \left( {{\mathscr {F}}}_{N} \right)$.

Then, let us assume that the entangled structure is in a ${{\mathscr {S}}}\left( N\left( t\right) \right) \ne {{\mathscr {S}}}^{*} \left( N\right)$ non-equilibrium state. Finally, it also can be verified that from ${{\mathscr {S}}}\left( N\left( t\right) \right)$ it is always possible to reach a stable ${{\mathscr {S}}}\left( N\left( t+\Gamma \right) \right) ={{\mathscr {S}}}^{*} \left( N\right)$ equilibrium state, as follows.

Let ${\tilde{\xi }}_{{{\mathscr {S}}}\left( N\left( t\right) \right) } \left( {{\mathscr {F}}}_{N} \right)$ and ${\tilde{\xi }}_{{{\mathscr {S}}}^{*} \left( N\right) } \left( {{\mathscr {F}}}_{N} \right)$ refer to (33) at ${{\mathscr {S}}}\left( N\left( t\right) \right)$ and ${{\mathscr {S}}}^{*} \left( N\right)$, defined as

102 $$\begin{aligned} {\tilde{\xi }}_{{{\mathscr {S}}}\left( N\left( t\right) \right) } \left( {{\mathscr {F}}}_{N} \right) ={\tilde{\xi }}_{{{\mathscr {S}}}^{*} \left( N\right) } \left( {{\mathscr {F}}}_{N} \right) +\lambda , \end{aligned}$$

where $\lambda$ after some calculations is yielded as

103 $$\begin{aligned} \lambda =A\sin \varsigma t+N\cos \varsigma t, \end{aligned}$$

where $\varsigma$ is as

104 $$\begin{aligned} \varsigma =\left( { \frac{\left( 1-f \left( B_{F} \left( {{\mathscr {F}}}_{N} \right) \right) +\left( {\tilde{\xi }}_{{{\mathscr {S}}}^{*} \left( N\right) } \left( {{\mathscr {F}}}_{N} \right) \right) ^{2} \right) }{G}} \right) ^{0.5} , \end{aligned}$$

where *G* is a constant, and

105 $$\begin{aligned} G{ \frac{d^{2} {\tilde{\xi }}_{{{\mathscr {S}}}\left( N\left( t\right) \right) } \left( {{\mathscr {F}}}_{N} \right) }{dt^{2} }} =-\left( 1-f \left( B_{F} \left( {{\mathscr {F}}}_{N} \right) \right) +\left( {\tilde{\xi }}_{{{\mathscr {S}}}^{*} \left( N\right) } \left( {{\mathscr {F}}}_{N} \right) \right) ^{2} \right) \lambda . \end{aligned}$$

Thus, from ${{\mathscr {S}}}\left( N\left( t\right) \right)$ the system will be in a stable ${{\mathscr {S}}}^{*} \left( N\right)$ at ${{\mathscr {S}}}\left( N\left( t+\Gamma \right) \right)$, as

106 $$\begin{aligned} {{\mathscr {S}}}\left( N\left( t+\Gamma \right) \right) ={{\mathscr {S}}}^{*} \left( N\right) , \end{aligned}$$

where

107 $$\begin{aligned} \Gamma ={ \frac{1}{\varsigma }} , \end{aligned}$$

where $\varsigma$ is given in (104).

The proof is concluded here. $\square$

#### Stable equilibrium state of the entangled network

This section illustrates the results of Theorem 1.

**Noiseless scenarios** Here, the stable equilibrium states of the entangled structure are determined for $\Delta \left( {{\mathscr {F}}}_{N} \right) =0$ scenarios.

**Figure 1:**
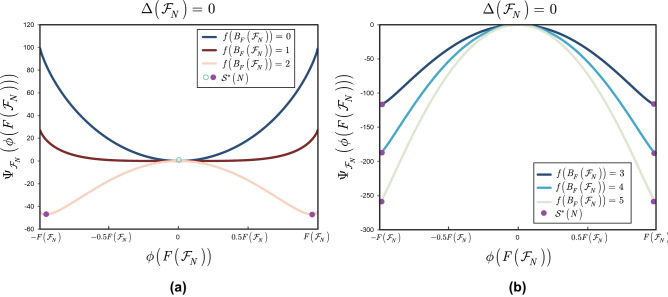
The stability function $\Psi _{{{\mathscr {F}}}_{N} } \left( \phi \left( F\left( {{\mathscr {F}}}_{N} \right) \right) \right)$ and the stable ${{\mathscr {S}}}^{*} \left( N\right)$ equilibrium states of the entangled quantum network *N* at $\Delta \left( {{\mathscr {F}}}_{N} \right) =0$, in function of $\phi \left( F\left( {{\mathscr {F}}}_{N} \right) \right) \in \left[ -F\left( {{\mathscr {F}}}_{N} \right) ,F\left( {{\mathscr {F}}}_{N} \right) \right]$ and $f \left( B_{F} \left( {{\mathscr {F}}}_{N} \right) \right)$. (**a**) The stable ${{\mathscr {S}}}^{*} \left( N\right)$ equilibrium state of the entangled structure for $f\left( B_{F} \left( {{\mathscr {F}}}_{N} \right) \right) =0,1$ (depicted by green-line empty dot), and the stable ${{\mathscr {S}}}^{*} \left( N\right)$ equilibrium states of the entangled structure for $f\left( B_{F} \left( {{\mathscr {F}}}_{N} \right) \right) =2$ (depicted by red dots). As $f \left( B_{F} \left( {{\mathscr {F}}}_{N} \right) \right) \le 1$, the entangled structure has one stable equilibrium state (green), while as $f\left( B_{F} \left( {{\mathscr {F}}}_{N} \right) \right) >1$, the entangled structure has two stable equilibrium states (red dots). (**b**) The stable ${{\mathscr {S}}}^{*} \left( N\right)$ equilibrium states of the entangled structure for $f\left( B_{F} \left( {{\mathscr {F}}}_{N} \right) \right) =3,4,5$ (depicted by red dots). Since $f\left( B_{F} \left( {{\mathscr {F}}}_{N} \right) \right) >1$, the entangled structure has two stable equilibrium states (red dots) for a given $f\left( B_{F} \left( {{\mathscr {F}}}_{N} \right) \right)$.

In Fig. 1, the stability function $\Psi _{{{\mathscr {F}}}_{N} } \left( \phi \left( F\left( {{\mathscr {F}}}_{N} \right) \right) \right)$ (see (69)) and the stable ${{\mathscr {S}}}^{*} \left( N\right)$ equilibrium states of the entangled quantum network *N* are depicted at average noise $\Delta \left( {{\mathscr {F}}}_{N} \right) =0$ (71), in function of the normalized fidelity $\phi \left( F\left( {{\mathscr {F}}}_{N} \right) \right) \in \left[ -F\left( {{\mathscr {F}}}_{N} \right) ,F\left( {{\mathscr {F}}}_{N} \right) \right]$ (see (22)) and normalized entanglement throughput $f \left( B_{F} \left( {{\mathscr {F}}}_{N} \right) \right)$ (see (23)). As it is depicted in Fig. 1a, if $f \left( B_{F} \left( {{\mathscr {F}}}_{N} \right) \right) \le 1$, then the entangled structure has only one stable equilibrium state at $\phi \left( F\left( {{\mathscr {F}}}_{N} \right) \right) =0$ (depicted by the green-line empty dot). As depicted in Fig. 1b, if $f\left( B_{F} \left( {{\mathscr {F}}}_{N} \right) \right) >1$, then entangled structure has two different stable equilibrium states (depicted by the red dots) at $\phi \left( F\left( {{\mathscr {F}}}_{N} \right) \right) =-F\left( {{\mathscr {F}}}_{N} \right)$ and $\phi \left( F\left( {{\mathscr {F}}}_{N} \right) \right) =F\left( {{\mathscr {F}}}_{N} \right)$.

**Noisy scenarios** Here, the stable equilibrium states of the entangled quantum network are determined for $\Delta \left( {{\mathscr {F}}}_{N} \right) >0$ scenarios.

**Figure 2:**
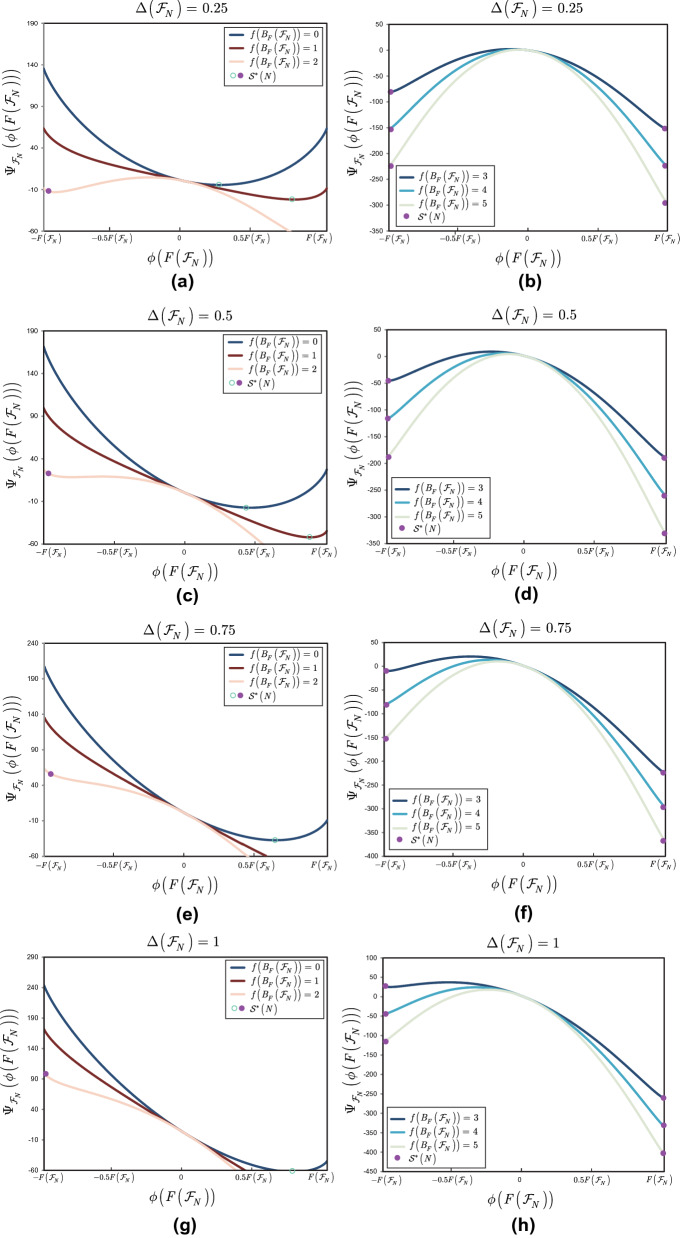
The stability function $\Psi _{{{\mathscr {F}}}_{N} } \left( \phi \left( F\left( {{\mathscr {F}}}_{N} \right) \right) \right)$ and the stable ${{\mathscr {S}}}^{*} \left( N\right)$ equilibrium states of the entangled quantum network *N* at $\Delta \left( {{\mathscr {F}}}_{N} \right) >0$, in function of $\phi \left( F\left( {{\mathscr {F}}}_{N} \right) \right) \in \left[ -F\left( {{\mathscr {F}}}_{N} \right) ,F\left( {{\mathscr {F}}}_{N} \right) \right]$ and $f \left( B_{F} \left( {{\mathscr {F}}}_{N} \right) \right)$, $\left| V\right| =100$. (**a**) The stable ${{\mathscr {S}}}^{*} \left( N\right)$ equilibrium state of the entangled structure at $\Delta \left( {{\mathscr {F}}}_{N} \right) =0.25$ and for $f\left( B_{F} \left( {{\mathscr {F}}}_{N} \right) \right) =0,1$ (depicted by green-line empty dot), and the stable ${{\mathscr {S}}}^{*} \left( N\right)$ equilibrium states of the entangled structure for $f\left( B_{F} \left( {{\mathscr {F}}}_{N} \right) \right) =2$ (depicted by red dots). (**b**) The stable ${{\mathscr {S}}}^{*} \left( N\right)$ equilibrium states of the entangled structure for $\Delta \left( {{\mathscr {F}}}_{N} \right) =0.25$ and $f\left( B_{F} \left( {{\mathscr {F}}}_{N} \right) \right) =3,4,5$ (depicted by red dots). (**c**) The stable ${{\mathscr {S}}}^{*} \left( N\right)$ equilibrium state of the entangled structure at $\Delta \left( {{\mathscr {F}}}_{N} \right) =0.5$ and for $f\left( B_{F} \left( {{\mathscr {F}}}_{N} \right) \right) =0,1$ (depicted by green-line empty dot), and the stable ${{\mathscr {S}}}^{*} \left( N\right)$ equilibrium states of the entangled structure for $f\left( B_{F} \left( {{\mathscr {F}}}_{N} \right) \right) =2$ (depicted by red dots). (**d**) The stable ${{\mathscr {S}}}^{*} \left( N\right)$ equilibrium states of the entangled structure for $\Delta \left( {{\mathscr {F}}}_{N} \right) =0.5$ and $f\left( B_{F} \left( {{\mathscr {F}}}_{N} \right) \right) =3,4,5$ (depicted by red dots). (**e**) The stable ${{\mathscr {S}}}^{*} \left( N\right)$ equilibrium state of the entangled structure at $\Delta \left( {{\mathscr {F}}}_{N} \right) =0.75$ and for $f\left( B_{F} \left( {{\mathscr {F}}}_{N} \right) \right) =0,1$ (depicted by green-line empty dot), and the stable ${{\mathscr {S}}}^{*} \left( N\right)$ equilibrium states of the entangled structure for $f\left( B_{F} \left( {{\mathscr {F}}}_{N} \right) \right) =2$ (depicted by red dots). (**f**) The stable ${{\mathscr {S}}}^{*} \left( N\right)$ equilibrium states of the entangled structure for $\Delta \left( {{\mathscr {F}}}_{N} \right) =0.75$ and $f\left( B_{F} \left( {{\mathscr {F}}}_{N} \right) \right) =3,4,5$ (depicted by red dots). (**g**) The stable ${{\mathscr {S}}}^{*} \left( N\right)$ equilibrium state of the entangled structure at $\Delta \left( {{\mathscr {F}}}_{N} \right) =1$ and for $f\left( B_{F} \left( {{\mathscr {F}}}_{N} \right) \right) =0,1$ (depicted by green-line empty dot), and the stable ${{\mathscr {S}}}^{*} \left( N\right)$ equilibrium states of the entangled structure for $f\left( B_{F} \left( {{\mathscr {F}}}_{N} \right) \right) =2$ (depicted by red dots). (**h**) The stable ${{\mathscr {S}}}^{*} \left( N\right)$ equilibrium states of the entangled structure for $\Delta \left( {{\mathscr {F}}}_{N} \right) =1$ and $f\left( B_{F} \left( {{\mathscr {F}}}_{N} \right) \right) =3,4,5$ (depicted by red dots).

In Fig. 2, the stability function $\Psi _{{{\mathscr {F}}}_{N} } \left( \phi \left( F\left( {{\mathscr {F}}}_{N} \right) \right) \right)$ (see (69)) and the stable ${{\mathscr {S}}}^{*} \left( N\right)$ equilibrium states of the entangled quantum network *N* are depicted at average noise $\Delta \left( {{\mathscr {F}}}_{N} \right) >0$ (71), in function of the normalized fidelity $\phi \left( F\left( {{\mathscr {F}}}_{N} \right) \right) \in \left[ -F\left( {{\mathscr {F}}}_{N} \right) ,F\left( {{\mathscr {F}}}_{N} \right) \right]$ (see (22)) and normalized entanglement throughput $f \left( B_{F} \left( {{\mathscr {F}}}_{N} \right) \right)$ (see (23)). As it is depicted in Fig. 2a, c, e, g, if $\Delta \left( {{\mathscr {F}}}_{N} \right) >0$ and $f \left( B_{F} \left( {{\mathscr {F}}}_{N} \right) \right) \le 1$, then the entangled structure has only one stable equilibrium state at a particular $\phi \left( F\left( {{\mathscr {F}}}_{N} \right) \right) >0$ (depicted by the green-line empty dot). As depicted in Fig. 2b, d, f, h, if $\Delta \left( {{\mathscr {F}}}_{N} \right) >0$ and $f\left( B_{F} \left( {{\mathscr {F}}}_{N} \right) \right) >1$, then entangled structure has two different stable equilibrium states (depicted by the red dots) at $\phi \left( F\left( {{\mathscr {F}}}_{N} \right) \right) =-F\left( {{\mathscr {F}}}_{N} \right)$ and $\phi \left( F\left( {{\mathscr {F}}}_{N} \right) \right) =F\left( {{\mathscr {F}}}_{N} \right)$.

A comparative analysis is included in Section A.1 of the Supplementary Information.

### Weakly and strongly entangled structures of the quantum Internet

#### Theorem 2

(Weakly and strongly entangled subnetworks of the entangled network) *For a weakly entangled subnetwork* ${{\mathscr {S}}}'_{N}$, *the* $F\left( {{\mathscr {S}}}'_{N} \right) =\left\{ F_{{{\mathscr {P}}}} \left( R_{i} \right) \right\} _{i=1}^{\left| {{\mathscr {S}}}'_{N} \right| }$ *fidelities of the nodes of* ${{\mathscr {S}}}'_{N}$ *are uncorrelated, while for a strongly entangled subnetwork* ${{\mathscr {S}}}_{N}^{*}$, *the* $F\left( {{\mathscr {S}}}_{N}^{*} \right) =\left\{ F_{{{\mathscr {P}}}} \left( R_{i} \right) \right\} _{i=1}^{\left| {{\mathscr {S}}}_{N}^{*} \right| }$ *fidelities of the nodes in* ${{\mathscr {S}}}_{N}^{*}$ *are correlated.*

#### Proof

Let ${{\mathscr {S}}}_{N}$ refer to a subnetwork of *N* with $\left| {{\mathscr {S}}}_{N} \right|$ quantum nodes. For the definition of ${{\mathscr {S}}}'_{N}$ weakly entangled and ${{\mathscr {S}}}_{N}^{*}$ strongly entangled subnetworks, see (17) and (19), respectively. For any $\Delta \left( {{\mathscr {F}}}_{N} \right)$, *N* has strongly entangled subnetworks, ${{\mathscr {S}}}_{N}$, only if $f\left( B_{F} \left( {{\mathscr {F}}}_{N} \right) \right) \ge 1$, while for $f\left( B_{F} \left( {{\mathscr {F}}}_{N} \right) \right) <1$, *N* has only weakly entangled subnetworks.

For the $\left| {{\mathscr {S}}}_{N} \right|$ nodes of ${{\mathscr {S}}}_{N}$, $R_{i}$, $i=1,\ldots ,\left| {{\mathscr {S}}}_{N} \right|$, let $\Delta F\left( {{\mathscr {S}}}_{N} \right)$ be a fidelity measure, defined as

108 $$\begin{aligned} \Delta F\left( {{\mathscr {S}}}_{N} \right) =\sum _{i=1}^{\left| {{\mathscr {S}}}_{N} \right| }\Delta F_{{{\mathscr {P}}}} \left( R_{i} \right) , \end{aligned}$$

where $\Delta F_{{{\mathscr {P}}}} \left( R_{i} \right)$ is defined in (30), $R_{i} \in {{\mathscr {S}}}_{N}$.

Then, let $M\left( {{\mathscr {S}}}_{N} \right)$ be the number of ${{\mathscr {S}}}_{N}$ subnetworks, and let ${{\mathscr {S}}}_{N}^{\left( z\right) }$ refer to an *z*th subnetwork, $z=1,\ldots ,M\left( {{\mathscr {S}}}_{N} \right)$. For the $M\left( {{\mathscr {S}}}_{N} \right)$ subnetworks, let $\mu _{N} \left( {{\mathscr {S}}}_{N} ,\Delta \left( {{\mathscr {F}}}_{N} \right) \right)$ be the average of (108) , as

109 $$\begin{aligned} {{\mu }_{N}}\left( {{{\mathscr {S}}}_{N}},\Delta \left( {{{\mathscr {F}}}_{N}} \right) \right)&=\tfrac{1}{M\left( {{{\mathscr {S}}}_{N}} \right) }\sum \limits _{z=1}^{M\left( {{{\mathscr {S}}}_{N}} \right) }{\Delta F\left( {\mathscr {S}}_{N}^{\left( z \right) } \right) } \\&=\tfrac{1}{M\left( {{{\mathscr {S}}}_{N}} \right) }\sum \limits _{z=1}^{M\left( {{{\mathscr {S}}}_{N}} \right) }{\sum \limits _{i=1}^{\left| {\mathscr {S}}_{N}^{\left( z \right) } \right| }{\Delta {{F}_{{\mathscr {P}}}}\left( {{R}_{i}} \right) }}. \end{aligned}$$

From (108) and (109), the weakly and strongly entangled subnetworks can be determined for any $f\left( B_{F} \left( {{\mathscr {F}}}_{N} \right) \right)$ and $\Delta \left( {{\mathscr {F}}}_{N} \right)$.

As a corollary, at $\Delta \left( {{\mathscr {F}}}_{N} \right) =0$ for a weakly entangled subnetwork ${{\mathscr {S}}}'_{N}$, the

110 $$\begin{aligned} F\left( {{\mathscr {S}}}'_{N} \right) =\left\{ F_{{{\mathscr {P}}}} \left( R_{i} \right) \right\} _{i=1}^{\left| {{\mathscr {S}}}'_{N} \right| } \end{aligned}$$

fidelities of the nodes of ${{\mathscr {S}}}'_{N}$ are uncorrelated that leads to

111 $$\begin{aligned} \mu _{N} \left( {{\mathscr {S}}}'_{N} ,0\right) \approx 0, \end{aligned}$$

since $\Delta \left( {{\mathscr {F}}}_{N} \right) =0$, the value of $\Delta F\left( {{\mathscr {S}}}'_{N} \right)$ in (108) is as

112 $$\begin{aligned} \Delta F\left( {{\mathscr {S}}}'_{N} \right) \approx 0 \end{aligned}$$

for the nodes of ${{\mathscr {S}}}_{N}^{*}$.

While for a strongly entangled subnetwork ${{\mathscr {S}}}_{N}^{*}$, the

113 $$\begin{aligned} F\left( {{\mathscr {S}}}_{N}^{*} \right) =\left\{ F_{{{\mathscr {P}}}} \left( R_{i} \right) \right\} _{i=1}^{\left| {{\mathscr {S}}}_{N}^{*} \right| } \end{aligned}$$

fidelities of the nodes in ${{\mathscr {S}}}_{N}^{*}$ are correlated via an entanglement purification $P_{{{\mathscr {S}}}_{N} }$, that leads to

114 $$\begin{aligned} \mu _{N} \left( {{\mathscr {S}}}_{N}^{*} ,0\right) \ll 0. \end{aligned}$$

It is because at $\Delta \left( {{\mathscr {F}}}_{N} \right) =0$, the value of $\Delta F\left( {{\mathscr {S}}}_{N}^{*} \right)$ in (108) is as

115 $$\begin{aligned} \Delta F\left( {{\mathscr {S}}}_{N}^{*} \right) \le 0 \end{aligned}$$

for the nodes of ${{\mathscr {S}}}_{N}^{*}$.

As $\Delta \left( {{\mathscr {F}}}_{N} \right) >0$, both $\mu _{N} \left( {{\mathscr {S}}}'_{N} \right)$ and $\mu _{N} \left( {{\mathscr {S}}}_{N}^{*} \right)$ are increased,

116 $$\begin{aligned} \mu _{N} \left( {{\mathscr {S}}}'_{N} ,\Delta \left( {{\mathscr {F}}}_{N} \right)>0\right) >\mu _{N} \left( {{\mathscr {S}}}'_{N} ,0\right) , \end{aligned}$$

and

117 $$\begin{aligned} \mu _{N} \left( {{\mathscr {S}}}_{N}^{*} ,\Delta \left( {{\mathscr {F}}}_{N} \right)>0\right) >\mu _{N} \left( {{\mathscr {S}}}_{N}^{*} ,0\right) , \end{aligned}$$

since the values of $\Delta F\left( {{\mathscr {S}}}'_{N} \right)$ in (108) and $\Delta F\left( {{\mathscr {S}}}_{N}^{*} \right)$ in (115) are decreased due to $\Delta \left( {{\mathscr {F}}}_{N} \right) >0$.

The proof is concluded here. $\square$

#### Weakly and strongly entangled structures

In this section, the results are illustrated with entangled network structures.

**Figure 3:**
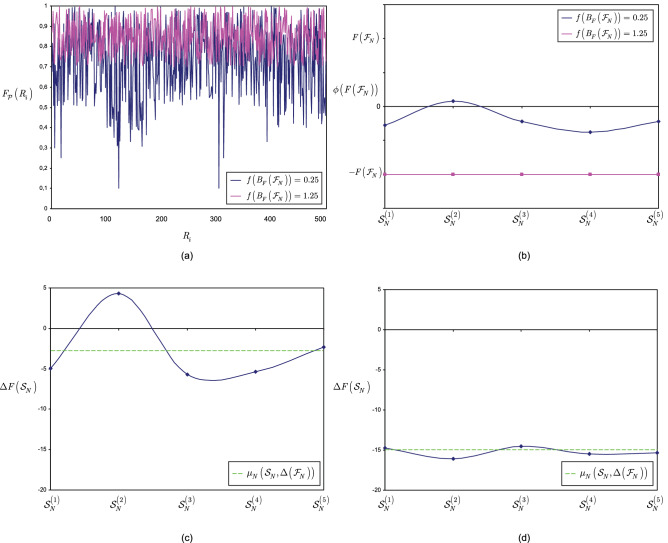
Weakly and strongly entangled structures at $\Delta \left( {{\mathscr {F}}}_{N} \right) =0$, $N=500$ and $M\left( {{\mathscr {S}}}_{N} \right) =5$, $\left| {{\mathscr {S}}}_{N}^{\left( z\right) } \right| =100$, $z=1,\ldots ,M\left( {{\mathscr {S}}}_{N} \right)$. (**a**) The distribution of the $F_{{{\mathscr {P}}}} \left( R_{i} \right)$ node fidelities at $F_{{{\mathscr {P}}}}^{*} \left( R_{i} \right) =0.7$, $i=1,\ldots ,N$, of a weakly entangled structure at $f\left( B_{F} \left( {{\mathscr {F}}}_{N} \right) \right) =0.25$ (depicted by black), and of a strongly entangled structure at $f\left( B_{F} \left( {{\mathscr {F}}}_{N} \right) \right) =1.25$ (depicted by red). (**b**) The $\phi \left( F\left( {{\mathscr {F}}}_{N} \right) \right)$ normalized fidelities of the $M\left( {{\mathscr {S}}}_{N} \right)$ subnetworks ${{\mathscr {S}}}_{N}^{\left( z\right) }$, $z=1,\ldots ,M\left( {{\mathscr {S}}}_{N} \right)$, for a weakly entangled structure at $f\left( B_{F} \left( {{\mathscr {F}}}_{N} \right) \right) =0.25$ (depicted by black), and of a strongly entangled structure at $f\left( B_{F} \left( {{\mathscr {F}}}_{N} \right) \right) =1.25$ (depicted by red). (**c**) The $\Delta F\left( {{\mathscr {S}}}_{N} \right)$ values of the $M\left( {{\mathscr {S}}}_{N} \right)$ subnetworks ${{\mathscr {S}}}_{N}^{\left( z\right) }$, $z=1,\ldots ,M\left( {{\mathscr {S}}}_{N} \right)$, for a weakly entangled structure at $f\left( B_{F} \left( {{\mathscr {F}}}_{N} \right) \right) =0.25$ (depicted by black) and the $\mu _{N} \left( {{\mathscr {S}}}_{N} ,\Delta \left( {{\mathscr {F}}}_{N} \right) \right) =\mu _{N} \left( {{\mathscr {S}}}'_{N} ,0\right) \approx 0$ average (depicted by dashed green line). (**d**) The $\Delta F\left( {{\mathscr {S}}}_{N} \right)$ values of the $M\left( {{\mathscr {S}}}_{N} \right)$ subnetworks ${{\mathscr {S}}}_{N}^{\left( z\right) }$, $z=1,\ldots ,M\left( {{\mathscr {S}}}_{N} \right)$, for a strongly entangled structure at $f\left( B_{F} \left( {{\mathscr {F}}}_{N} \right) \right) =1.25$ (depicted by black) and the $\mu _{N} \left( {{\mathscr {S}}}_{N} ,\Delta \left( {{\mathscr {F}}}_{N} \right) \right) =\mu _{N} \left( {{\mathscr {S}}}_{N}^{*} ,0\right) \ll 0$ average (depicted by dashed green line).

**Noiseless scenarios** The results are depicted in Fig. 3 for strongly and weakly entangled structures at $\Delta \left( {{\mathscr {F}}}_{N} \right) =0$, $N=500$ and $M\left( {{\mathscr {S}}}_{N} \right) =5$, $\left| {{\mathscr {S}}}_{N}^{\left( z\right) } \right| =100$, $z=1,\ldots ,M\left( {{\mathscr {S}}}_{N} \right)$.

The results of Fig. 3 are detailed as follows. At $\Delta \left( {{\mathscr {F}}}_{N} \right) =0$, the weakly and strongly entangled structures have a fundamentally different characteristics. While for a weakly entangled structure ${{\mathscr {S}}}'_{N}$ at $f\left( B_{F} \left( {{\mathscr {F}}}_{N} \right) \right) =0.25$, the $F_{{{\mathscr {P}}}} \left( R_{i} \right)$ fidelities of the nodes are uncorrelated and statistically independent, for a given subnetwork ${{\mathscr {S}}}_{N}^{\left( z\right) }$, the number of quantum repeaters with fidelity $F_{{{\mathscr {P}}}} \left( R_{i} \right) <F_{{{\mathscr {P}}}}^{*} \left( R_{i} \right)$ and $F_{{{\mathscr {P}}}} \left( R_{i} \right) \ge F_{{{\mathscr {P}}}}^{*} \left( R_{i} \right)$ are statistically equal. As a corollary, in Fig. 3b, the $\phi \left( F\left( {{\mathscr {F}}}_{N} \right) \right)$ normalized fidelities taken for the quantum repeaters of the subnetworks are around zero, $\phi \left( F\left( {{\mathscr {F}}}_{N} \right) \right) \approx 0$. On the other hand, for a strongly entangled structure ${{\mathscr {S}}}_{N}^{*}$ at $f\left( B_{F} \left( {{\mathscr {F}}}_{N} \right) \right) =1.25$, the distribution of the $F_{{{\mathscr {P}}}} \left( R_{i} \right)$ fidelities are fundamentally different, since the quantum repeaters are connected via high entanglement-throughput connections that allows to perform $P_{{{\mathscr {S}}}_{N} }$ entanglement purification between the nodes. As a corollary, if the quantum nodes are connected via high entanglement-throughput connections, the quantum nodes formulate a strongly entangled structure, and the $F_{{{\mathscr {P}}}} \left( R_{i} \right)$ fidelities become correlated. As follows, for a strongly entangled subnetwork ${{\mathscr {S}}}_{N}^{*}$, the fidelities of the quantum nodes are statistically not independent. Therefore, the $\phi \left( F\left( {{\mathscr {F}}}_{N} \right) \right)$ normalized fidelities taken for the quantum repeaters of the subnetworks are as $\phi \left( F\left( {{\mathscr {F}}}_{N} \right) \right) \approx -F\left( {{\mathscr {F}}}_{N} \right)$, since for the nodes of the strongly entangled subnetwork, the corresponding relation is $F_{{{\mathscr {P}}}} \left( R_{i} \right) \ge F_{{{\mathscr {P}}}}^{*} \left( R_{i} \right)$. Since the $\Delta F\left( {{\mathscr {S}}}_{N} \right)$ values are evaluated from the $F_{{{\mathscr {P}}}} \left( R_{i} \right)$ node fidelities at a particular $F_{{{\mathscr {P}}}}^{*} \left( R_{i} \right)$, the fundamentally different characteristics of the weakly and strongly entangled structure are also reflected in Fig. 3c, d. While for a weakly entangled structure ${{\mathscr {S}}}'_{N}$, the $\Delta F\left( {{\mathscr {S}}}_{N} \right)$ is around zero, for a strongly entangled structure ${{\mathscr {S}}}_{N}^{*}$, $\Delta F\left( {{\mathscr {S}}}_{N} \right)$ is significantly below zero. As a corollary, the $\mu _{N} \left( {{\mathscr {S}}}_{N} ,\Delta \left( {{\mathscr {F}}}_{N} \right) \right)$ average converges to zero for a weakly entangled structure ${{\mathscr {S}}}'_{N}$, while it is significantly below zero for a strongly entangled structure ${{\mathscr {S}}}_{N}^{*}$.

**Figure 4:**
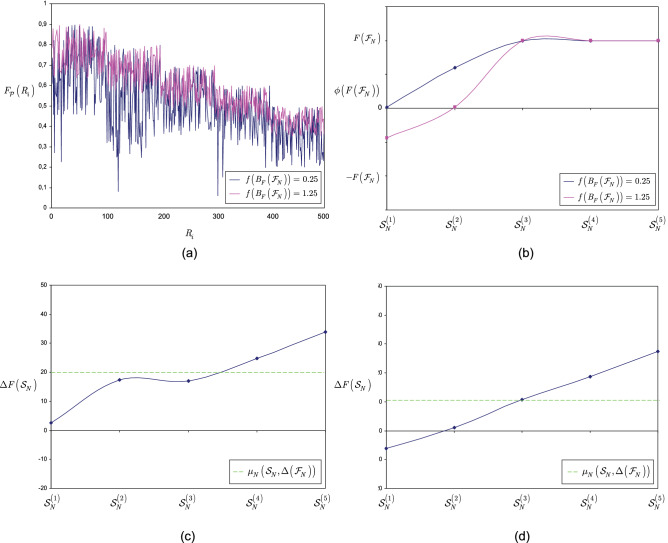
Weakly and strongly entangled structures at $\Delta \left( {{\mathscr {F}}}_{N} \right) >0$, $N=500$ and $M\left( {{\mathscr {S}}}_{N} \right) =5$, $\left| {{\mathscr {S}}}_{N}^{\left( z\right) } \right| =100$, $z=1,\ldots ,M\left( {{\mathscr {S}}}_{N} \right)$. The $\Delta \left( {{\mathscr {S}}}_{N}^{\left( z\right) } \right)$ subnetwork noise for a *z*th subnetwork ${{\mathscr {S}}}_{N}^{\left( z\right) }$ is set as $\Delta \left( {\mathscr {S}}_{N}^{\left( z \right) } \right) ={z}/{10}\;$. (**a**) The distribution of the $F_{{{\mathscr {P}}}} \left( R_{i} \right)$ node fidelities at $\Delta \left( {{\mathscr {F}}}_{N} \right) >0$, $F_{{{\mathscr {P}}}}^{*} \left( R_{i} \right) =0.7$, $i=1,\ldots ,100$, of a weakly entangled structure at $f\left( B_{F} \left( {{\mathscr {F}}}_{N} \right) \right) =0.25$ (depicted by black), and of a strongly entangled structure at $f\left( B_{F} \left( {{\mathscr {F}}}_{N} \right) \right) =1.25$ (depicted by red). (**b**) The $\phi \left( F\left( {{\mathscr {F}}}_{N} \right) \right)$ values of the $M\left( {{\mathscr {S}}}_{N} \right)$ subnetworks ${{\mathscr {S}}}_{N}^{\left( z\right) }$, $z=1,\ldots ,M\left( {{\mathscr {S}}}_{N} \right)$ at $\Delta \left( {{\mathscr {F}}}_{N} \right) >0$, for a weakly entangled structure at $f\left( B_{F} \left( {{\mathscr {F}}}_{N} \right) \right) =0.25$ (depicted by black), and of a strongly entangled structure at $f\left( B_{F} \left( {{\mathscr {F}}}_{N} \right) \right) =1.25$ (depicted by red). (**c**) The $\Delta F\left( {{\mathscr {S}}}_{N} \right)$ values of the $M\left( {{\mathscr {S}}}_{N} \right)$ subnetworks ${{\mathscr {S}}}_{N}^{\left( z\right) }$, $z=1,\ldots ,M\left( {{\mathscr {S}}}_{N} \right)$ at $\Delta \left( {{\mathscr {F}}}_{N} \right) >0$, for a weakly entangled structure at $f\left( B_{F} \left( {{\mathscr {F}}}_{N} \right) \right) =0.25$ (depicted by black) and the $\mu _{N} \left( {{\mathscr {S}}}_{N} ,\Delta \left( {{\mathscr {F}}}_{N} \right) \right) =\mu _{N} \left( {{\mathscr {S}}}'_{N} ,\Delta \left( {{\mathscr {F}}}_{N} \right)>0\right) >\mu _{N} \left( {{\mathscr {S}}}'_{N} ,0\right)$ average (depicted by dashed green line). (**d**) The $\Delta F\left( {{\mathscr {S}}}_{N} \right)$ values of the $M\left( {{\mathscr {S}}}_{N} \right)$ subnetworks ${{\mathscr {S}}}_{N}^{\left( z\right) }$, $z=1,\ldots ,M\left( {{\mathscr {S}}}_{N} \right)$ at $\Delta \left( {{\mathscr {F}}}_{N} \right) >0$, for a strongly entangled structure at $f\left( B_{F} \left( {{\mathscr {F}}}_{N} \right) \right) =1.25$ (depicted by black) and the $\mu _{N} \left( {{\mathscr {S}}}_{N} ,\Delta \left( {{\mathscr {F}}}_{N} \right) \right) =\mu _{N} \left( {{\mathscr {S}}}_{N}^{*} ,\Delta \left( {{\mathscr {F}}}_{N} \right)>0\right) >\mu _{N} \left( {{\mathscr {S}}}_{N}^{*} ,0\right)$ average (depicted by dashed green line).

**Noisy scenarios** The results of are depicted in Fig. 4 for strongly and weakly entangled structures at $\Delta \left( {{\mathscr {F}}}_{N} \right) >0$, $N=500$ and $M\left( {{\mathscr {S}}}_{N} \right) =5$, $\left| {{\mathscr {S}}}_{N}^{\left( z\right) } \right| =100$, $z=1,\ldots ,M\left( {{\mathscr {S}}}_{N} \right)$.

The $\Delta \left( {{\mathscr {F}}}_{N} \right) >0$ situation significantly differs from the $\Delta \left( {{\mathscr {F}}}_{N} \right) =0$ case, however the relation between the weakly and strongly entangled structures is analogous to the $\Delta \left( {{\mathscr {F}}}_{N} \right) =0$ case. As a fundamental impact of the increased noise level, the $F_{{{\mathscr {P}}}} \left( R_{i} \right)$ fidelity of the quantum nodes are decreased, therefore for a particular $R_{i}$, the probability of $F_{{{\mathscr {P}}}} \left( R_{i} \right) <F_{{{\mathscr {P}}}}^{*} \left( R_{i} \right)$ is higher compared to the $\Delta \left( {{\mathscr {F}}}_{N} \right) =0$ case. As a corollary, in Fig. 4b, the $\phi \left( F\left( {{\mathscr {F}}}_{N} \right) \right)$ values are increased for both the weakly entangled ${{\mathscr {S}}}'_{N}$ and strongly entangled ${{\mathscr {S}}}_{N}^{*}$ structures. Similarly, the $\Delta F\left( {{\mathscr {S}}}_{N} \right)$ values in Fig. 4c, d are also increased compared to the $\Delta \left( {{\mathscr {F}}}_{N} \right) =0$ case, since in the $\Delta \left( {{\mathscr {F}}}_{N} \right) >0$ setting, the values of $\Delta F_{{{\mathscr {P}}}} \left( R_{i} \right)$ of the quantum nodes pick up a positive value with higher probability than in the $\Delta \left( {{\mathscr {F}}}_{N} \right) =0$ setting for both the ${{\mathscr {S}}}'_{N}$ and ${{\mathscr {S}}}_{N}^{*}$ entangled structures. Therefore, the corresponding relations $\mu _{N} \left( {{\mathscr {S}}}'_{N} ,\Delta \left( {{\mathscr {F}}}_{N} \right)>0\right) >\mu _{N} \left( {{\mathscr {S}}}'_{N} ,0\right)$ and $\mu _{N} \left( {{\mathscr {S}}}_{N}^{*} ,\Delta \left( {{\mathscr {F}}}_{N} \right)>0\right) >\mu _{N} \left( {{\mathscr {S}}}_{N}^{*} ,0\right)$ straightforwardly follow between the weakly and strongly entangled structure of the noisy and noiseless scenarios.

### Impacts of noise on the equilibrium states of the entangled network

#### Lemma 1

(Impacts of noise on the equilibrium states of the entangled network) *For a given* $f\left( B_{F} \left( {{\mathscr {F}}}_{N} \right) \right)$, *the stable equilibrium states of the entangled network are determined only by* $\Delta \left( {{\mathscr {F}}}_{N} \right)$.

#### Proof

The proof trivially follows form the formula of (69).

The proof is concluded here. $\square$

#### Stable equilibrium states of the entangled structure at noise

This section demonstrates the results for noisy scenarios.

**Figure 5:**
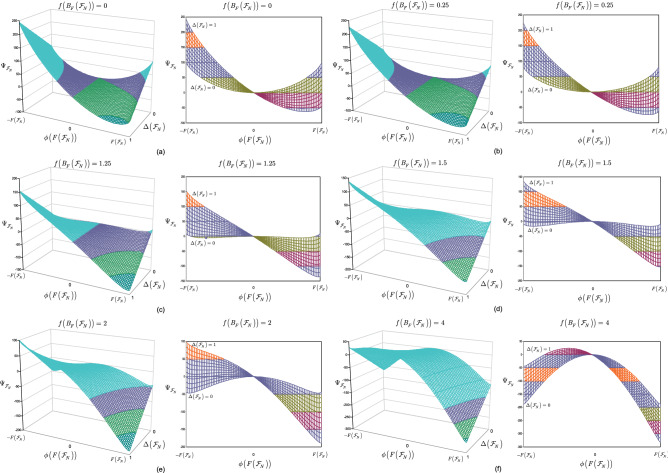
Impacts of noise on the equilibrium states of a weakly entangled structure ${{\mathscr {S}}}'_{N}$ (**a**), (b) and strongly entangled (**c**)–(**f**) quantum network ${{\mathscr {S}}}_{N}^{*}$ at a given $f\left( B_{F} \left( {{\mathscr {F}}}_{N} \right) \right)$, $\left| V\right| =100$. The stability function $\Psi _{{{\mathscr {F}}}_{N} } \left( \phi \left( F\left( {{\mathscr {F}}}_{N} \right) \right) \right)$ (left) in function of $\phi \left( F\left( {{\mathscr {F}}}_{N} \right) \right)$ and $\Delta \left( {{\mathscr {F}}}_{N} \right)$, and $\Psi _{{{\mathscr {F}}}_{N} } \left( \phi \left( F\left( {{\mathscr {F}}}_{N} \right) \right) \right)$ in function of $\Delta \left( {{\mathscr {F}}}_{N} \right)$, $\Delta \left( {{\mathscr {F}}}_{N} \right) \in \left[ 0,1\right]$, at a given $f\left( B_{F} \left( {{\mathscr {F}}}_{N} \right) \right)$ (right) identify the: (**a**) Weakly entangled quantum network, $f\left( B_{F} \left( {{\mathscr {F}}}_{N} \right) \right) =0$. (**b**) Weakly entangled quantum network, $f\left( B_{F} \left( {{\mathscr {F}}}_{N} \right) \right) =0.25$. (**c**) Strongly entangled quantum network, $f\left( B_{F} \left( {{\mathscr {F}}}_{N} \right) \right) =1.25$. (**d**) Strongly entangled quantum network, $f\left( B_{F} \left( {{\mathscr {F}}}_{N} \right) \right) =1.5$. (**e**) Strongly entangled quantum network, $f\left( B_{F} \left( {{\mathscr {F}}}_{N} \right) \right) =2$. (**f**) Strongly entangled quantum network, $f\left( B_{F} \left( {{\mathscr {F}}}_{N} \right) \right) =4$.

The stability function of the entangled network in the function of $\Delta \left( {{\mathscr {F}}}_{N} \right)$ is depicted in Fig. 5a–f. Figure 5a, b show a weakly entangled network structure, while Fig. 5c–f illustrate a strongly entangled network structure.

Analysis of ${{\mathscr {S}}}^{*} \left( N\right)$ stable equilibrium states can be found in Section A.2 of the Supplementary Information.

### Corollaries

The corollaries of the derivations are summarized in Corollaries 1, 3.

#### Corollary 1

*For any* $\Delta \left( {{\mathscr {F}}}_{N} \right) \ge 0$, *a weakly entangled network structure* ${{\mathscr {S}}}'_{N}$ *has only a global stable equilibrium state* ${{\mathscr {S}}}^{*} \left( N\right)$.

#### Corollary 2

*For* $\Delta \left( {{\mathscr {F}}}_{N} \right) =0$, *a strongly entangled network structure* ${{\mathscr {S}}}_{N}^{*}$ *has a global stable equilibrium state* ${{\mathscr {S}}}^{*} \left( N\right)$ *or two local, symmetrical equilibrium states* ${{\mathscr {S}}}^{*} \left( N\right)$, *depending on the value of* $f\left( B_{F} \left( {{\mathscr {F}}}_{N} \right) \right)$.

#### Corollary 3

*For any* $\Delta \left( {{\mathscr {F}}}_{N} \right) >0$, *a strongly entangled network structure* ${{\mathscr {S}}}_{N}^{*}$ *has a global stable equilibrium state* ${{\mathscr {S}}}^{*} \left( N\right)$ *or two local, but asymmetrical equilibrium states* ${{\mathscr {S}}}^{*} \left( N\right)$ *depending on the value of* $f\left( B_{F} \left( {{\mathscr {F}}}_{N} \right) \right)$.

### Dynamics of a local entanglement purification

#### Lemma 2

(Dynamics impacts of local entanglement purification in the quantum Internet) *The use of entanglement purification for the improvement of the entanglement fidelity only for a given subset of quantum nodes statistically decreases the total capability of the quantum network to improve the average fidelity of the network.*

#### Proof

To derive the proof via the statistical physics model, we construct a quantum repeater-level model, in the following manner.

Let $\delta \left( R_{i} \right)$ characterize the state of an *i*th quantum repeater $R_{i}$, defined as

118 $$\begin{aligned} \delta \left( R_{i} \right) =\psi B_{F} \left( P_{R_{i} } \right) , \end{aligned}$$

where $B_{F} \left( P_{R_{i} } \right)$ is the entanglement rate consumption of entanglement purification $P_{R_{i} }$ (sum of incoming and outcoming entanglement rates in $R_{i}$ associated with $P_{R_{i} }$), as

119 $$\begin{aligned} B_{F} \left( P_{R_{i} } \right) ={ \frac{1}{\left| S_{P_{R_{i} } } \right| }} \sum _{k=1}^{\left| S_{P_{R_{i} } } \right| }B_{F} \left( E_{k} \right) , \end{aligned}$$

where $S_{P_{R_{i} } }$is the set of entangled connections of $R_{i}$ associated with entanglement purification $P_{R_{i} }$, $\left| S_{P_{R_{i} } } \right|$ is the cardinality of set $S_{P_{R_{i} } }$, while $\psi$ is defined as

120 $$\begin{aligned} \psi ={ \frac{F_{{{\mathscr {P}}}}^{*} \left( R_{i} \right) }{B_{F}^{*} \left( P_{R_{i} } \right) }} , \end{aligned}$$

where $F_{{{\mathscr {P}}}}^{*} \left( R_{i} \right)$ is a target average fidelity of $R_{i}$,

121 $$\begin{aligned} F_{{{\mathscr {P}}}}^{*} \left( R_{i} \right) >F_{{{\mathscr {P}}}} \left( R_{i} \right) , \end{aligned}$$

while $F_{{{\mathscr {P}}}} \left( R_{i} \right)$ is a current average fidelity of $R_{i}$ defined via (6), and $B_{F}^{*} \left( P_{R_{i} } \right)$ is a target value of $B_{F} \left( P_{R_{i} } \right)$, as

122 $$\begin{aligned} B_{F}^{*} \left( P_{R_{i} } \right) <B_{F} \left( P_{R_{i} } \right) . \end{aligned}$$

The quantity in (120) therefore identifies a statistical cost of reaching $F_{{{\mathscr {P}}}}^{*} \left( R_{i} \right)$ in terms of entanglement rate consumption $B_{F}^{*} \left( P_{R_{i} } \right)$ (Increasing $B_{F}^{*} \left( P_{R_{i} } \right)$ at a given $F_{{{\mathscr {P}}}}^{*} \left( R_{i} \right)$ means that a higher entanglement rate consumption is needed in $R_{i}$, and as a corollary, $\psi$ is decreased.). The target values $F_{{{\mathscr {P}}}}^{*} \left( R_{i} \right)$ and $B_{F}^{*} \left( P_{R_{i} } \right)$ are considered as global quantities, i.e., (120) is considered to be the same for all quantum repeaters of the network.

Then, let $\omega$ be the ratio of the target average fidelity $F^{*} \left( {{\mathscr {F}}}_{N} \right)$ of the entanglement flow ${{\mathscr {F}}}_{N}$ of *N* and $F_{{{\mathscr {P}}}}^{*} \left( R_{i} \right)$,

123 $$\begin{aligned} \omega ={ \frac{F^{*} \left( {{\mathscr {F}}}_{N} \right) }{F_{{{\mathscr {P}}}}^{*} \left( R_{i} \right) }} , \end{aligned}$$

which quantity identifies and preserves the value of the local $F_{{{\mathscr {P}}}}^{*} \left( R_{i} \right)$ with respect to a given global $F^{*} \left( {{\mathscr {F}}}_{N} \right)$ in the statistical model.

Using (123), $F^{*} \left( {{\mathscr {F}}}_{N} \right)$ can be expressed as

124 $$\begin{aligned} F^{*} \left( {{\mathscr {F}}}_{N} \right) =\omega F_{{{\mathscr {P}}}}^{*} \left( R_{i} \right) . \end{aligned}$$

From (118) and (123), the ${{\mathscr {C}}}\left( R_{i} \right)$ capability of a given quantum repeater $R_{i}$ to improve the $F\left( {{\mathscr {F}}}_{N} \right)$ average fidelity of ${{\mathscr {F}}}_{N}$ to a target $F^{*} \left( {{\mathscr {F}}}_{N} \right)$ via an entanglement purification $P_{R_{i} }$ is defined as

125 $$\begin{aligned} {{\mathscr {C}}}\left( R_{i} \right) =\delta \left( R_{i} \right) \omega . \end{aligned}$$

Using (125), the ${{\mathscr {C}}}\left( N\right)$ capability of the entangled network *N* to improve the $F\left( {{\mathscr {F}}}_{N} \right)$ average fidelity of ${{\mathscr {F}}}_{N}$ to a target $F^{*} \left( {{\mathscr {F}}}_{N} \right)$ via $P_{R_{i} }$ in the $\left| V\right|$ nodes, $i=1,\ldots ,\left| V\right|$, of *N* is as

126 $$\begin{aligned} {{\mathscr {C}}}\left( N\right) =\sum _{i=1}^{\left| V\right| }{{\mathscr {C}}}\left( R_{i} \right) =\psi \omega B_{F} \left( P_{N} \right) , \end{aligned}$$

where $B_{F} \left( P_{N} \right)$ is the total entanglement rate consumption of entanglement purification $P_{N}$ in *N*,

127 $$\begin{aligned} B_{F} \left( P_{N} \right) =\sum _{i=1}^{\left| V\right| }B_{F} \left( P_{R_{i} } \right) , \end{aligned}$$

from which the ${\tilde{B}}_{F} \left( P_{R_{i} } \right)$ average entanglement rate consumption at $P_{N}$ for a given node is

128 $$\begin{aligned} {\tilde{B}}_{F} \left( P_{R_{i} } \right) ={ \frac{1}{\left| V\right| }} B_{F} \left( P_{N} \right) . \end{aligned}$$

Note that $\psi$ and $\omega$ in (126) are global quantities (same for all quantum nodes of the quantum network).

Then, at a given $F_{{{\mathscr {P}}}}^{*} \left( R_{i} \right)$, let $P'_{R_{i} }$ be an entanglement purification in a local $R_{i}$ with an increased target entanglement rate consumption $B_{F}^{*} \left( P'_{R_{i} } \right)$, such that

129 $$\begin{aligned} B_{F}^{*} \left( P'_{R_{i} } \right) >B_{F}^{*} \left( P_{R_{i} } \right) <B_{F} \left( P_{R_{i} } \right) , \end{aligned}$$

where $B_{F} \left( P_{R_{i} } \right)$ is given in (119).

Using $P'_{R_{i} }$, allows us to rewrite (120) as $\psi '$

130 $$\begin{aligned} \psi '={ \frac{F_{{{\mathscr {P}}}}^{*} \left( R_{i} \right) }{B_{F}^{*} \left( P'_{R_{i} } \right) }} <\psi , \end{aligned}$$

that can be rewritten as

131 $$\begin{aligned} \psi '=\psi \left( C_{1} -C_{2} B_{F} \left( P'_{N} \right) \right) , \end{aligned}$$

where $C_{1} ,C_{2} \in \left[ 0,1\right]$ are constants, $B_{F} \left( P'_{N} \right)$ is the total entanglement rate consumption of entanglement purification $P'_{N}$, as

132 $$\begin{aligned} {{B}_{F}}\left( {{{{P}'_{N}}}} \right)&=\sum \limits _{i=1}^{\left| V \right| }{{{B}_{F}}\left( {{{{P}'_{{{R}_{i}}}}}} \right) } \\&=\sum \limits _{i=1}^{\left| V \right| }{\tfrac{1}{\left| {{S}_{{{{{P}'_{{{R}_{i}}}}}}}} \right| }\sum \limits _{k=1}^{\left| {{S}_{{{{{P}'_{{{R}_{i}}}}}}}} \right| }{{{B}_{F}}\left( {{E}_{k}} \right) ,}} \end{aligned}$$

thus for a given quantum node the ${\tilde{B}}_{F} \left( P'_{R_{i} } \right)$ average entanglement rate consumption at $P'_{N}$ is

133 $$\begin{aligned} {\tilde{B}}_{F} \left( P'_{R_{i} } \right) ={ \frac{1}{\left| V\right| }} B_{F} \left( P'_{N} \right) . \end{aligned}$$

As a corollary, using (131), the state of $R_{i}$ from (118) can be rewritten at $P'_{R_{i} }$ as

134 $$\begin{aligned} {\delta }'\left( {{R}_{i}} \right)&={\psi }'{{B}_{F}}\left( {{{{P}'_{{{R}_{i}}}}}} \right) \\&=\psi \left( {{C}_{1}}-{{C}_{2}}{{B}_{F}}\left( {{{{P}'_{N}}}} \right) \right) {{B}_{F}}\left( {{{{P}'_{{{R}_{i}}}}}} \right) , \end{aligned}$$

while the ${{\mathscr {C}}}\left( R_{i} \right)$ capability of a given $R_{i}$ from (125) can be rewritten as

135 $$\begin{aligned} {{\mathscr {C}}}'\left( R_{i} \right) =\omega \psi \left( C_{1} -C_{2} B_{F} \left( P'_{N} \right) \right) B_{F} \left( P'_{R_{i} } \right) , \end{aligned}$$

while from (126), the ${{\mathscr {C}}}'\left( N\right)$ capability of the entangled network *N* to improve the $F\left( {{\mathscr {F}}}_{N} \right)$ average fidelity of ${{\mathscr {F}}}_{N}$ to a target $F^{*} \left( {{\mathscr {F}}}_{N} \right)$ via $P'_{R_{i} }$ in the $\left| V\right|$ nodes, $i=1,\ldots ,\left| V\right|$, of *N* is as

136 $$\begin{aligned} {{\mathscr {C}}}'\left( N \right)&=\sum \limits _{i=1}^{\left| V \right| }{{{\mathscr {C}}}'\left( {{R}_{i}} \right) } \\&={\psi }'\omega {{B}_{F}}\left( {{{{P}'_{N}}}} \right) \\&=\omega \psi \left( {{C}_{1}}-{{C}_{2}}{{B}_{F}}\left( {{{{P}'_{N}}}} \right) \right) {{B}_{F}}\left( {{{{P}'_{N}}}} \right) \\&=\omega \psi \left( {{C}_{1}}{{B}_{F}}\left( {{{{P}'_{N}}}} \right) -{{C}_{2}}B_{F}^{2}\left( {{{{P}'_{N}}}} \right) \right) . \end{aligned}$$

After some calculations, ${{\mathscr {C}}}'\left( N\right)$ in (136) is maximized if ${{\mathscr {C}}}'\left( R_{i} \right)$ is

137 $$\begin{aligned} {{\mathscr {C}}}'\left( R_{i} \right) ={ \frac{1}{4C_{2} \left| V\right| }} \omega \psi \left( C_{1}^{2} \right) . \end{aligned}$$

Putting (137) into (136) yields

138 $$\begin{aligned} {{\mathscr {C}}}'\left( N \right)&=\sum \limits _{i=1}^{\left| V \right| }{\tfrac{1}{4{{C}_{2}}\left| V \right| }\omega \psi \left( C_{1}^{2} \right) } \\&=\tfrac{1}{4{{C}_{2}}}\omega \psi \left( C_{1}^{2} \right) , \end{aligned}$$

from which $B_{F} \left( P'_{N} \right)$ can be found via the solution of

139 $$\begin{aligned} C_{2} B_{F}^{2} \left( P'_{N} \right) -C_{1} B_{F} \left( P'_{N} \right) -{ \frac{1}{4C_{2} }} C_{1}^{2} =0, \end{aligned}$$

which yields

140 $$\begin{aligned} B_{F} \left( P'_{N} \right) ={ \frac{C_{1} }{2C_{2} }} . \end{aligned}$$

As it can be concluded from the comparison of (135) and (137), ${{\mathscr {C}}}'\left( R_{i} \right)$ at a node-level maximization in (135), and ${{\mathscr {C}}}'\left( R_{i} \right)$ in a network-level maximization in (137), in fact, are different.

Let assume that in the quantum network, a set $\Gamma '$ of

141 $$\begin{aligned} \left| \Gamma '\right| =\left| V'\right| \end{aligned}$$

quantum nodes use purification $P'_{R_{i} }$,

142 $$\begin{aligned} \left| V'\right| =X\left| V\right| , \end{aligned}$$

where $X\in \left( 0,1\right]$, such that

143 $$\begin{aligned} B_{F} \left( P'_{N} \right) =\left( 1+\mu \right) B_{F} \left( P_{N} \right) , \end{aligned}$$

where $\mu \in \left( 0,1\right]$ is a constant, while the remaining set $\Gamma$ of

144 $$\begin{aligned} \left| \Gamma \right| =\left| V\right| -\left| V'\right| \end{aligned}$$

quantum nodes use purification $P_{R_{i} }$, with $B_{F} \left( P_{N} \right)$.

For a given $R_{i}$ from set $\Gamma$, ${{\mathscr {C}}}'\left( R_{i} \right)$ is as

145 $$\begin{aligned} {{\mathscr {C}}}'\left( R_{i} \right) =\left( 1-X\mu \right) { \frac{1}{4C_{2} \left| V\right| }} \omega \psi \left( C_{1}^{2} \right) , \end{aligned}$$

while for the total $\left| \Gamma \right|$ nodes of $\Gamma '$,

146 $$\begin{aligned} {{\mathscr {C}}}'\left( \Gamma \right)&=\left| \Gamma \right| \left( 1-X\mu \right) \tfrac{1}{4{{C}_{2}}\left| V \right| }\omega \psi \left( C_{1}^{2} \right) \\&=\left( \left| V \right| -X\left| V \right| \right) \left( 1-X\mu \right) \tfrac{1}{4{{C}_{2}}\left| V \right| }\omega \psi \left( C_{1}^{2} \right) . \end{aligned}$$

Similarly, for a given $R_{i}$ from set $\Gamma '$, ${{\mathscr {C}}}'\left( R_{i} \right)$ is as

147 $$\begin{aligned} {{\mathscr {C}}}'\left( R_{i} \right) =\left( 1-X\mu \right) \left( 1+\mu \right) { \frac{1}{4C_{2} \left| V\right| }} \omega \psi \left( C_{1}^{2} \right) , \end{aligned}$$

while for the total $\left| \Gamma '\right|$ nodes of $\Gamma '$,

148 $$\begin{aligned} {{\mathscr {C}}}'\left( {{\Gamma }'} \right)&=\left| {{\Gamma }'} \right| \left( 1-X\mu \right) \left( 1+\mu \right) \tfrac{1}{4{{C}_{2}}\left| V \right| }\omega \psi \left( C_{1}^{2} \right) \\&=X\left| V \right| \left( 1-X\mu \right) \left( 1+\mu \right) \tfrac{1}{4{{C}_{2}}\left| V \right| }\omega \psi \left( C_{1}^{2} \right) . \end{aligned}$$

For the total network $N=\Gamma \bigcup \Gamma '$, from (146) and (148), ${{\mathscr {C}}}''\left( N\right)$ is evaluated as

149 $$\begin{aligned} {{\mathscr {C}}}''\left( N \right)&={{\mathscr {C}}}'\left( \Gamma \right) +{{\mathscr {C}}}'\left( {{\Gamma }'} \right) \\&=\left( \left( \left| V \right| -X\left| V \right| \right) \left( 1-X\mu \right) +X\left| V \right| \left( 1-X\mu \right) \left( 1+\mu \right) \right) \tfrac{1}{4{{C}_{2}}\left| V \right| }\omega \psi \left( C_{1}^{2} \right) \\&=\left| V \right| \left( 1-{{X}^{2}}{{\mu }^{2}} \right) \tfrac{1}{4{{C}_{2}}\left| V \right| }\omega \psi \left( C_{1}^{2} \right) \\&=\left( 1-{{X}^{2}}{{\mu }^{2}} \right) \tfrac{1}{4{{C}_{2}}}\omega \psi \left( C_{1}^{2} \right) . \end{aligned}$$

From (147) follows, that the improvement of the local capability requires the parameterization $\mu >0$ and $0<X<{1/ \left( 1+\mu \right) }$. On the other hand, from (149) follows that for any $X>0$ and $\mu >0$, the ${{\mathscr {C}}}'\left( N\right)$ capability of the entangled network *N* from (138) is decreased by a $\partial$ ratio to

150 $$\begin{aligned} {{\mathscr {C}}}''\left( N\right) ={{\mathscr {C}}}'\left( N\right) \partial , \end{aligned}$$

where

151 $$\begin{aligned} \partial ={ \frac{{{\mathscr {C}}}''\left( N\right) }{{{\mathscr {C}}}'\left( N\right) }} =\left( 1-X^{2} \mu ^{2} \right) , \end{aligned}$$

that immediately proves that statistically, the capability of the entangled network *N* to improve the $F\left( {{\mathscr {F}}}_{N} \right)$ average fidelity of ${{\mathscr {F}}}_{N}$ to a target $F^{*} \left( {{\mathscr {F}}}_{N} \right)$ is decreased if an improved entanglement purification $P'_{R_{i} }$ with $B_{F} \left( P'_{N} \right) >\left( 1+\mu \right) B_{F} \left( P_{N} \right)$ is applied only to a local subset $\Gamma '$ of quantum nodes in the quantum network, while the remaining set $\Gamma$ quantum nodes use purification $P_{R_{i} }$, with $B_{F} \left( P_{N} \right)$.

The proof is concluded here. $\square$

## Entanglement flow dynamics

This section derives the dynamics of optimal entanglement flow in the entangled structures of the quantum Internet at fluctuating entangled connections and quantum nodes. The derivations utilize the fundamentals of spectral graph theory^141–143^.

### Theorem 3

(Maximally allowed fluctuations in entangled structures for seamless entanglement flow) *For the total* *Q* *paths of* *N*, *the* ${{\mathscr {F}}}_{N}$ *entanglement flow is seamless,* ${{\mathscr {F}}}_{N} =\tilde{{{\mathscr {F}}}}_{N}$, *if* $\varphi \left( E_{s} \right) \le \varphi ^{*} \left( E_{s} \right)$ *for* $s=1,\ldots ,\sum _{j=1}^{Q}\left| S_{{{\mathscr {P}}}_{j} } \right|$, *where* $\varphi ^{*} \left( E_{s} \right)$ *is an upper bound on* $\varphi \left( E_{s} \right)$ *in a* ${{\mathscr {S}}}^{*} \left( N\right)$ *stable equilibrium state*, $\varphi \left( E_{s} \right) \le \varphi ^{*} \left( E_{s} \right)$.

### Proof

A main challenge here is the determination of the $\varphi \left( E_{s} \right)$ fluctuation coefficients of the entangled connections $E=\left\{ E_{s} \right\} _{s=1}^{\sum _{j=1}^{Q}\left| S_{{{\mathscr {P}}}_{j} } \right| }$. As we prove, the fluctuation coefficients of the entangled connections straightforwardly can be yielded from the structure of the entangled network *N*, in the following manner.

Let $\varphi \left( E_{s} \right)$ be the fluctuation of an entangled connection $E_{s} \left( x,y\right)$ between nodes *x* and *y*, defined as

152 $$\begin{aligned} \varphi \left( E_{s} \right) =\left| \varphi \left( x\right) -\varphi \left( y\right) \right| , \end{aligned}$$

where $\varphi \left( x\right)$ and $\varphi \left( y\right)$ are the fluctuations associated with *x* and *y*, defined as

153 $$\begin{aligned} \varphi \left( x\right) =f\left( B_{F} \left( x\right) \right) -f \left( B_{F} \left( {{\mathscr {S}}}^{*} \left( N\right) \right) \right) , \end{aligned}$$

where $f\left( B_{F} \left( x\right) \right)$ is the normalized outcoming entanglement rate of *x* on connection $E_{s} \left( x,y\right)$,

154 $$\begin{aligned} f\left( B_{F} \left( x\right) \right) =B_{F} \left( x\right) { \frac{1}{B_{F}^{*} \left( {{\mathscr {F}}}_{N} \right) }} , \end{aligned}$$

thus $\varphi \left( x\right)$ from (153) is as

155 $$\begin{aligned} \varphi \left( x \right)&={{B}_{F}}\left( x \right) \tfrac{1}{B_{F}^{*}\left( {{{\mathscr {F}}}_{N}} \right) }-{{B}_{F}}\left( {{{\mathscr {F}}}_{N}} \right) \tfrac{1}{B_{F}^{*}\left( {{{\mathscr {F}}}_{N}} \right) } \\&=\tfrac{1}{B_{F}^{*}\left( {{{\mathscr {F}}}_{N}} \right) }\left( {{B}_{F}}\left( x \right) -{{B}_{F}}\left( {{{\mathscr {F}}}_{N}} \right) \right) , \end{aligned}$$

while

156 $$\begin{aligned} \varphi \left( y\right) =f\left( B_{F} \left( y\right) \right) -f \left( B_{F} \left( {{\mathscr {S}}}^{*} \left( N\right) \right) \right) , \end{aligned}$$

where $f\left( B_{F} \left( y\right) \right)$ is the normalized incoming entanglement rate of *y* on connection $E_{s} \left( x,y\right)$, as

157 $$\begin{aligned} f\left( B_{F} \left( y\right) \right) =B_{F} \left( y\right) { \frac{1}{B_{F}^{*} \left( {{\mathscr {F}}}_{N} \right) }} . \end{aligned}$$

thus $\varphi \left( y\right)$ from (156) can be rewritten as

158 $$\begin{aligned} \varphi \left( y \right)&={{B}_{F}}\left( y \right) \tfrac{1}{B_{F}^{*}\left( {{{\mathscr {F}}}_{N}} \right) }-{{B}_{F}}\left( {{{\mathscr {F}}}_{N}} \right) \tfrac{1}{B_{F}^{*}\left( {{{\mathscr {F}}}_{N}} \right) } \\&=\tfrac{1}{B_{F}^{*}\left( {{{\mathscr {F}}}_{N}} \right) }\left( {{B}_{F}}\left( y \right) -{{B}_{F}}\left( {{{\mathscr {F}}}_{N}} \right) \right) . \end{aligned}$$

Thus, $\varphi \left( E_{s} \right)$ from (152) can be rewritten as

159 $$\begin{aligned} \varphi \left( {{E}_{s}} \right)&=\left| f\left( {{B}_{F}}\left( x \right) \right) -f\left( {{B}_{F}}\left( {{{\mathscr {S}}}^{*}}\left( N \right) \right) \right) -\left( f\left( {{B}_{F}}\left( y \right) \right) -f\left( {{B}_{F}}\left( {{{\mathscr {S}}}^{*}}\left( N \right) \right) \right) \right) \right| \\&=\left| f\left( {{B}_{F}}\left( x \right) \right) -f\left( {{B}_{F}}\left( y \right) \right) \right| \\&=\left| \tfrac{1}{B_{F}^{*}\left( {{{\mathscr {F}}}_{N}} \right) }\left( {{B}_{F}}\left( x \right) -{{B}_{F}}\left( y \right) \right) \right| . \end{aligned}$$

The $\varphi \left( E_{s} \right)$ and the $\varphi ^{*} \left( E_{s} \right)$ critical coefficients are determined as follows.

For a given $E_{s} \left( R_{i} ,R_{k} \right)$ between $R_{i}$ and $R_{k}$, let $\omega _{ik} >0$ be defined as the sum of normalized entanglement throughput of all paths over $E_{s} \left( R_{i} ,R_{k} \right)$, as

160 $$\begin{aligned} {{\omega }_{ik}}&=\sum \limits _{j=1}^{Q}{f\left( {{B}_{F,{{{\mathscr {P}}}_{j}}}}\left( {{E}_{s}}\left( {{R}_{i}},{{R}_{k}} \right) \right) \right) } \\&=\sum \limits _{j=1}^{Q}{{{B}_{F,{{{\mathscr {P}}}_{j}}}}\left( {{E}_{s}} \right) \tfrac{1}{B_{F,{{{\mathscr {P}}}_{j}}}^{*}\left( {{E}_{s}} \right) },} \end{aligned}$$

where $B_{F,{{\mathscr {P}}}_{j} } \left( E_{s} \right)$ is as in (8), while $B_{F,{{\mathscr {P}}}_{j} }^{*} \left( E_{s} \right)$ is a critical bound on $B_{F,{{\mathscr {P}}}_{j} } \left( E_{s} \right)$.

Then, let

161 $$\begin{aligned} \vec {W}=\left[ W_{ij} \right] \end{aligned}$$

be a $\left| V\right| \times \left| V\right|$ matrix, defined as

162 $$\begin{aligned} W_{ij} =\left\{ \begin{array}{l} {z_{i} \omega _{ik} ,\mathrm{if}\; E_{s} \in E} \\ {0,\mathrm{if}\; E_{s} \not \in E} \end{array}\right. , \end{aligned}$$

where $z_{i}$ is a constraint for $R_{i}$, such that a symmetry condition

163 $$\begin{aligned} z_{i} \omega _{ik} =z_{k} \omega _{ki} \end{aligned}$$

holds, where $z_{k}$ is a constraint for $R_{k}$. The scaling factors formulate $\vec {Z}$ as

164 $$\begin{aligned} \vec {Z}=\mathrm{diag}\,\left( z_{1} ,\ldots ,z_{\left| V\right| } \right) . \end{aligned}$$

For a given $R_{i}$, let ${{\mho }_{i}}$ be the set of all entangled connections of $R_{i}$, and let $\chi _{i}$ be defined as

165 $$\begin{aligned} \chi _{i} =\sum _{k\in {{\mho }_{i}}}\omega _{ik} , \end{aligned}$$

from which a matrix $\vec {X}$ is defined for the $\left| V\right|$ quantum nodes of *N*, as

166 $$\begin{aligned} \vec {X}=\mathrm{diag}\left( \chi _{1} ,\ldots ,\chi _{\left| V\right| } \right) . \end{aligned}$$

From (161) and (166), the symmetric ${{\mathscr {L}}}\left( N\right)$ Laplacian^141^ of the undirected entangled quantum network *N* is defined as

167 $$\begin{aligned} {{\mathscr {L}}}\left( N\right) =\vec {X}-\vec {W}. \end{aligned}$$

Using the condition from (163), ${{\mathscr {L}}}\left( N\right)$ can be rewritten as an asymmetric and symmetrizable^141–143^ Laplacian ${{\mathscr {L}}}^{*} \left( N\right)$, as

168 $$\begin{aligned} {{\mathscr {L}}}^{*} \left( N\right) =\left( \vec {Z}\right) ^{-1} {{\mathscr {L}}}\left( N\right) . \end{aligned}$$

Note, that for a general Laplacian $\left\langle {{\mathscr {L}}}\left( N\right) \right\rangle$ of a directed entangled quantum network *N*,

169 $$\begin{aligned} \left\langle {{\mathscr {L}}}\left( N\right) \right\rangle =\vec {X}-\left\langle \vec {W}\right\rangle , \end{aligned}$$

with

170 $$\begin{aligned} \left\langle W_{ij} \right\rangle =\left\{ \begin{array}{l} {z_{i} \omega _{ik} ,{\mathrm{if}}\; E_{s} \left( i\rightarrow k\right) \in E} \\ {0,{\mathrm{if}}\; E_{s} \not \in E} \end{array}\right. , \end{aligned}$$

the relation in (163) is not a required condition. If (163) is not satisfied, then the general $\left\langle {{\mathscr {L}}}\left( N\right) \right\rangle$ is unsymmetrizable^141^.

Then, let $\vec {\varphi }\left( N\right)$ be the node fluctuations in *N*

171 $$\begin{aligned} \vec {\varphi }\left( N\right) =\left( \varphi \left( 1\right) ,\ldots ,\varphi \left( \left| V\right| \right) \right) ^{T} , \end{aligned}$$

subject to be found.

Then,

172 $$\begin{aligned} {{\mathscr {L}}}^{*} \left( N\right) \vec {\varphi }\left( N\right) =\lambda \vec {\varphi }\left( N\right) , \end{aligned}$$

where $\lambda$ is an eigenvalue of ${{\mathscr {L}}}^{*} \left( N\right)$. Thus, $\vec {\varphi }\left( N\right)$ from (171) can be rewritten as an eigenvector that is associated with $\lambda$.

The $\lambda$ eigenvalues of ${{\mathscr {L}}}^{*} \left( N\right)$ are evaluated from (167) and (164) via the relation

173 $$\begin{aligned} S\left( {{\mathscr {L}}}\left( N\right) \right) =\left( \vec {Z}\right) ^{{1/ 2}} {{\mathscr {L}}}^{*} \left( N\right) \left( \vec {Z}\right) ^{{-1/2} } =\left( \vec {Z}\right) ^{{-1/2}} {{\mathscr {L}}}\left( N\right) \left( \vec {Z}\right) ^{{-1/2}} , \end{aligned}$$

where $S\left( {{\mathscr {L}}}\left( N\right) \right)$ is the scaled Laplacian ${{\mathscr {L}}}\left( N\right)$.

Then, (173) can be evaluated further as

174 $$\begin{aligned} {{\left( {\vec {Z}} \right) }^{{1}/{2}\;}}{{{\mathscr {L}}}^{*}}\left( N \right) \vec {\varphi }\left( N \right)&=S\left( {\mathscr {L}}\left( N \right) \right) \left( {{\left( {\vec {Z}} \right) }^{{1}/{2}\;}}\vec {\varphi }\left( N \right) \right) \\&=\lambda \left( {{\left( {\vec {Z}} \right) }^{{1}/{2}\;}}\vec {\varphi }\left( N \right) \right) \\&=\lambda \left( \vec {\xi }\left( N \right) \right) , \end{aligned}$$

thus $S\left( {{\mathscr {L}}}\left( N\right) \right)$ has the same eigenvalues as ${{\mathscr {L}}}^{*} \left( N\right)$, with eigenvector $\vec {\xi }\left( N\right)$,

175 $$\begin{aligned} \vec {\xi }\left( N\right) =\left( \vec {Z}\right) ^{{1/2}} \vec {\varphi }\left( N\right) =\left( \xi \left( 1\right) ,\ldots ,\xi \left( \left| V\right| \right) \right) ^{T} . \end{aligned}$$

The eigenvalues of $S\left( {{\mathscr {L}}}\left( N\right) \right)$ are nonnegative, since

176 $$\begin{aligned} \left( \vec {\xi }\left( N\right) \right) ^{T} S\left( {{\mathscr {L}}}\left( N\right) \right) \vec {\xi }\left( N\right) =\sum _{E\left( i,k\right) \in E}z_{i} \omega _{ik} \left( { \frac{\xi \left( i\right) }{\sqrt{z_{i} } }} -{ \frac{\xi \left( k\right) }{\sqrt{z_{k} } }} \right) ^{2} \ge 0, \end{aligned}$$

where $z_{i}$ is an *i*th element of $\vec {Z}$ (see (164)).

Then, let $\vec {\gamma }_{i}$ be an orthonormal eigenvector associated with an *i*th eigenvalue $\lambda _{i}$ (eigenbasis of $S\left( {{\mathscr {L}}}\left( N\right) \right)$), $i=1,\ldots ,\left| V\right|$ as

177 $$\begin{aligned} S\left( {{\mathscr {L}}}\left( N\right) \right) \vec {\gamma }_{i} =\lambda _{i} \vec {\gamma }_{i} , \end{aligned}$$

such that

178 $$\begin{aligned} \vec {\gamma }_{u} \vec {\gamma }_{v} =\delta _{uv} , \end{aligned}$$

where $\delta _{uv}$ is the Kronecker delta^141^.

Using (177), the eigenvalues can be determined via $\vec {\gamma }_{i}$, from which $\vec {\varphi }\left( N\right)$ is straightforwardly yielded by (172). Thus, the $\varphi \left( E_{s} \right)$ fluctuation (152) of an entangled connection $E_{s}$ can be quantified in an exact form.

Then, let ${{\mathscr {S}}}^{*} \left( N\right)$ be a stable equilibrium state of *N* with an entanglement flow rate $B_{F} \left( {{\mathscr {S}}}^{*} \left( N\right) \right)$, and let $f\left( B_{F} \left( {{\mathscr {S}}}^{*} \left( N\right) \right) \right)$ be the normalized entanglement rate $B_{F} \left( {{\mathscr {F}}}_{N} \right)$ of flow ${{\mathscr {F}}}_{N}$ in ${{\mathscr {S}}}^{*} \left( N\right)$, defined as

179 $$\begin{aligned} f \left( B_{F} \left( {{\mathscr {S}}}^{*} \left( N\right) \right) \right) =B_{F} \left( {{\mathscr {F}}}_{N} \right) { \frac{1}{B_{F}^{*} \left( {{\mathscr {F}}}_{N} \right) }} >0, \end{aligned}$$

where $B_{F}^{*} \left( {{\mathscr {F}}}_{N} \right)$ is a critical bound on $B_{F} \left( {{\mathscr {F}}}_{N} \right)$ in ${{\mathscr {S}}}^{*} \left( N\right)$.

The problem then is the determination of the upper bound $\varphi ^{*} \left( E_{s} \right)$ on (152) for all entangled connections, such that for a given $E_{s}$

180 $$\begin{aligned} \varphi \left( E_{s} \right) \le \varphi ^{*} \left( E_{s} \right) . \end{aligned}$$

We show that of $\varphi ^{*} \left( E_{s} \right)$ can be evaluated from the $\left\langle {{\mathscr {L}}}\left( N\right) \right\rangle$ Laplacian of the entangled quantum network *N*, since for any seamless $\tilde{{{\mathscr {F}}}}_{N}$ entanglement flow ${{\mathscr {F}}}_{N}^{*}$, the $\left\langle {{\mathscr {L}}}\left( N\right) \right\rangle$ general Laplacian of the entangled quantum network *N* is decomposable^141^ as

181 $$\begin{aligned} \left\langle {{\mathscr {L}}}\left( N\right) \right\rangle ={{\mathscr {L}}}^{*} \left( N\right) +\zeta _{{{\mathscr {L}}}\left( N\right) } , \end{aligned}$$

where ${{\mathscr {L}}}^{*} \left( N\right)$ is a symmetrizable Laplacian (168), while $\zeta _{{{\mathscr {L}}}\left( N\right) }$ is a residual Laplacian, such that

182 $$\begin{aligned} \zeta _{{{\mathscr {L}}}\left( N\right) } =\vec {0}, \end{aligned}$$

where $\vec {0}$ is a null matrix.

Let $N\left( t\right)$ be the state of the entangled network at a particular *t*, $t=1,\ldots ,T$ . Then, from (181), the dynamics of the $\vec {\varphi }\left( N\left( t\right) \right)$ fluctuation coefficients (171) of the entangled structure can be evaluated as

183 $$\begin{aligned} \tfrac{{{d}^{2}}\vec {\varphi }\left( N\left( t \right) \right) }{d{{t}^{2}}}&=-\left\langle {\mathscr {L}}\left( N \right) \right\rangle \vec {\varphi }\left( N\left( t \right) \right) \\&=-\left( {{{\mathscr {L}}}^{*}}\left( N \right) +{{\zeta }_{{\mathscr {L}}\left( N \right) }} \right) \vec {\varphi }\left( N\left( t \right) \right) \\&=-\left( {{{\mathscr {L}}}^{*}}\left( N \right) +\vec {0} \right) \vec {\varphi }\left( N\left( t \right) \right) , \end{aligned}$$

with

184 $$\begin{aligned} \vec {\xi }\left( N\left( t\right) \right) =\left( \vec {Z}\right) ^{{1/2}} \vec {\varphi }\left( N\left( t\right) \right) , \end{aligned}$$

and with a scaled Laplacian $S\left( \left\langle {{\mathscr {L}}}\left( N\right) \right\rangle \right)$ as

185 $$\begin{aligned} S\left( \left\langle {\mathscr {L}}\left( N \right) \right\rangle \right)&={{\left( {\vec {Z}} \right) }^{{1}/{2}\;}}\left\langle {\mathscr {L}}\left( N \right) \right\rangle {{\left( {\vec {Z}} \right) }^{{-1}/{2}\;}} \\&={{\left( {\vec {Z}} \right) }^{{1}/{2}\;}}{{{\mathscr {L}}}^{*}}\left( N \right) {{\left( {\vec {Z}} \right) }^{{-1}/{2}\;}}+{{\left( {\vec {Z}} \right) }^{{1}/{2}\;}}\vec {0}{{\left( {\vec {Z}} \right) }^{{-1}/{2}\;}} \\&={{\left( {\vec {Z}} \right) }^{{1}/{2}\;}}{{{\mathscr {L}}}^{*}}\left( N \right) {{\left( {\vec {Z}} \right) }^{{-1}/{2}\;}}+\vec {0} \\&=S\left( {{{\mathscr {L}}}^{*}}\left( N \right) \right) +S\left( {{\zeta }_{{\mathscr {L}}\left( N \right) }} \right) , \end{aligned}$$

such that $S\left( \zeta _{{{\mathscr {L}}}\left( N\right) } \right) =\vec {0}$, and

186 $$\begin{aligned} \tfrac{{{d}^{2}}\vec {\xi }\left( N\left( t \right) \right) }{d{{t}^{2}}}&=-S\left( \left\langle {\mathscr {L}}\left( N \right) \right\rangle \right) \vec {\xi }\left( N\left( t \right) \right) \\&=-\left( S\left( {{{\mathscr {L}}}^{*}}\left( N \right) \right) \right) \vec {\xi }\left( N\left( t \right) \right) , \end{aligned}$$

and

187 $$\begin{aligned} \tfrac{{{d}^{2}}\vec {\xi }\left( N\left( t \right) \right) }{d{{t}^{2}}}-{{C}_{d}}\tfrac{d\vec {\xi }\left( N\left( t \right) \right) }{dt}&=-S\left( \left\langle {\mathscr {L}}\left( N \right) \right\rangle \right) \vec {\xi }\left( N\left( t \right) \right) \\&=S\left( {{{\mathscr {L}}}^{*}}\left( N \right) \right) \vec {\xi }\left( N\left( t \right) \right) , \end{aligned}$$

where $C_{d} \ge 0$ is a constant ((187) is the typical diffusive wave equation on a graph, where the graph Laplacian plays the role of the ${{\nabla }^{2}}$ operator.).

Since (184) can be rewritten via the $\vec {\gamma }_{i}$ eigenbasis of $S\left( \left\langle {{\mathscr {L}}}\left( N\right) \right\rangle \right) =S\left( {{\mathscr {L}}}^{*} \left( N\right) \right)$ as

188 $$\begin{aligned} \vec {\xi }\left( N\left( t\right) \right) =\sum _{i=1}^{\left| V\right| }A_{i} \left( t\right) \vec {\gamma }_{i} , \end{aligned}$$

where $A_{i} \left( t\right)$ is defined via the relation of

189 $$\begin{aligned} { \frac{d^{2} A_{i} \left( t\right) }{dt^{2} }} =-\lambda _{i} A_{i} \left( t\right) , \end{aligned}$$

as

190 $$\begin{aligned} A_{i} \left( t\right) =\tau _{i} \exp \left( \pm i\alpha _{i} t\right) , \end{aligned}$$

where

191 $$\begin{aligned} \tau _{i} =\left| A_{i} \right| \exp \left( i\theta _{i} \right) , \end{aligned}$$

where $\left| A_{i} \right|$ is the fluctuation amplitude, and

192 $$\begin{aligned} -\pi <\theta _{i} \le \pi , \end{aligned}$$

is the fluctuation phase, while

193 $$\begin{aligned} \alpha _{i} =\sqrt{\lambda _{i} } . \end{aligned}$$

As follows, $\vec {\varphi }\left( N\left( t\right) \right)$ can be rewritten as

194 $$\begin{aligned} \vec {\varphi }\left( N\left( t \right) \right)&={{\left( {\vec {Z}} \right) }^{{-1}/{2}\;}}\left( \sum \limits _{i=1}^{\left| V \right| }{{{A}_{i}}\left( t \right) {{{\vec {\gamma }}}_{i}}} \right) \\&={{\left( {\vec {Z}} \right) }^{{-1}/{2}\;}}\left( \sum \limits _{i=1}^{\left| V \right| }{\left( \left| {{A}_{i}} \right| \exp \left( i{{\theta }_{i}} \right) \right) \exp \left( \pm i{{\alpha }_{i}}t \right) {{{\vec {\gamma }}}_{i}}} \right) . \end{aligned}$$

From (194) follows, that the determination of the upper bounds $\vec {\varphi }^{*} \left( N\left( t\right) \right)$ on $\vec {\varphi }\left( N\left( t\right) \right)$ is directly related with the values of $A_{i} \left( t\right)$. After some calculations, $A_{i} \left( t\right)$ can be rewritten as

195 $$\begin{aligned} A_{i} \left( t\right) =\tau _{i} \exp \left( -\left( { \frac{C_{d} }{2}} \pm \sqrt{\Upsilon } \sin \left( { \frac{\theta }{2}} \right) \right) t\pm i\sqrt{\Upsilon } \cos \left( { \frac{\theta }{2}} \right) t\right) , \end{aligned}$$

where $$\Upsilon \ge 0$$.

Thus, the critical condition

196 $$\begin{aligned} \varphi _{i} \left( N\left( t\right) \right) <\varphi _{i}^{*} \left( N\left( t\right) \right) \end{aligned}$$

is yielded via (195) if only

197 $$\begin{aligned} \sqrt{\Upsilon} \left|\sin \left( {{\frac{\theta}{2}}} \right) \right| \le {{\frac{C_{d}}{2}}} .\end{aligned}$$

It also can be verified that (197) holds for a general $\left\langle {{\mathscr {L}}}\left( N\right) \right\rangle$ (see (181)), if only $\zeta _{{{\mathscr {L}}}\left( N\right) } =\vec {0}$, which condition is given in (182).

We recall the definition of seamless entanglement flow from (15). It can be straightforwardly verified that relation $\varphi \left( E_{s} \right) \le \varphi ^{*} \left( E_{s} \right)$ is determined via (197) for all entangled connections of the quantum network. Therefore, if $\varphi _{i} \left( N\left( t\right) \right)$ is selected for all the $\left| V\right|$ quantum nodes of the quantum network such that (197) is satisfied for all $A_{i} \left( t\right)$, $i=1,\ldots ,\left| V\right|$, then $\varphi \left( E_{s} \right) \le \varphi ^{*} \left( E_{s} \right)$ holds for all entangled connections.

The proof is concluded here. $\square$

Additional results are included in Section A.3 of the Supplementary Information.

## Conclusions

The quantum Internet is an adequate answer to the computational power that becomes available via quantum computers. Here, we evaluated and quantified the dynamics of the entangled network structures of the quantum Internet. We proved the equilibrium states of entangled network structures and derived the effects of noise on the equilibrium states of the entangled network to provide stable quantum communications. We identified the attributes of weakly and strongly entangled structures of the quantum Internet and derived the dynamic effects of local entanglement purification in the global entangled structure of the quantum Internet. The model is independent of the actual physical implementations and it can be applied within the heterogeneous experimental structures of a global-scale quantum Internet.

### Ethics statement

This work did not involve any active collection of human data.

## Supplementary Information

Supplementary Information.

## Data Availability

This work does not have any experimental data.
