# Supplementary material for: Dynamics of entangled networks of the quantum Internet
Source: Sci Rep. 2020 Jul 31;10:12909. doi: 10.1038/s41598-020-68498-x (PMC7395178; doi:10.1038/s41598-020-68498-x)
Supplement: Supplementary file 1 — Supplementary Information. [file 41598_2020_68498_MOESM1_ESM.pdf]

Dynamics of Entangled Networks of the Quantum Internet

Laszlo Gyongyosi[1,2,3,*]

[1]School of Electronics and Computer Science, University of Southampton, Southampton, SO17 1BJ, UK
[2]Department of Networked Systems and Services, Budapest University of Technology and Economics, Budapest, H-1117 Hungary
[3]MTA-BME Information Systems Research Group, Hungarian Academy of Sciences, Budapest, H-1051 Hungary
[*]gyongyosi@hit.bme.hu

ABSTRACT

Entangled quantum networks are a fundamental of any global-scale quantum Internet. Here, a mathematical model is developed to quantify the dynamics of entangled network structures and entanglement flow in the quantum Internet. The analytical solutions of the model determine the equilibrium states of the entangled quantum networks and characterize the stability, fluctuation attributes, and dynamics of entanglement flow in entangled network structures. We demonstrate the results of the model through various entangled structures and quantify the dynamics.

A Appendix

A.1 Stability Function Comparison

Fig. A.1 compares the stability $\Psi_{\mathscr{F}_N}(\phi(F(\mathscr{F}_N)))$ (left) of the entangled structure and the stable $\mathscr{S}^*(N)$ equilibrium states of the entangled quantum network N (right) for a large-scaled values of $f(B_F(\mathscr{F}_N))$ at noiseless and noisy network scenarios.

A.2 Stable Equilibrium States

The $\mathscr{S}^*(N)$ stable equilibrium states are depicted in Fig. A.2. As it can be concluded from the results, for any $\Delta(\mathscr{F}_N)$, the weakly entangled structure $\mathscr{S}'_N$ has only a global stable equilibrium state $\mathscr{S}^*(N)$ in Fig. A.2(a)-(b). On the other hand, the strongly entangled structures $\mathscr{S}^*_N$ in Fig. A.2(c)-(f) have two local asymmetrical equilibrium states $\mathscr{S}^*(N)$ from some $\Delta(\mathscr{F}_N)$.

A.3 Entanglement Flow Dynamics

A.3.1 Seamless Optimal Entanglement Flow at Fluctuating Entangled Connections

Lemma A.1 *(Seamless optimal entanglement flow in the quantum Internet at fluctuating entangled connections). For the total Q paths of N, the $\mathscr{F}_N$ entanglement flow is seamless optimal, $\mathscr{F}_N = \mathscr{F}^*_N$, if $f(B_F(\mathscr{S}^*(N))) \geq 1$ and $\varphi(E_s) \leq \varphi^*(E_s)$ for $s = 1,\ldots,\sum_{j=1}^{Q}\left|S_{\mathscr{P}_j}\right|$.*

Proof. Recalling the condition of $B_F(\mathscr{F}_N) \geq B'_F(\mathscr{S}^*(N))$, $f(B_F(\mathscr{S}^*(N))) \geq 1$ for $f(B_F(\mathscr{S}^*(N)))$ straightforwardly follows. Then, from the proof of Theorem 3, the $\mathscr{L}^*(N)$ Laplacian of the entangled quantum network N is always symmetrizable for any seamless optimal entanglement flow $\mathscr{F}^*_N$, which determines $\varphi^*(E_s)$ for $s = 1,\ldots,\sum_{j=1}^{Q}\left|S_{\mathscr{P}_j}\right|$.

The proof is concluded here. ■

The notations of the fluctuation dynamics analysis of the entangled structure are summarized in Fig. A.3.

A.3.2 Entangled Network Structure with Zero Fluctuations

Lemma A.2 *(Determining an entangled network structure with zero fluctuations). At a given N with a set $E = \{E_s\}_{s=1}^{\sum_{j=1}^{Q}\left|S_{\mathscr{P}_j}\right|}$ of entangled connections, there exists an entangled connection structure $E^* = \{E_s\}_{s=1}^{\sum_{j=1}^{Q}\left|S^*_{\mathscr{P}_j}\right|}$, for which $\varphi^*(E_s) = 0$, $s = 1,\ldots,\left|S^*_{\mathscr{P}_j}\right|$, where $S^*_{\mathscr{P}_j}$ is an connection set of a j-th path $\mathscr{P}_j$.*

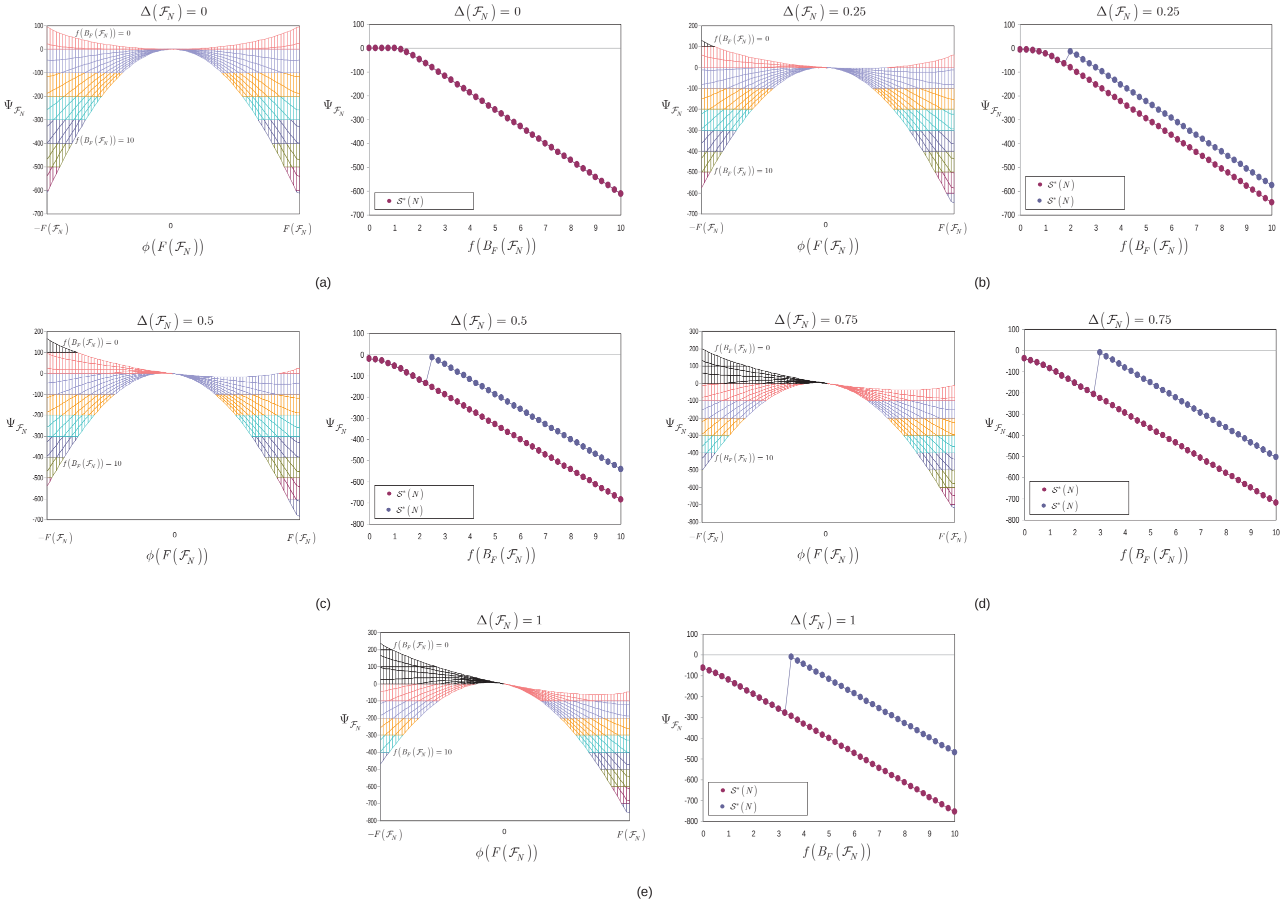

Figure A.1. The stability function $\Psi_{\mathscr{F}_N}(\phi(F(\mathscr{F}_N)))$ (left) and the stable $\mathscr{S}^*(N)$ equilibrium states of the entangled quantum network N (right) for a large-scaled values of $f(B_F(\mathscr{F}_N))$, $f(B_F(\mathscr{F}_N)) = [0,10]$, at $\Delta(\mathscr{F}_N) \in [0,1]$, $|V| = 100$. (a) As $\Delta(\mathscr{F}_N) = 0$, the stable equilibrium states of the entangled structure are symmetrical for all $f(B_F(\mathscr{F}_N))$, thus the stable equilibrium states for $f(B_F(\mathscr{F}_N)) > 1$ are overlapped in the right figure. (b)-(d) As $\Delta(\mathscr{F}_N)$ increases, the entangled structure has one stable equilibrium state only for low values of $f(B_F(\mathscr{F}_N))$. For a higher values of $f(B_F(\mathscr{F}_N))$ the two stable equilibrium states are asymmetrical, thus the stable equilibrium states in the right figure are distinguished into red and blue dots for a particular $f(B_F(\mathscr{F}_N))$. (e) As $\Delta(\mathscr{F}_N) \to 1$, $f(B_F(\mathscr{F}_N)) \leq 3$, the entangled structure has one stable state, while if $f(B_F(\mathscr{F}_N)) > 3$, then the entangled network has two asymmetrical stable equilibrium states.

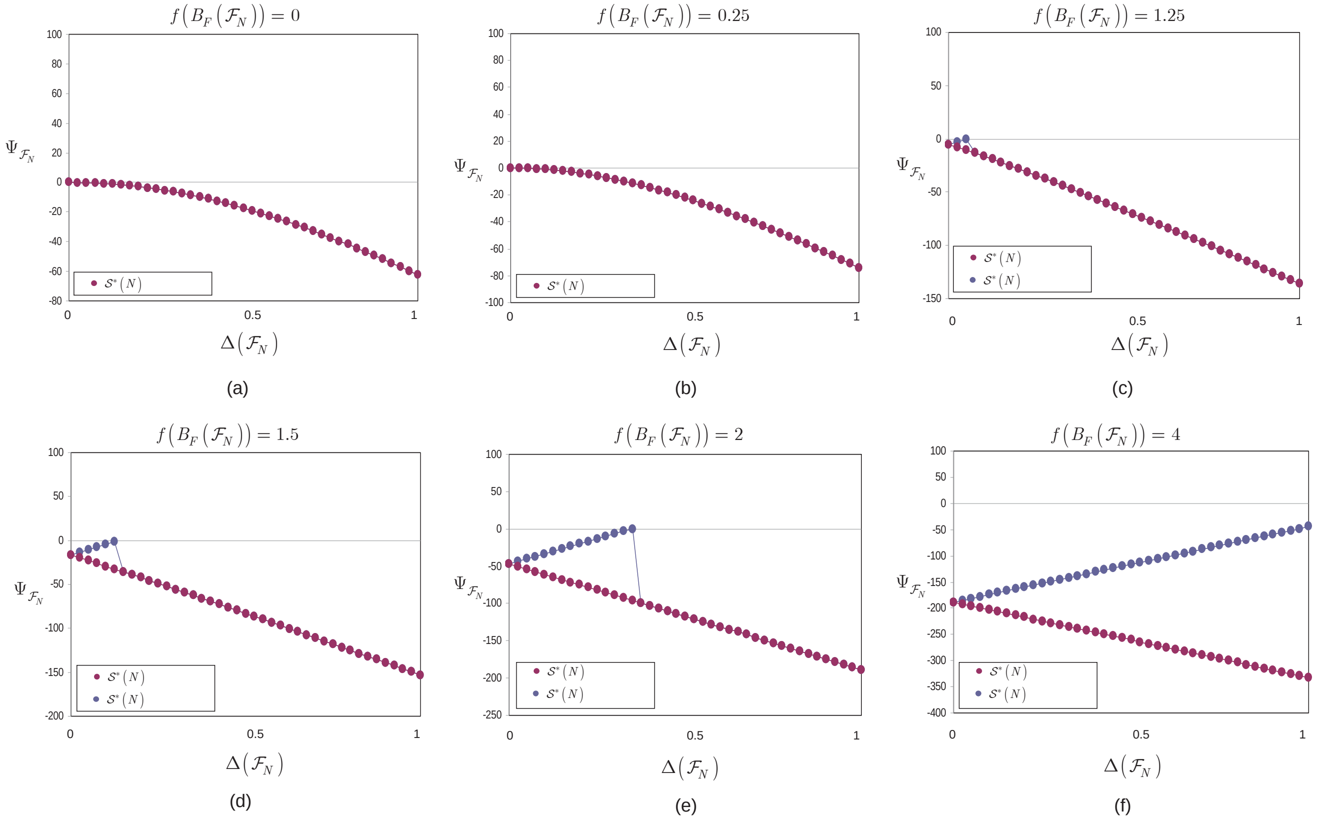

Figure A.2. The stable equilibrium states $\mathscr{S}^*(N)$ of the quantum network N at a particular $f(B_F(\mathscr{F}_N))$ in function of $\Delta(\mathscr{F}_N)$, $|V| = 100$. (a) weakly entangled quantum network, $f(B_F(\mathscr{F}_N)) = 0$. (b) weakly entangled quantum network, $f(B_F(\mathscr{F}_N)) = 0.25$. (c) strongly entangled quantum network, $f(B_F(\mathscr{F}_N)) = 1.25$. (d) strongly entangled quantum network, $f(B_F(\mathscr{F}_N)) = 1.5$. (e) strongly entangled quantum network, $f(B_F(\mathscr{F}_N)) = 2$. (f) strongly entangled quantum network, $f(B_F(\mathscr{F}_N)) = 4$.

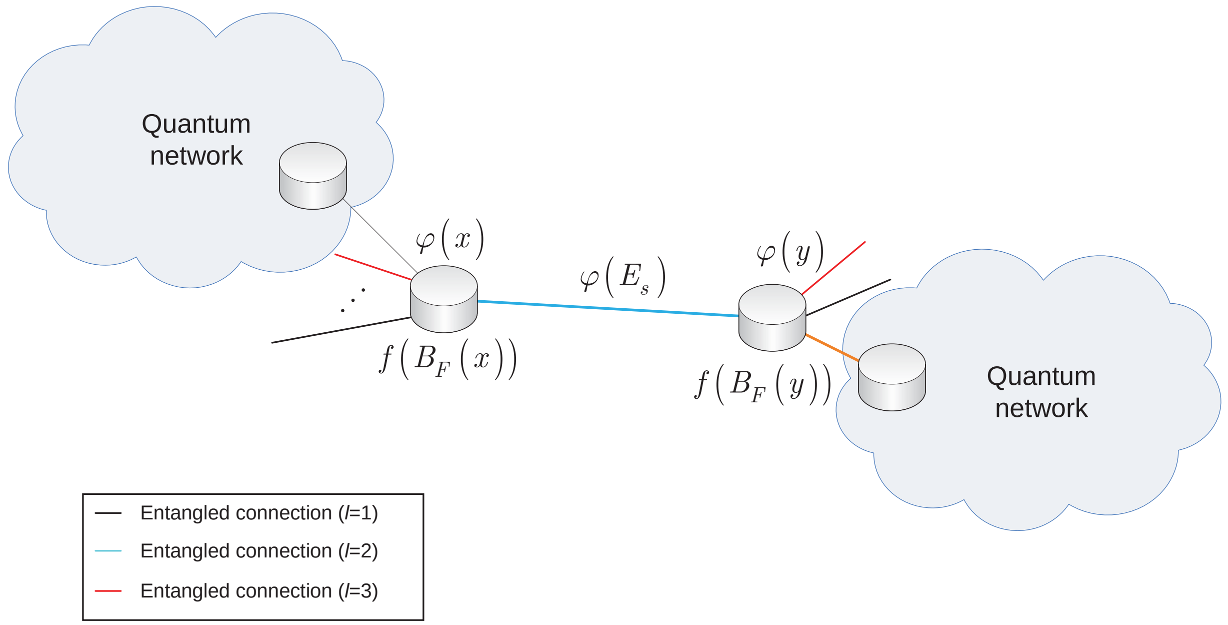

Figure A.3. Fluctuation dynamics of entangled connections in the quantum Internet. The physical entangled connections are depicted by the solid lines represent different l entanglement levels in the quantum Internet. For a given quantum node pair (x,y) with an l-level entangled connection $E_s = E(x,y)$ the fluctuation of the connection is $\varphi(E_s) = |\varphi(x) - \varphi(y)|$, where $\varphi(x)$ and $\varphi(y)$ are the node-level fluctuations. For a given t, the network state is $N(t)$, and the fluctuations of the $|V|$ quantum nodes of N are identified by $\vec{\varphi}(N(t))$ as $\vec{\varphi}(N(t)) = \left(\vec{Z}\right)^{-1/2} \left(\sum_{i=1}^{|V|} (|A_i| \exp(i\theta_i)) \exp(\pm i\alpha_i t) \vec{\gamma}_i \right), t = 1, \ldots, T$.

Proof. Since $\varphi(E_s) \leq \varphi^*(E_s)$ holds if only

$$\langle \mathscr{L}(N) \rangle = \mathscr{L}^*_{\vec{\varphi}^*(N(t))=\vec{0}}(N) + \vec{0}, \tag{A.1}$$

it follows that there exists a

$$\mathscr{L}^*(N) = \mathscr{L}^*_{\vec{\varphi}^*(N(t))=\vec{0}}(N) \tag{A.2}$$

for a given N, where $\mathscr{L}^*_{\vec{\varphi}^*(N(t))=\vec{0}}(N)$ is a target Laplacian at $\vec{\varphi}^*(N(t)) = \vec{0}$.

The $\mathscr{L}^*(N)$ target Laplacian of N is can be evaluated via $\mathscr{L}^*(N) = \left(\vec{Z}\right)^{-1} \mathscr{L}(N)$ that also determines the connection set of N such that $\vec{\varphi}^*(N(t)) = \vec{0}$. Since $\varphi^*(E_s) = 0$, the resulting quantum network keeps any $\mathscr{S}^*(N)$ equilibrium state with $f(B_F(\mathscr{S}^*(N)))$.

The proof is concluded here. ■

A.4 Notations

The notations of the manuscript are summarized in Table A.1.

Table A.1. Summary of notations.

Notation	*Description*
N	An entangled quantum network, $N = (V, E)$, where V is a set of nodes, E is a set of entangled connections.
A	A source user (quantum node) in the quantum network.
B	A destination user (quantum node).
R_i	An i-th quantum repeater, $i = 1, \ldots, \lvert V \rvert$, where $\lvert V \rvert$ is the total number of quantum nodes of N.
R_k	A next neighbor of R_k (towards destination).
l	Level of entanglement.
$\mathrm{L}_l(x,y)$	An l-level entangled connection between quantum nodes x and y, simplified as $E(x,y)$.
$d(x,y)_{\mathrm{L}_l}$	Hop-distance at an L_l-level entangled connection between quantum nodes x and y, $d(x,y)_{\mathrm{L}_l} = 2^{l-1}$.
O_C	An oscillator with frequency f_C, $f_C = 1/t_C$, serves as a reference clock.
C	A cycle, with $t_C = 1/f_C$.
π_S	A time unit, defined as $\pi_S = x t_C$, where x is the number of C.
$\mathscr{N}$	A physical quantum link of the quantum network.
B_F	Entanglement throughput [Entangled density matrices per π_S].
$\lvert B_F \rvert$	Number of entangled states [Number of Entangled density matrices].
$E(x,y)$	An l-level entangled connection between quantum nodes x and y.
$B_F(E(x,y))$	Entanglement throughput of the entangled connection $E(x,y)$ [Entangled density matrices per π_S].
$\mathscr{S}^*(N)$	Equilibrium state of the entangled quantum network N.
$\mathscr{F}_N$	Entanglement flow in N.

$\mathscr{P}_j$	A j-th entangled path of $\mathscr{F}_N$, $j=1,\ldots,Q$, where Q is the total number of paths in N.
$F_{\mathscr{P}_j}(R_i)$	Average entanglement fidelity outputted via quantum repeater R_i in the $\mathscr{F}_N$ entanglement flow of N.
σ_f	An f-th entangled subsystem outputted by R_i, $f=1,\ldots,n$.
$F_{\mathscr{P}}(R_i)$	Average entanglement fidelity outputted via quantum repeater R_i in the $\mathscr{F}_N$ entanglement flow of N.
$F(\mathscr{F}_N)$	The average fidelity of entanglement flow $\mathscr{F}_N$.
$\left\|V_{\mathscr{P}_j}\right\|$	Number of quantum nodes of a given entangled path $\mathscr{P}_j$.
$\left\|S_{\mathscr{P}_j}\right\|$	Number of entangled connections nodes of a given entangled path $\mathscr{P}_j$.
$B_{F,\mathscr{P}_j}(\mathscr{F}_N)$	Average entanglement rate of $\mathscr{P}_j$ of $\mathscr{F}_N$ at a particular entanglement fidelity F.
$B_{F,\mathscr{P}_j}(E_s)$	Average entanglement throughput of an s-th entangled connection E_s for a particular entanglement fidelity F, $s=1,\ldots,\left\|S_{\mathscr{P}_j}\right\|$.
$B(\mathscr{F}_N)$	Average entanglement throughput of $\mathscr{F}_N$ for the total Q paths of N, for a particular entanglement fidelity F.
$\Delta(\mathscr{F}_N)$	Average noise of entanglement flow $\mathscr{F}_N$, $0\leq\Delta(\mathscr{F}_N)\leq 1$.
$\Delta(R_i)$	Average noise of an i-th quantum node R_i.
$\tilde{\mathscr{F}}_N$	A seamless entanglement flow $\mathscr{F}_N$ in N.
$\varphi(E_s)$	Fluctuation of an entangled connection E_s of $\mathscr{F}_N$, $\varphi(E_s)=\|\varphi(x)-\varphi(y)\|$, where $\varphi(x)$ and $\varphi(y)$ are the fluctuations associated with quantum nodes x and y.
$\varphi^*(E_s)$	An upper bound on $\varphi(E_s)$ of an entangled connection E_s in a $\mathscr{S}^*(N)$ stable equilibrium state.
$\mathscr{F}_N^*$	A seamless optimal entanglement flow $\mathscr{F}_N$ in N.
$\mathscr{S}_N'$	A weakly entangled subnetwork of N with $\|\mathscr{S}_N\|$ quantum nodes.
$\Omega_{\mathscr{S}_N'}$	Number of paths of a $\mathscr{S}_N'$ weakly entangled structure.
$B_F(\mathscr{S}_N')$	Average entanglement throughput of $\mathscr{S}_N'$ for a particular entanglement fidelity F.
$B_F^*(\mathscr{S}_N')$	Expected value of $B_F(\mathscr{S}_N')$ for a particular entanglement fidelity F.
$\mathscr{S}_N^*$	A strongly entangled subnetwork of N.
$\Psi_{\mathscr{F}_N}$	Stability function of the entangled structure N.
$F(\mathscr{F}_N)$	Average fidelity of entanglement flow $\mathscr{F}_N$.
$\phi(\cdot)$, $f(\cdot)$	Normalizing functions.
c_B	A constant.
φ	A constant.
$\mathscr{S}(N)$	Statistical physics model of an entangled quantum network N.
$\phi(F(\mathscr{F}_N))$	Normalized value of $F(\mathscr{F}_N)$.
$f(B_F(\mathscr{F}_N))$	Normalized value of $B_F(\mathscr{F}_N)$.
$H(\mathscr{F}_N)$	Hamiltonian of the entanglement flow $\mathscr{F}_N$ in the entangled network structure N.
$J_{i,k}$	An interaction parameter.

$H(\Delta(\mathscr{F}_N))$	Hamiltonian of the average noise $\Delta(\mathscr{F}_N)$ of $\mathscr{F}_N$.
μ_0	A normalization term.
σ_i	A state of quantum node.
$\Delta F_{\mathscr{P}}(R_i)$	A fidelity difference, $\Delta F_{\mathscr{P}}(R_i) = F^*_{\mathscr{P}}(R_i) - F_{\mathscr{P}}(R_i)$.
ξ_i	Model parameter, $\xi_i = \text{sign}(\Delta F_{\mathscr{P}}(R_i))$, where the sign$(x)$ function returns the sign of x (sign(0) is considered as negative.
$H(\mathscr{F}_N)$	Hamiltonian of entanglement flow $\mathscr{F}_N$.
$\tilde{\xi}(\mathscr{F}_N)$	Average of model parameter ξ_i, $\tilde{\xi}(\mathscr{F}_N) = \frac{1}{\|V\|}\sum_{i=1}^{\|V\|}\xi_i$.
$\tilde{K}$	Average number of entangled connections between the nodes.
$E(\mathscr{F}_N)$	Energy of the system $\mathscr{S}(N)$.
$S_e(\mathscr{F}_N)$	Entropy of $\mathscr{S}(N)$.
$f(\xi_i)$	A distribution function.
$N(t)$	State of the entangled structure N at a particular t, $t = 1, \ldots, T$.
$\Pr\left(\tilde{\xi}(\mathscr{F}_N) \middle\| \tilde{\xi}'(\mathscr{F}_N)\right)$	Probability of transition $\tilde{\xi}'(\mathscr{F}_N) \to \tilde{\xi}(\mathscr{F}_N)$ at a given state $\mathscr{S}(N(t))$.
$\Pr\left(\tilde{\xi}'(\mathscr{F}_N) \middle\| \tilde{\xi}(\mathscr{F}_N)\right)$	Probability of transition $\tilde{\xi}(\mathscr{F}_N) \to \tilde{\xi}'(\mathscr{F}_N)$ at a given $\mathscr{S}(N(t))$.
$\mathscr{S}(N(t))$	Statistical physics model of an entangled quantum network $N(t)$.
$\Phi\left(\xi_i, \tilde{\xi}(\mathscr{F}_N)\right)$	Distribution function at a particular $\tilde{\xi}(\mathscr{F}_N)$.
ω	A normalization term.
$\Omega(\mathscr{F}_N)$	A coefficient of the stability analysis of the entangled structure N.
$\zeta(\mathscr{F}_N)$	A coefficient of the stability analysis of the entangled structure N.
$\kappa_{\mathscr{F}_N}\left(\tilde{\xi}(\mathscr{F}_N)\right)$	A coefficient of the stability analysis of the entangled structure N.
$\Pi_{\mathscr{F}_N}\left(\tilde{\xi}(\mathscr{F}_N)\right)$	A coefficient of the stability analysis of the entangled structure N.
$\chi(\mathscr{F}_N)$	A component of the $\Psi_{\mathscr{F}_N}(\phi(F(\mathscr{F}_N)))$ stability function of the entangled quantum network N.
$\Psi'_{\mathscr{F}_N}(\phi(F(\mathscr{F}_N)))$	Derivative the $\Psi_{\mathscr{F}_N}(\phi(F(\mathscr{F}_N)))$ stability function of the entangled quantum network N.
$\Psi''_{\mathscr{F}_N}(\phi(F(\mathscr{F}_N)))$	Second derivative the $\Psi_{\mathscr{F}_N}(\phi(F(\mathscr{F}_N)))$ stability function of the entangled quantum network N.
λ	A coefficient of the stability analysis of the entangled structure N.
ς	A coefficient of the stability analysis of the entangled structure N.
Γ	A coefficient of the stability analysis of the entangled structure N.
P_{R_i}	Entanglement purification in a quantum node R_i.
$P_{\mathscr{S}_N}$	Entanglement purification in a subnetwork $\mathscr{S}_N$ of N.
P_N	Entanglement purification in N.
$M(\mathscr{S}_N)$	Number of $\mathscr{S}_N$ subnetworks of N.
$\mathscr{S}_N^{(z)}$	A z-th subnetwork of N.
$\mu_N(\mathscr{S}_N, \Delta(\mathscr{F}_N))$	An averaged fidelity for a given $\mathscr{S}_N$, at a particular $\Delta(\mathscr{F}_N)$.

$\delta(R_i)$	State of an i-th quantum repeater R_i.
$B_F(P_{R_i})$	Entanglement rate consumption of entanglement purification P_{R_i} (sum of incoming and outcoming entanglement rates in R_i associated with P_{R_i}).
$S_{P_{R_i}}$	Set of entangled connections of R_i associated with entanglement purification P_{R_i}.
$\left\lvert S_{P_{R_i}} \right\rvert$	Cardinality of set $S_{P_{R_i}}$.
$F^*_{\mathscr{P}}(R_i)$	A target average fidelity of R_i.
$F_{\mathscr{P}}(R_i)$	An average fidelity of R_i.
$B^*_F(P_{R_i})$	A target value of $B_F(P_{R_i})$, $B^*_F(P_{R_i}) < B_F(P_{R_i})$.
ω	A ratio of the target average fidelity $F^*(\mathscr{F}_N)$ of the entanglement flow $\mathscr{F}_N$ of N and $F^*_{\mathscr{P}}(R_i)$.
$\mathscr{C}(R_i)$	Capability of a quantum repeater R_i to improve the $F(\mathscr{F}_N)$ average fidelity of $\mathscr{F}_N$ to a target $F^*(\mathscr{F}_N)$ via an entanglement purification P_{R_i}.
$\mathscr{C}(N)$	Capability of the entangled network N to improve the $F(\mathscr{F}_N)$ average fidelity of $\mathscr{F}_N$ to a target $F^*(\mathscr{F}_N)$ via P_{R_i} in the $\lvert V\rvert$ nodes, $i = 1,\ldots,\lvert V\rvert$, of N.
$B_F(P_N)$	Total entanglement rate consumption of entanglement purification P_N in N.
$\tilde{B}_F(P_{R_i})$	Average entanglement rate consumption at P_N for a given node.
P'_{R_i}	Entanglement purification in a local R_i with an increased target entanglement rate consumption $B^*_F\left(P'_{R_i}\right)$.
$B_F(P'_N)$	Total entanglement rate consumption of entanglement purification P'_N.
$\tilde{B}_F\left(P'_{R_i}\right)$	Average entanglement rate consumption of P'_N.
∂	A capability of the entangled network N for fidelity improvement, $\mathscr{C}''(N) = \mathscr{C}'(N)\,\partial$, where $\partial = \frac{\mathscr{C}''(N)}{\mathscr{C}'(N)} = \left(1 - X^2\mu^2\right)$, where $X > 0$ and $\mu > 0$.
$\mathscr{L}(N)$	A symmetric $\mathscr{L}(N)$ Laplacian of the undirected entangled quantum network N.
$\mathscr{L}^*(N)$	An asymmetric and symmetrizable Laplacian of N.
$\langle\mathscr{L}(N)\rangle$	A general Laplacian $\langle\mathscr{L}(N)\rangle$ of a directed entangled quantum network N.
$\vec{\varphi}(N)$	A vector of node fluctuations in N.
λ	An eigenvalue of $\mathscr{L}^*(N)$.
$S(\mathscr{L}(N))$	A scaled Laplacian $\mathscr{L}(N)$.
ω_{ik}	Total scaled entanglement throughput of all paths over $E_s(R_i,R_k)$, $\omega_{ik} > 0$.
z_i	A constraint for R_i, such that the symmetry condition $z_i\omega_{ik} = z_k\omega_{ki}$ holds, where z_k is a constraint for R_k.
$\vec{Z}$	A vector, defined for the $\lvert V\rvert$ quantum nodes, as $\vec{Z} = \mathrm{diag}\left(z_1,\ldots,z_{\lvert V\rvert}\right)$.
$\vec{\gamma}_i$	An orthonormal eigenvector associated with an i-th eigenvalue λ_i (eigenbasis of $S(\mathscr{L}(N))$), $i = 1,\ldots,\lvert V\rvert$.
δ_{uv}	Kronecker delta.
$B^*_F(\mathscr{F}_N)$	Critical bound on $B_F(\mathscr{F}_N)$ in $\mathscr{S}^*(N)$.
$\zeta_{\mathscr{L}(N)}$	A residual Laplacian.
$\vec{0}$	A null matrix.

C_d	A constant, $C_d \geq 0$.
$A_i(t)$	A coefficient to evaluate the $\varphi(E_s)$ fluctuation parameter of an entangled connection E_s of $\mathscr{F}_N$, $A_i(t) = \tau_i \exp(\pm i\alpha_i t)$, where $\tau_i = \|A_i\| \exp(i\theta_i)$.
θ_i	A coefficient to evaluate the $\varphi(E_s)$ fluctuation parameter of an entangled connection E_s of $\mathscr{F}_N$, $-\pi < \theta_i \leq \pi$.
α_i	A coefficient to evaluate the $\varphi(E_s)$ fluctuation parameter of an entangled connection E_s of $\mathscr{F}_N$, $\alpha_i = \sqrt{\lambda_i}$.
